# Effects of Eribulin on the RNA Content of Extracellular Vesicles Released by Metastatic Breast Cancer Cells

**DOI:** 10.3390/cells13060479

**Published:** 2024-03-08

**Authors:** Matteo Giulietti, Francesco Piva, Monia Cecati, Serena Maggio, Michele Guescini, Tiziana Saladino, Laura Scortichini, Sonia Crocetti, Miriam Caramanti, Nicola Battelli, Emanuela Romagnoli

**Affiliations:** 1 Department of Specialistic Clinical and Odontostomatological Sciences, Polytechnic University of Marche, 60131 Ancona, Italy; 2 Department of Biomolecular Sciences, University of Urbino Carlo Bo, 61029 Urbino, Italy; 3 Oncology Unit AST3, Macerata Hospital, Via Santa Lucia 2, 62100 Macerata, Italy

**Keywords:** extracellular vesicles (EVs), microtubule-targeting agents (MTAs), eribulin, RNA-seq, miRNAs

## Abstract

Extracellular vesicles (EVs) are small lipid particles secreted by almost all human cells into the extracellular space. They perform the essential function of cell-to-cell communication, and their role in promoting breast cancer progression has been well demonstrated. It is known that EVs released by triple-negative and highly aggressive MDA-MB-231 breast cancer cells treated with paclitaxel, a microtubule-targeting agent (MTA), promoted chemoresistance in EV-recipient cells. Here, we studied the RNA content of EVs produced by the same MDA-MB-231 breast cancer cells treated with another MTA, eribulin mesylate. In particular, we analyzed the expression of different RNA species, including mRNAs, lncRNAs, miRNAs, snoRNAs, piRNAs and tRNA fragments by RNA-seq. Then, we performed differential expression analysis, weighted gene co-expression network analysis (WGCNA), functional enrichment analysis, and miRNA-target identification. Our findings demonstrate the possible involvement of EVs from eribulin-treated cells in the spread of chemoresistance, prompting the design of strategies that selectively target tumor EVs.

## 1. Introduction

Breast cancer is the most frequent malignancy in women. Epidemiological data show that in 2020, there were 2.3 million women diagnosed with breast cancer and 685 thousand deaths globally [1]. Breast cancer seems to develop starting from certain pre-existing lesions such as atypical ductal hyperplasia (ADH), atypical lobular hyperplasia (ALH), lobular carcinoma in situ (LCIS), ductal carcinoma in situ (DCIS), and flat epithelial atypia (FEA) [2]. Unfortunately, it is impossible to predict which lesion is most likely to progress into invasive carcinoma. Indeed, cancer progression depends on the heterogeneous genetic mutations that occurred in the cells [3,4] and the interactions, not yet well characterized, between tumor epithelial cells and cells comprising the microenvironment (myoepithelial and endothelial cells, fibroblasts, myofibroblasts, leukocytes, and other cell types) [5]. Triple-negative breast cancer (TNBC) is an aggressive breast cancer subtype characterized by the absence of the expression of receptors for estrogen (ER), progesterone (PR), and of human epidermal growth factor receptor-2 (HER2) [6]. Once diagnosed, treatment of TNBC relies on chemotherapy, in particular, on the anthracycline and taxane drugs [7], also in combination with other single agents such as gemcitabine [8] or capecitabine after surgery [9]. Generally, the median overall survival (OS) in patients with early stages of TNBC treated with chemotherapy is 13–18 months [10]. Research is focused on developing new drugs which can improve the clinical outcomes of TNBC-positive patients.

Eribulin mesylate (E7389; Halaven) is a non-taxane microtubule dynamics inhibitor responsible for the arrest of the cell cycle in the G2-M phase; therefore, the metaphase/anaphase checkpoint is not passed, and the cell undergoes apoptosis [11,12]. In particular, eribulin inhibits microtubule polymerization by binding β-tubulin at the exposed (plus) ends of growing microtubules, leading to the blockage of mitotic spindle formation [13]. Moreover, eribulin has nonmitotic effects in the form of the reversal of the epithelial-to-mesenchymal (EMT) transition [14] and suppression of cancer cell migration, invasion and metastasis [15,16,17]. An effect exists in the form of vascular remodeling, which is an increased tumor perfusion that eliminates hypoxia-driven growth aggressiveness and increases exposure of subsequent treatments [16]. In addition, eribulin induces the caspase-independent apoptosis pathway by triggering Bcl-2 phosphorylation [18]. The antiproliferative effect of eribulin is enhanced by stathmin, a phosphoprotein that modulates microtubule dynamics [19]; therefore, the stathmin could be dosed to predict its efficacy. Eribulin is extremely effective in TNBC patients [20], where it is used after treatment or in combination with anthracycline and taxane [21,22] or, more recently, with gemcitabine [23]. Of note, it was shown that there is a minimal risk of drug–drug interactions in the clinical setting [24]. Further details about its mechanisms of action and results of clinical trials have been reviewed elsewhere [25,26].

Extracellular vesicles (EVs) are small lipid particles secreted from almost all human cell types, both healthy and malignant, that deliver their cargo (i.e., DNA, proteins, various types of RNAs) to recipient cells [27,28]. In this way, EVs act as mediators of the cell-to-cell communication and cancer progression. Experimental studies in breast cancer demonstrated the role of EVs in promoting cell growth and survival [29], metastasis [30], epithelial to mesenchymal transition [31], angiogenesis [32], immunosuppression [33], and chemoresistance [34,35]. These effects are mainly due to the delivery of miRNA to the recipient cell [34]. Interestingly, the EV cargo varies with cell conditions, therefore also following the drug treatment. In fact, Pederson et al. demonstrated that eribulin modifies the cargo of EVs isolated from eribulin-treated breast cancer cells. In particular, in EVs of HCC1937 TNBC cells, eribulin significantly decreased integrin-linked kinase (ILK) level [36], a driver of EMT in recipient mammary epithelial cells [37]. Moreover, since the secretion of EVs is based on microtubule-dependent trafficking, microtubule-targeting agents (MTAs) used in the treatment of breast cancer could affect EVs formation, cargo and release. In fact, in MDA-MB-231 and HCC1937 triple-negative breast cancer (TNBC) cells treated with eribulin for 2–4 h, while the number of vesicles released remained constant, those exposing CD63 decreased [36]. CD63 tetraspanin is a characteristic EV-associated protein and related to endosomal sorting and EV cargo loading.

It is known that the released EVs from MDA-MB-231 breast cancer cells treated with paclitaxel, another MTA, promote the chemoresistance associated with a specific EV cargo, which can influence other cells and can potentially serve as a marker of MTA resistance [38]. Accordingly, it is important to understand if a treatment could contribute to the propagation of biologically meaningful messages (e.g., drug resistance) to neighboring and distal cells. Here, we assessed the RNA content of EVs released by highly aggressive, invasive and poorly differentiated triple-negative, MDA-MB-231 breast cancer cells treated with eribulin in comparison with the content of EVs from untreated cells. Our results highlight the potential contribution of EVs of eribulin-treated cells in eribulin resistance, epithelial-to-mesenchymal transition, adhesion, extracellular matrix (ECM) remodeling, lipid metabolism, and chromosomal instability.

## 2. Materials and Methods

### 2.1. Cell Cultures

The MDA-MB-231 human breast cancer cell line was obtained from ATCC (Manassas, VA, USA), and it was grown in L-15 (Leibovitz) (ThermoFisher, Milan, Italy) medium supplemented with 1% penicillin/streptomycin, 2 mM glutamine and 10% heat-inactivated fetal bovine serum. L-15 medium is used to grow cell lines in the absence of CO_2_-enriched atmosphere. Cell culture media were changed every two days. MDA-MB-231 cells were treated with eribulin at 1.6, 2.5 and 5 nM, and cell viability was monitored using MTT assay.

### 2.2. Extracellular Vesicle Isolation

EV isolation was carried out in accordance with our previous studies [39,40]. The day before the collection of EVs, we replaced the medium with fresh medium containing vesicle-depleted FBS, produced by ultracentrifugation overnight at 100,000× *g* at 4 °C, and the resulting supernatant was filtered through 0.4 μm membranes before being used for the preparation of EVs. Cell-conditioned medium was collected and purified by two serial centrifugations for 15 min at 1000× *g* and 15 min at 2000× *g* to remove debris and apoptotic bodies. Then, to remove large EVs, the supernatant was centrifuged at 12,000× *g* for 30 min and 15,000× *g* for 30 min. To isolate small EVs, the collected supernatant was subjected to ultracentrifugation at 100,000× *g* for 2 h. The crude small EV pellet was resuspended in a large volume of PBS and further ultracentrifuged at 100,000× *g* for 1 h to wash the sample. During the whole protocol, temperatures was maintained at 4 °C.

### 2.3. Nanoparticle Tracking Assay (NTA)

NTA measurements were performed, in accordance with our previous studies [40], with a NanoSight LM10 (NanoSight, Amesbury, UK), and three videos of either 30 s or 60 s were recorded of each sample. All measurements were performed at room temperature, never above 25 °C. The NTA 3.1 software (Nanosight, Amesbury, UK) was used for capturing and analyzing the data, which are presented as the mean ± SD of the three video recordings. Samples containing high particle numbers were diluted before analysis, and the relative concentration was then calculated according to the dilution factor. Beads with 100 nm and 400 nm diameters (Malvern Instruments Ltd., Malvern, UK) were used as control.

### 2.4. Western Blotting Analysis

For SDS-PAGE, samples containing 10 µg of protein were mixed with Laemmli sample buffer (1:1 ratio) and loaded onto 12% SDS-PAGE gels. Subsequently, proteins were blotted to a Polyvinylidene difluoride (PVDF) membrane (ThermoFisher, Milan, Italy). Primary antibodies used were CD63 (cat. 10628D, ThermoFisher), CD9 (cat. #13174, CellSignalling, Danvers, MA, USA) and calnexin (cat. C4731, Merk/Sigma, Milan, Italy). Primary antibodies were incubated overnight at 4 °C, followed by washing and the application of secondary HRP-conjugated antibody (ThermoFisher, Milan, Italy). Immune complexes were visualized using the Clarity and/or Clarity Max (Bio-Rad, Milan, Italy).

### 2.5. RNA Sequencing and Data Analysis

Total RNAs were isolated from small EVs of untreated and eribulin-treated cells using Total RNA Purification Kit (Cat. 17200, Norgen, Thorold, ON, Canada) following the manufacturer’s instructions. RNA integrity was assessed using an RNA Nano 6000 Assay Kit with the Bioanalyzer 2100 system (Agilent Technologies, Santa Clara, CA, USA). The cDNA library for mRNAs and lncRNAs was developed using the SMARTer Stranded V3 (Takara Bio, Kusatsu, Japan) with ribodepletion; then, the sequencing of this library was performed using Next Generation Sequencing (NGS) Illumina NovaSeq platform to generate 150 bp paired-end reads. The small RNA transcriptome was evaluated by preparing the library (QIAseq miRNA kit, Qiagen, Milan, Italy) and by sequencing with the same platform obtaining 150 bp paired-end reads.

We have not performed the validation of the expression of a sample of genes as it is emerging that the RNA sequence results are very reliable or consistent with those obtained with the RT-qPCR technique [41].

The Unique Molecular Indices (UMIs), embedded in the reads by the kits for preparation of libraries for a more accurate transcript quantification, were detected and removed together with the adapters using the UMI-tools v1.0.0 software [42]. Then, after quality controls by FastQC tool version 0.12.0 (Babraham Bioinformatics, Cambridge, UK) (http://www.bioinformatics.babraham.ac.uk/projects/fastqc/, accessed on 10 July 2023), reads were mapped to the human genome (GRCh38 assembly) using the alignment tool Hisat2 v2.2 [43] with default parameters. Then, UMI-tools software was adopted also for read deduplication, which occurs during PCR amplification, based on the mapping coordinates. HTSeq v1.99.2 [44], basing on the Gencode v38 annotation GTF file (based on the GRCh38 human assembly) for the coordinates of protein coding genes, lncRNAs and snoRNAs, was used to count the deduplicated reads mapped to each gene. Additional GTF files containing the genomic coordinates of miRNAs, piRNAs and tRNA-derived ncRNAs have been retrieved from miRBase v22.1 [45], piRBase [46] and tRFexplorer [47], respectively.

By using the DEBrowser tool [48], removal of low-count (<10) genes and the Trimmed Mean of the M-values (TMM) normalization were performed in order to carry out the subsequent Principal Component Analysis (PCA) and Hierarchical Clustering analysis. Differential expression analysis was carried out using DESeq2 tool [49], adopting the following thresholds: |Log2(FC)| ≥ 1 and BH-adjusted *p*-value ≤ 0.05. The lists of differentially expressed genes (up- and down-regulated) were submitted to Enrichr tool [50] to highlight enriched KEGG pathways, MSigDB Hallmarks, Panther, HumanCyc, Reactome, Elsevier Pathway Collection, WikiPathway, BioPlanet, and GeneOntology terms. Only significant results (adjusted *p*-values < 0.05, Fisher’s exact test) are reported and discussed in this study. The software used for data processing has consulted multiple pathway libraries; therefore, there is no single and univocal result in the tables.

### 2.6. Analysis of Co-Expressed Gene Modules by WGCNA

Firstly, we removed very low-expressed genes (we retained genes with read count > 10, in more than 75% of the samples) in order to limit noise, which would affect the following analyses. The selected genes (*n* = 12,209) have been submitted to weighted gene co-expression network analysis (WGCNA, by using the R package WGCNA v1.72 [51] following the standard WGCNA procedures [52], in order to identify the modules most related to the eribulin treatment. By the “pickSoftThreshold” function, the soft threshold β was selected when the R^2^ of the Scale Free Topology Model was >0.8. Indeed, accordingly to WGCNA authors [52], when this R^2^ is >0.8 for low powers (<30), it means that the topology of the network is scale-free and there are no batch-effects in the data. The identification of modules was carried out by the creation of an adjacency matrix transformed into a topological overlap matrix (TOM), which was subsequently submitted to hierarchical clustering performed by the function cutreeHybrid from the R package dynamicTreeCut v1.63 (parameters: minimum module size = 30 genes; similar modules merged when cuttree height < 0.25). In each module, WGCNA identifies the Module Eigengene (ME, summarizing the whole module gene expression) and calculates the Module Membership (MM) of each gene, which is obtained by correlation analysis between the expression level of this gene and the ME. Genes with the highest MM scores are considered hub genes of the module [53]. Module-to-trait relationships were identified using Pearson’s correlation analysis, which evaluates the correlations between MEs and a trait, which in this study is the treatment status (eribulin or control) and gives back a Gene Significance (GS) score for each gene. For each module significantly associated with the eribulin treatment, the top 10 genes with best GS scores are reported.

### 2.7. Analysis of miRNA Target Genes

Instead of predicting the targets of our DE miRNAs (|log2FC| > 1.5), which would have led to a high number of false-positive predictions, we wanted to consider only experimentally validated target genes. The most up-to-date database containing these experimental interactions is mirTarbase (v9, 2022, [54]). In particular, we considered only miRNA-target interactions validated by well-established techniques, such as gene reporter assay, Western blot, and RT-qPCR, since they are the most reliable methods to demonstrate direct interaction between an miRNA and its target, while methods like microarray or high-throughput sequencing provide indirect relationships [54]. Even the mirTarbase tool refers to the former techniques as ‘strong evidence’ methods. Among validated targets, we searched for genes involved in chromosomal instability (gene list available at [55]), multi-drug resistance (ABC pumps: *ABCB1*, *ABCC1*, *ABCC2*, *ABCG2*), and immune checkpoints (gene list available at [56]).

### 2.8. Association of piRNAs, snoRNAs and tRNA Fragments with Breast Cancer

We investigated possible roles of piRNAs, snoRNAs and tRNA fragments in breast cancer by using disease-association prediction tools. Regarding piRNAs, we used iPiDi-PUL, a recently developed tool to predict piRNA–disease associations via positive unlabeled learning [57], and we filtered the results with a confidence score >0.8. For snoRNAs, we used the RNADisease v4.0 tool, which assigns a confidence score to ncRNA-disease association by integrating the experimental and prediction evidence [58] and filtered results associated with breast cancer with a confidence score >0.6. Regarding tRNA fragments, we used tRFExplorer [47], a database containing tRNA fragment expression data from TCGA.

### 2.9. RNA Isolation and Quantification

For miRNA analyses, total RNA was extracted from control and 1.6 nM Eribulin-treated MDA cells by using the miRNeasy Cells Advanced Kit (Qiagen, Milan, Italy) and reverse transcribed with the miRCURY LNA Universal RT microRNA PCR kit (Qiagen, Milan, Italy). The optional UniSP6 RNA spike-in oligonucleotide was added to each reaction and used for normalization. Quantitative RT-PCRs were performed with the miRCURY LNA SYBR^®^ Green PCR Kit and miRCURY LNA miRNA PCR Assays (Qiagen, Milan, Italy) specific for the miRNAs analyzed. The qRT-PCR conditions were the following: initial incubation step at 95 °C for 10 min followed by 50 cycles consisting of two steps at 95 °C for 10 s and 60 °C for 60 s. Cq were determined using the Cy0 method, performed according to the ΔCq method [59].

## 3. Results

### 3.1. EV Isolation and Characterization

**Figure 1:**
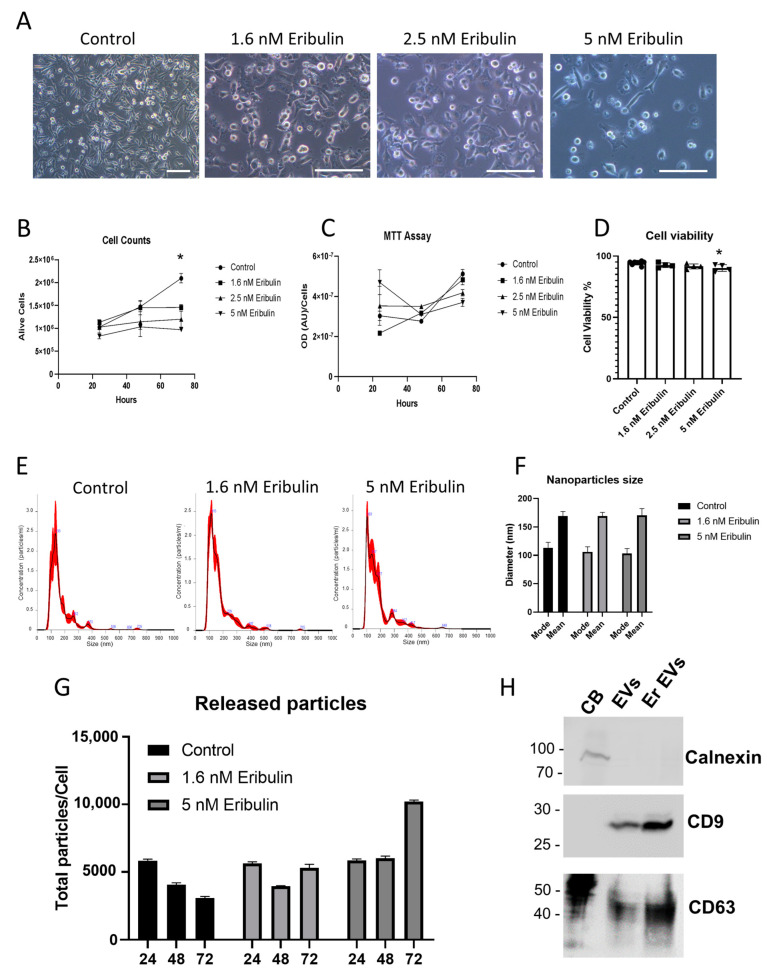
Characterization of EVs released in presence of increasing concentrations of eribulin. Cells treated with eribulin were analyzed by light microscopy. Scale bar = 100 µm (**A**). The number of viable cells was evaluated with trypan blue (**B**) and MTT (**C**) assays, and viability data reported as percentage (**D**). EVs released following 1.6 nM and 5 nM eribulin treatment for 72 h were analyzed using nanoparticle tracking assay, distribution plots of the calculated hydrodynamic diameter (**E**), summarized in the relative mean/mode graph (**F**), and vesicle quantifications (**G**) were reported. Western blot analysis against the well-established EV markers CD63 and CD9, and against calnexin, a well-established endoplasmic reticulum marker, was reported for cell bodies (CB), control (EVs) and 1.6 nM eribulin treated (Er EVs) EVs (**H**). * *p* < 0.05.

MDA-MB-231 cells were treated with increasing concentration of eribulin 1.6 nM, 2.5 nM and 5 nM for 72 h; subsequently, cell viability and morphology were monitored in order to determine the optimal eribulin concentration to administer in an attempt to achieve the stronger effect on morphology without significant cell death. Following eribulin treatment, MDA-MB-231 cells progressively became more and more round in shape as shown by light microscopy analysis (Figure 1A). This adaptation was concentration-dependent and suggests a deep rearrangement of cellular cytoskeleton. Trypan blue assays revealed that cell viability significantly decreased after 3 days of eribulin treatment at 2.5 and 5 nM (Figure 1B). Moreover, MTT assay revealed that 2.5 and 5 nM eribulin treatments induced a reduction in cell viability (Figure 1C). Figure 1D shows that the viability slightly decreased with increasing eribulin concentrations used; however, statistical significance was reached only in the presence of 5 nM eribulin (ANOVA test, post-hoc Dunnet’s test, * *p* < 0.05). The day before EV collection, cells were washed and switched to a fresh medium containing vesicle-depleted FBS and eribulin. After another 24 h incubation, conditioned medium was collected to isolate secreted EVs by using a standard serial-ultracentrifugation protocol. The nanoparticle tracking assay (NTA) of purified EVs showed that vesicles have a hydrodynamic compatible with that of small EVs (30–200 nm); moreover, the NTA clearly showed that EV size distribution was quite similar in all the tested conditions (Figure 1E). In particular, the mean and mode of the particle’s hydrodynamic diameter are summarized in Figure 1F. This graph shows a slight but not significant decrease in the mode of the diameter of 5 nM eribulin-treated EVs. The quantification of released EVs showed that 5 nM eribulin induced a strong release of particles (Figure 1G), which could be at least in part the result of passive leakage from the plasma membrane of damaged cells. Finally, Western blot analysis of the isolated EVs showed the absence of contamination by cellular components and the successful removal of cellular debris. Indeed, calnexin, a well-known endoplasmic reticulum marker, was only found in the cell body (CB) lane. In contrast, EV samples were negative for calnexin, but positive for EV markers CD63 and CD9, demonstrating no carryover of cellular organelles into the EV pellet (Figure 1H). Based on these data, the eribulin concentration used to treat MDA-MB-231 in the following RNA characterization experiments was 1.6 nM eribulin for 72 h. The chosen concentration is effective, according to European Medicines Agency (EMA) [60], and similar experimental conditions (MDA-MB-231 exposed to 72 h with 1.5 nM eribulin) have been adopted elsewhere [61].

### 3.2. RNA Sequencing and Data Analysis

**Figure 2:**
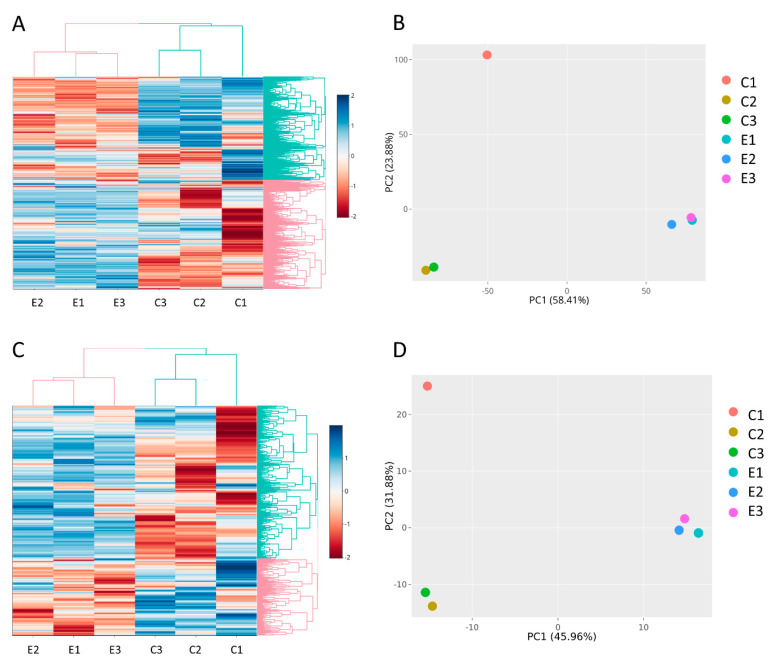
(**A**) Cluster analysis and (**B**) PCA of long RNA library. (**C**) Cluster analysis and (**D**) PCA of small RNA library. The graphs highlight the separation among untreated (C1, C2, C3) and eribulin-treated (E1, E2, E3) MDA-MB-231-derived EV samples.

To understand the cargo changes of EVs released from triple-negative breast cancer cell line MDA-MB-231 following eribulin treatment, we performed RNA sequence analysis of EV content from three non- and three eribulin-treated biological replicates. The quantity and quality of RNA from EVs are shown in Table S1 and Figure S1. In all samples, as expected for RNA isolated from EVs, small RNA peaks (25–200 nt) were present, and the level of 28S and 18S rRNA (>1000 nt) is very low. Two libraries have been produced for RNA sequence analysis, i.e., for long RNAs (mRNAs, lncRNAs) and for small RNAs (miRNAs, piRNAs, snoRNAs, tRNA-derived ncRNAs). After quality control, preprocessing of RNA sequence data has been performed by the removal of low-expressed genes and by expression normalization (Supplementary Figure S2, Supplementary Tables S2 and S3). As expected, cluster analysis and PCA showed a clear separation between untreated (samples C1, C2, C3) and eribulin-treated samples (i.e., E1, E2, E3) regarding both the analyses for long RNAs (Figure 2A,B) and for small RNAs (Figure 2C,D). This demonstrates that the replicates of the treated samples are transcriptionally similar to each other and distinct from the control group. However, it can be noted that, strangely, the C1 sample is quite different from C2 and C3, but it is still included in the group of controls.

**Figure 3:**
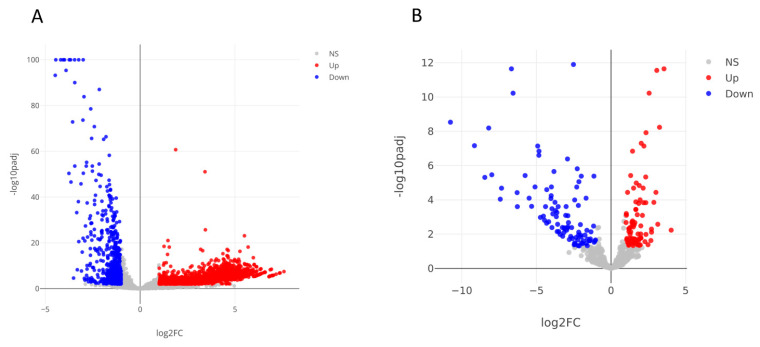
Volcano plots resulted from the differential expression analysis performed using DESeq tool and the following thresholds: |Log_2_(FoldChange)| ≥ 1, adjusted *p*-values < 0.05. Colored dots are differentially expressed mRNAs and lncRNAs (**A**). Differentially expressed miRNAs, piRNAs, snoRNAs and tsRNAs (**B**). NS: not significant.

Regarding mRNAs and lncRNAs, differential expression analysis identified a total of 2802 differentially expressed genes (DEGs) (|Log2(FC)| ≥ 1; adjusted *p*-values < 0.05), including 2066 up-regulated and 736 down-regulated genes (Figure 3A, Table 1, Supplementary Table S4). Regarding the small RNA sequence analysis, 180 differentially expressed small RNAs were identified, including 80 upregulated and 100 downregulated transcripts (Figure 3B, Table 2, Supplementary Table S5). Interestingly, the majority of up-regulated small RNAs consists of small nucleolar RNAs (snoRNAs), while almost all down-regulated small RNAs are miRNAs or piRNAs. 72% of DE mRNAs are up-regulated, instead 93% of DE lncRNAs are up-regulated.

### 3.3. Functional Enrichment Analysis

All DE mRNAs in EVs were processed to identify the enriched pathways to which they belong to infer the effects they produce in the cells that receive these EVs (Supplementary Table S6). In interpreting these data, it must be taken into account that, probably, the main target of these EVs is constituted by the adjacent tumor cells, whereas other EVs will reach different tissues. According to long RNA sequencing, among the enhanced pathways consisting of up-regulated genes, we find those related to the axon guidance and axonogenesis, ECM remodeling, cell adhesion, Epithelial to Mesenchymal Transition (EMT), lipid metabolism, and ABC-type xenobiotic transporters.

By performing enrichment analysis of genes found to be down-regulated in EVs, we can highlight the pathways that are no longer enhanced following eribulin treatment. In other words, in the recipient cells, these pathways were enhanced by the EVs released from the untreated cells, but, following treatment, the EVs no longer enhance these pathways. Among the no longer enhanced pathways, we have found protein synthesis, RNA transcription, RNA maturation, and energy production (glycolysis and mitochondrial respiration). Full results are reported in Supplementary Table S7.

### 3.4. Identification of Key Genes Associated with the Eribulin Treatment by WGCNA

**Figure 4:**
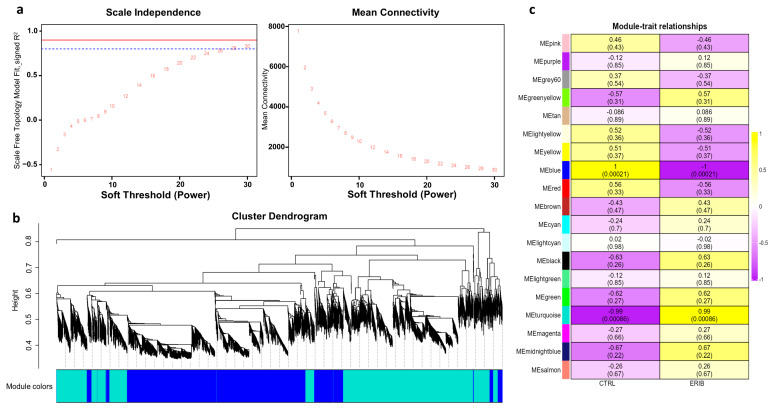
Results of weighted gene co-expression network analysis (WGCNA). (**a**) Identification of the optimal soft-threshold power by calculation of scale-free topology fit index and mean connectivity. In the left panel, R^2^ values of the Scale Free Topology Model Fit and the corresponding soft threshold powers are shown. The optimum power is 28 since it is the minimum value above the threshold set at 0.8 (dashed blue line). In the right panel, the mean connectivity as a function of the soft-threshold power is shown. These graphs confirm that the network we have constructed has a scale-free topology. (**b**) Clustering dendrograms and modules identified by WGCNA: each color represents a different module. (**c**) Module-to-trait analysis between the gene modules and eribulin treatment (ERIB) or controls (CTRL). For each module, the Person’s correlation coefficients and their *p*-values, within parenthesis, are provided. Only the statistically significant modules (i.e., turquoise and blue) were further investigated. Curiously, these two modules are also those with the largest number of genes. This was not expected since significance and coefficients do not depend on the module size.

We performed the weighted gene co-expression network analysis on our gene expression data, obtained by RNA sequencing from EVs released from MDA-MB-231 cells after eribulin treatment. WGCNA identifies similar expression profiles (co-expression) of genes among samples, and it groups highly co-expressed genes into network modules. Moreover, the most central and connected genes, called “hub” genes, well represent the functions of the entire module, and they may be involved in pathological processes or have important clinical implications as potential diagnostic and prognostic biomarkers or therapeutic targets [62]. We previously adopted WGCNA to study mRNA, miRNA and lncRNA expression in pancreatic cancer [63,64,65]. In this study, WGCNA has been applied in order to identify modules and their key genes associated with the eribulin treatment in breast cancer cells. Firstly, we constructed a gene co-expression network and verified whether it had a scale-free topology, as is required for WGCNA. The scale-free topology is the structure of all biological networks, and it consists in some nodes (i.e., hub genes) that are more connected than others (i.e., peripheral genes). Our network had the scale-free topology since the R^2^ scale-free topology fit index reached values above 0.8 at the power = 28 (Figure 4a). Since we obtained this low power, it implied that there were no batch effects in our data. Then, we performed hierarchical clustering and identified 19 gene co-expression modules consisting of a different number of genes and named with different colors according to WGCNA package functions (Figure 4b, Supplementary Table S8). Next, we set out to evaluate the correlations between the characteristics of the modules and the treatment conditions (eribulin or controls). Only two modules (turquoise and blue) were statistically significantly (*p* < 0.05) associated with the treatment (Figure 4c). In particular, the turquoise module was associated with the eribulin treatment (cor = 0.99; *p* = 0.00086), while the blue module was associated with the controls (cor = 1; *p* = 0.00021).

Among these two modules, the top 10 genes with the GS (Gene Significance, i.e., the correlation with the treatment status) scores are reported in Table 3. The functional enrichment analysis of these genes highlighted that the turquoise module (associated with the eribulin treatment) contains genes mainly involved in “Actin Filament-Based Transport”, “Extracellular Matrix Organization”, and “Crosslinking of Collagen Fibrils”. In these pathways, enhanced following eribulin treatment, the involved genes are *PXDN*, *MYO1G* and *CAPN11*, *DSE*. In the blue module (associated with the untreated cancer cells), the enriched pathways were “Cilium Disassembly”, “Microtubule”, “Tubulin Binding”, “Myosin Binding”, and “Lamellipodium Assembly”, where the genes *KIF1C*, *MAP4*, *ACTG1*, *ABLIM3*, *TRIOBP* are involved. WGCNA results indicated that eribulin induced a strong alteration in the EV content, mainly affecting genes encoding proteins involved in the organization of the cytoskeleton and extracellular matrix. In particular, pathways associated with the cytoskeleton were typical of EVs from untreated cancer cells, whereas pathways associated with the extracellular matrix were typical of EVs from eribulin-treated cells.

### 3.5. Experimentally Assessed Target Genes of miRNAs in EV

We aimed to identify genes involved in chromosomal instability (CIN), immune checkpoint, and drug resistance that are targeted by miRNAs contained in EVs released from MDA-MB-231 under normal conditions (here called down-regulated miRNAs) and after eribulin treatment (up-regulated). The results of the analysis performed with the mirTarbase tool (which contains only experimentally validated miRNA-mRNA interactions) show some genes involved in CIN, immune checkpoint, and drug resistance (Table 4).

### 3.6. Comparison of MicroRNA Expression in EVs and Cells

**Figure 5:**
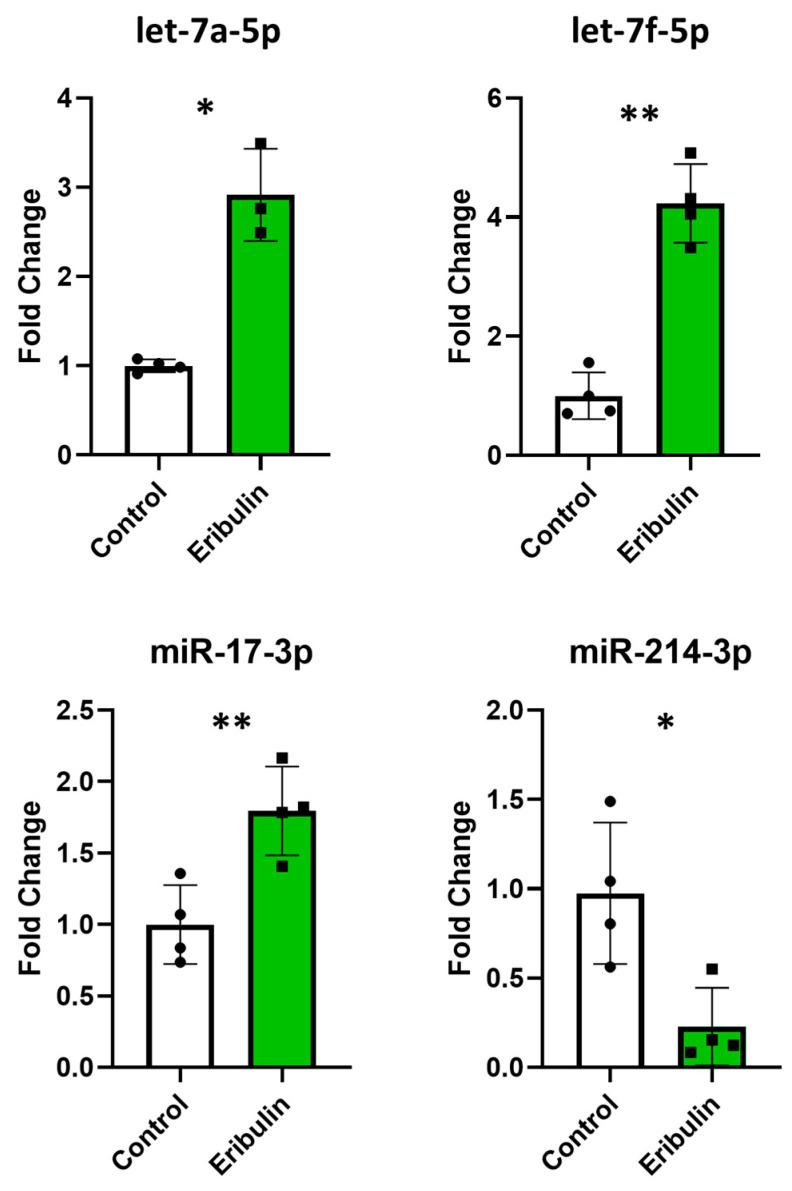
Quantitative RT-PCRs performed with the miRCURY LNA SYBR^®^ Green PCR Kit and miRCURY LNA miRNA PCR Assays (Qiagen) to assess the intracellular expression levels of *let-7a-5p*, *miR-17-3p*, *let-7f-5p* and *miR-214-3p*. The evaluations were performed in quadruplicate on control and eribulin-treated cells. * *p* < 0.05; ** *p* < 0.01.

We verified if eribulin influenced the cellular expression of microRNAs or their sorting into EVs. Therefore, we determined the intracellular levels of the most differentially expressed EV microRNAs. The three most overexpressed microRNAs in the EVs after treatment were *let-7a-5p*, *miR-17-3p*, and *let-7f-5p* with fold changes of about 1.9, 1.8 and 1.6, respectively, and fold changes of about 2.9, 1.8 and 4.2, respectively, in cells. Among the most down-regulated microRNAs (*miR-214-3p*, *miR-155-5p*, *miR-196-5p*), only miR-214-3p had a sufficient expression level to be detected. Notably, its expression level was decreased by about 4-fold in both cells and EVs (Figure 5). Thus, although not linearly for all microRNAs tested, changes in the expression levels of EV microRNAs mirror those of parent cells.

### 3.7. Association of Other Small RNAs with Breast Cancer

Finally, we searched for possible associations between breast cancer and piRNAs, snoRNAs and tRNA fragments, by using some prediction tools. iPiDi-PUL tool highlighted that 4/9 (44%) upregulated piRNAs and only 2/40 (5%) downregulated piRNAs are predicted to be associated with breast cancer (Supplementary Table S9). Since piRNAs associated with breast cancer were both up- and downregulated by the eribulin treatment, we cannot draw definitive conclusions. By using the RNADisease tool, we found one upregulated snoRNA (*U44/RNU44/SNORD44*) and one downregulated snoRNA (*U8/SNORD118*) in eribulin-treated EVs. Notably, in the literature, it was found that *SNORD44* is downregulated in breast cancer and associated with a poor prognosis, whereas *SNORD118* was upregulated in breast cancer and its depletion inhibited tumorigenicity of breast cancer cells in vivo and in vitro [66]. Since eribulin induced upregulation of *SNORD44* and downregulation of *SNORD118*, eribulin could exert its anti-tumor roles also by affecting the expression of these snoRNAs. According to tRFExplorer database, the tRNA fragments *tRFdb-5026a*, *tRFdb-5020a*, *tRFdb-3004a* and *ts-112* are more expressed in breast cancer than normal tissues. They are more expressed also in specific subtypes, such as LumA, LumB, Her2 and triple-negative (Supplementary Table S10). Our data show that eribulin treatment decreased the expression levels of *tRFdb-3004a* and *ts-112*, suggesting a positive role of eribulin, but it would not affect the levels of *tRFdb-5026a* and *tRFdb-5020a*.

## 4. Discussion

For the first time, RNA sequencing on long and small RNAs in EVs released from eribulin treated MDA-MB-231 triple-negative breast cancer cells have been carried out to obtain an overview of the communication through EVs between eribulin-treated and untreated cells. Among up-regulated genes, we identified many pathways that could be enhanced in the EV-recipient cells, including axon guidance and axonogenesis, ECM remodeling, adhesion, EMT, lipid metabolism, and ABC-pumps.

Regarding the axon guidance and axonogenesis-enriched pathways, which serve to attract or repel growing axons and migrating neurons in the developing nervous system, the presence of peripheral nerves in the microenvironment of epithelial carcinomas is associated with more aggressive disease [67] and higher is the grade of breast cancer and more numerous and thicker are the nerve fibers [68]. Since highly aggressive human TNBC tumors are enriched for genes associated with neurogenesis [69], if our observed enrichment of these pathways was induced in microenvironment cells, it could enhance the nerves and therefore cancer migration and metastasis. Moreover, the enhancement of these pathways could also occur in cancer cells, inducing migration and invasion [70]. However, among the genes enriched in the axon guidance pathway, some also have a tumor suppressor role [71], so we are not able to deduce the overall role of these enriched genes.

We also identified the ECM remodeling, a process useful to cancer to create a matrix that supports tumor growth and constitutes a physical barrier to evade immune surveillance by T-cells. Moreover, proteolytic ECM degradation generates bioactive compounds that promote tumor proliferation, migration, invasion and angiogenesis [72,73]. ECM proteins can also establish a physical and biochemical niche for cancer stem cells (CSCs) [74]. Consistently, our WGCNA results indicated that pathways associated with the extracellular matrix were typical of EVs from eribulin-treated cells.

Another pathway is related to cell adhesion, the increase of which restrict cell growth mainly through contact inhibition and limit tumor cell migration [75]. However, malignant cells can also utilize these pathways to promote tumor growth; in fact, the expression of various integrins promotes tumor cell proliferation, survival and metastases [76,77].

We identified up-regulated genes belonging to the Epithelial to Mesenchymal Transition (EMT) process, but since some of them promote epithelial and other mesenchymal status, we are not able to deduce whether, overall, they push towards one or another cell type. However, it was shown that eribulin can revert EMT [17], likely by inhibiting TGF-β-mediated Snail expression by impairing the microtubule-dependent nuclear localization of Smad2/3. Moreover, microtubule depolymerization mediated by eribulin induces c-Jun, which consequently increases Slug/*SNAI2* expression in cells with low Smad4 [78].

Another pathway is regarding lipid metabolism involved in cancer metabolic reprogramming to provide the additional requirements of energy and metabolites for cell proliferation and dissemination [79] and chemoresistance [80]. Therefore, lipid metabolic reprogramming is considered as a hallmark of cancer [81]. While most somatic cells obtain their lipids either from dietary sources or from lipids synthesized by the liver, various cancers reactivate de novo lipogenesis, making themselves more independent from externally provided lipids [82]. In breast cancer, de novo lipogenesis is enabled in the luminal subtype, whereas the use of exogenous fatty acids is typical in the basal-like receptor-negative subtypes [82].

Some up-regulated genes are ABC-type xenobiotic transporters that help cancer to achieve chemoresistance. These resistance mechanisms concern the multidrug resistance (MDR) system and are induced by various drugs, including eribulin [83]. Here, we assessed that *ABCB1* (P-glycoprotein) expression is strongly increased (log2FC = +5.301, about 40 times more) and it is known to be responsible for the eribulin resistance of many breast cancer cell lines, including the MDA-MB-231 used in this study [84,85,86]. In particular, eribulin induced an acquired resistance in breast cancer cells by inducing an overexpression of *ABCB1* and *ABCC11* genes [86]. Similarly, paclitaxel and docetaxel are also responsible for an increased expression of *ABCC2* and *ABCB1* genes in breast cancer [87,88]. In addition, docetaxel induced higher levels of *ABCG2* protein (also known as breast cancer resistance protein, BCRP) in EVs released from docetaxel-resistant MCF-7 cells [35]. Although in our EVs from eribulin-treated cancer cells, *ABCC11* mRNA was not up-regulated, we found that both *ABCC2* (log2FC = +4.570, about 23 times more) and *ABCG2* (log2FC = +2.366, about 5 times more) were up-regulated. However, it is not yet known whether eribulin is among their substrates.

Moreover, the WGCNA analysis highlighted the transporter *SLC22A5* (*OCTN2*) as key genes in the turquoise module associated with the eribulin treatment. Notably, *SLC22A5* is involved in the transport of many drugs, including the microtubule targeting agent (MTA) vincristine, vinblastine, vinorelbine, and paclitaxel [89]. Eribulin is also an MTA, but it is not yet known whether it can be transported by *SLC22A5.*

In addition to the well-known resistance mechanisms due to the expression of the multidrug resistance (MDR) system genes, further mechanisms of resistance to MTAs were identified. One is the activation of the PI3K/AKT survival pathway due to mutational activation of PIK3CA or inactivation of PTEN [90], so PI3K inhibition enhances the anti-tumor effect of eribulin in triple-negative breast cancer [91]. Subsequently, it was confirmed that mutations in *PIK3CA*, *PIK3R1* or *AKT1* activate PI3K pathway and confer resistance to eribulin [92]. Here, EVs from eribulin-breast cancer cells contained higher levels of *PIK3R5* (log2FC = +3.877), *PIK3C2B* (log2FC = +1.664) and *PIK3R1* (log2FC = +1.500) mRNAs than controls. Similarly, it is possible that these mRNAs transferred to the recipient cells induce chemoresistance.

In addition, c-Fos is upregulated following to eribulin treatment in the triple-negative breast cancer cell lines MDA-MB-231 and HCC70 and this was related to low eribulin sensitivity [93]. Here, both *FOS* (log2FC = +1.571) and the other Fos family member *FOSB* (log2FC = +1.770) are upregulated in EVs from eribulin-treated cells. This supports the possibility that eribulin induced stress could spread a chemoresistance message.

The genes in which RNA was found to have a down-regulation in EVs of treated cells show the pathways that were supported in recipient cells by the EVs of the untreated cells and are no longer supported by the EVs of the treated cells. These pathways involved the cell cycle (protein synthesis, RNA transcription, ATP synthesis), and therefore, if the recipient cells are tumor cells, its slowing down could be interpreted as a positive effect due to decreased proliferation. On the other hand, this could be a defense mechanism that would make cancer cells less prone to be affected by the MTA- and DNA-targeting drugs.

We also performed RNA sequencing on small RNAs in EVs released from eribulin-treated MDA-MB-231 triple-negative breast cancer cells. Our results showed that the expression levels of some small RNA transcripts are associated with both anti-tumor and pro-tumor effects.

Among sequenced small RNAs, there are the PIWI-interacting RNAs (piRNAs), a novel class of small non-coding RNAs (26–31nt) that form the piRNA silencing complex involved in transposon silencing, genome rearrangement, epigenetic regulation, and protein regulation. The functions of piRNAs in normal or pathological states are still largely unknown. Recently, it was demonstrated that piRNAs’ abnormal expression is associated with the progression of several cancer types, including breast cancer. There was also observed a role in the maintenance of cancer stemness and chemoresistance. Therefore, they could serve as novel biomarkers and therapeutic targets for tumor diagnostics and treatment [94,95,96]. Our analysis identified forty-nine differentially expressed piRNAs, nine of which are up-regulated and forty of which are down-regulated. An analysis of these piRNAs in the literature and in specific databases returned only *piR-hsa-7193*, which we found strongly down-regulated (log2(FC)= −4.4), and it is known to be highly up-regulated in breast cancer patients [97]. Since this piRNA is typical of cancer, its expression lowering could be a positive effect of eribulin.

The novel class of small RNAs named tRNA-derived fragments consists of tRNA precursors or fragments of mature tRNAs. These RNAs have recently drawn great attention due to the identification of further biological roles in tumorigenesis and cancer progression apoptosis and metastasis, including in breast cancer [98]. They play many regulatory roles, including gene silencing, RNA stability, and translation [98]. Recently, a role in drug resistance has also been proposed [99,100]. Among tRNA-derived fragments identified in our EVs, *ts-112* is already known to have oncogenic potential in breast cancer. Indeed, its inhibition in MCF10CA1a aggressive breast cancer cells reduced cell proliferation, whereas its over-expression in normal breast MCF10A cells increased proliferation [101]. Interestingly, in EVs released after eribulin treatment, *ts-112* is strongly down-regulated (log2(FC) = −4.8) so, similarly to *piR-hsa-7193*, this oncogenic tRNA-derived fragment is no longer transferred by EVs to the recipient cells, resulting in a positive effect of eribulin.

The small nucleolar RNAs (snoRNAs) are involved in the post-transcriptional modifications of other RNAs, and recent studies have suggested the snoRNAs as diagnostic or prognostic biomarkers. Krishnan et al., by sequencing small RNAs of normal and cancerous breast tissues, identified some snoRNAs associated with a patient’s overall survival [102]. Among them, they identified *SNORD46* as down-regulated in tumor tissues. On the contrary, in EVs from eribulin-treated cells, we found that *SNORD46* is up-regulated (log2(FC) = 2.3), suggesting an anticancer effect of eribulin.

Among over-expressed miRNAs in EVs derived from cells treated with eribulin, *let-7a-5p* is associated with bortezomib sensitivity according to a study performed in 34 different breast cancer cell lines (MD-MBA-231 included) [103]. Another overexpressed miRNA is *miR-30c*, which sensitizes MDA-MB-231 to paclitaxel and doxorubicin [104].

Among down-expressed miRNAs in EVs derived from cells treated with eribulin, miR-671-5p is known to inhibit epithelial-to-mesenchymal transition, induce S-phase arrest, and sensitize breast cancer cells to cisplatin, 5-fluorouracil (5-FU) and epirubicin [105]. Low expression of *miR-148a-3p* in TNBC is associated with the development of metastases [106]. Interestingly, EVs from MDA-MB-231 cells showed higher levels of *miR-155* than EVs from non-metastatic breast cancer cells (MCF7) and non-tumor cells (MCF10A) [107]. Down-regulation of *miR-155-5p* decreases the effectiveness of Olaparib [108] and enhances the anti-tumor effect of cetuximab in MDA-MB-231 cells [109]. *MiR-125b-5p* seems to have opposite effects; in fact, it confers resistance to paclitaxel-treated MDA-MB-231 cells [110], while it can also confer sensitivity to paclitaxel-resistant cells [111]. Low *miR-214* expression confers chemoresistance to tamoxifen and fulvestrant in metastatic MCF7 cells [112]. Finally, EV-contained *miR-122-5p* is transferred from breast cancer cells to non-tumor cells to suppress glucose uptake by silencing pyruvate kinase; this increases the nutrient availability for cancer cells, and metastasis is facilitated [113]. The diversity of effects of all these molecules does not allow us to draw a conclusion regarding the final effect of these EVs.

We identified the experimentally assessed target genes of miRNAs in EVs and evaluated their effects. Some microRNAs are already known to target important checkpoint proteins involved in the onset of CIN [114]. Chromosomal instability (CIN) is an increased frequency of changes in chromosome structure. It is considered a hallmark of cancer as it plays a role in tumorigenesis, cancer progression, and chemoresistance [55].

Among the genes we found silenced by upregulated miRNAs, *AURKA*, *KLF4*, and *MDM2* are known to regulate CIN. The overexpression of Aurora A kinase (*AURKA*) and the proto-oncogene *MDM2* induces CIN by centrosome amplification, cytokinesis failure and dysregulation of cell-cycle proteins. Instead, the knockout of *KLF4* (Kruppel-like factor 4) induces centrosome amplification and breakages [55]. Overall, the identified up-regulated miRNAs seem to counteract chromosomal instability.

Some down-regulated miRNAs (present in EVs before eribulin treatment) are already known to silence *APC*, *CTNNB1*, *FBXW7*, *ID1*, *TP53*. Thus, these genes that were silenced under normal conditions are no longer silenced after eribulin treatment. In particular, depletion or mutation of *APC*, β-catenin (*CTNNB1*), hCdc4 (*FBXW7*) and *TP53* are known to induce CIN by dysregulation of cell-cycle proteins, merotely and checkpoint defects. However, ID1 overexpression can induce cytokinesis failure [54]. Overall, the identified down-regulated miRNAs no longer seem to induce chromosomal instability in the EV-recipient cells. Interestingly, it should be noted that both p53 and its specific inhibitor Mdm2 are targets of the EV-contained miRNAs: the up-regulated *miR-29a-3p* silences Mdm2 and the down-regulated *miR-125a-5p* no longer silences p53 after eribulin treatment. This suggests an increase in p53 amount and therefore tumor suppressive effects.

Furthermore, three miRNAs (*miR-451a*, *mir-214-3p*, *miR-328-3p*) known to silence the multi-drug resistance proteins ABCB1 (MDR1, P-glycoprotein) and ABCG2 (BCRP, breast cancer resistance protein) were down-regulated in eribulin-treated EVs; *mir-214-3p* is also known to silence the immune checkpoint PD-L1. Since, following eribulin treatment, these miRNAs are no longer transferred to other cells via EVs, higher levels of MDR proteins and PD-L1 could be induced in tumor recipient cells, which could become more resistant to drugs and immune system.

Our comparison of the expression of some vesicular versus cellular microRNAs suggested that eribulin treatment does not affect sorting in vesicles, but the cellular content. If this were also true for all EV microRNAs, it would mean that the sampling of EVs from body fluids is representative of the content of tumor cells, confirming the validity of liquid biopsies.

## 5. Conclusions

Our results showed that EVs of eribulin-treated cancer cells contain mRNAs that could contribute to EMT, adhesion, ECM remodeling, and lipid metabolism. In addition, on the one hand, eribulin could exert a positive role by decreasing chromosomal instability, and on the other hand, it seems to also have a negative role because it could increase eribulin resistance and immune escape. However, we must also point out that according to some evidence, the microRNAs transferred by EVs could be in insufficient quantity to modify the functioning of the recipient cells [115]; moreover, other molecules carried by exosomes, as proteins and lipids, could also be responsible for an effect.

Moreover, we must be aware of the weaknesses of currently available methods, which do not allow us to reach high resolutions. In fact, the RNA sequencing shows only the average of many different messages carried by each single EV released by the cancer cells. Instead, each EV could carry a specific message and bring it to a specific target cell. A better decryption of the message carried by the EVs will be possible with the improvement of techniques; in particular, we refer to single-cell sequencing applied to exosomes [116].

## Figures and Tables

**Table 1:** The top 20 (best fold change) upregulated and downregulated mRNAs, lncRNAs are reported.

Gene Name	Category	log_2_ (Fold-Change)	*p* Adjusted
*AQP4-AS1*	lncRNA	7.59	2.71 × 10^−8^
*AC002463.3*	lncRNA	7.39	1.02 × 10^−7^
*GABRG3*	protein_coding	7.36	1.31 × 10^−7^
*CASS4*	protein_coding	7.18	4.00 × 10^−7^
*NEFM*	protein_coding	6.94	2.53 × 10^−7^
*MLIP*	protein_coding	6.87	4.40 × 10^−7^
*CALN1*	protein_coding	6.61	5.66 × 10^−8^
*FER1L5*	protein_coding	6.59	4.47 × 10^−8^
*OTOG*	protein_coding	6.55	7.29 × 10^−8^
*CELF2-AS2*	lncRNA	6.45	1.49 × 10^−7^
*ATP10B*	protein_coding	6.39	1.63 × 10^−9^
*RP11-799O21.2*	lncRNA	6.37	2.70 × 10^−7^
*FAM83F*	protein_coding	6.36	2.54 × 10^−7^
*TDRD12*	protein_coding	6.36	2.82 × 10^−7^
*RP4-715N11.2*	lncRNA	6.35	3.15 × 10^−7^
*POU2F3*	protein_coding	6.35	3.02 × 10^−7^
*GRM8*	protein_coding	6.34	3.00 × 10^−9^
*NID2*	protein_coding	6.33	3.84 × 10^−7^
*LINC02822*	lncRNA	6.33	4.40 × 10^−7^
*MMP16*	protein_coding	6.32	3.76 × 10^−7^
*H3C10*	protein_coding	−3.22	5.04 × 10^−21^
*ANP32B*	protein_coding	−3.22	5.72 × 10^−117^
*TP53INP2*	protein_coding	−3.24	1.82 × 10^−39^
*IGIP*	protein_coding	−3.30	5.91 × 10^−9^
*BCL2L2*	protein_coding	−3.33	1.19 × 10^−33^
*NUDT16*	protein_coding	−3.43	1.84 × 10^−55^
*SH3PXD2A*	protein_coding	−3.43	2.76 × 10^−96^
*CRTAP*	protein_coding	−3.44	9.71 × 10^−143^
*H4C11*	protein_coding	−3.49	2.83 × 10^−5^
*PGPEP1*	protein_coding	−3.54	7.71 × 10^−73^
*ZSWIM9*	protein_coding	−3.63	5.21 × 10^−47^
*C22orf46*	transcribed unitary pseudogene	−3.65	3.46 × 10^−167^
*CTD-3099C6.13*	unknown	−3.72	8.58 × 10^−120^
*FBXW4*	protein_coding	−3.74	2.42 × 10^−50^
*RAB13*	protein_coding	−3.89	2.47 × 10^−96^
*RASSF3*	protein_coding	−3.97	9.52 × 10^−168^
*KIF1C*	protein_coding	−4.05	0.00 × 10^−0^
*TRAK2*	protein_coding	−4.17	5.02 × 10^−249^
*NET1*	protein_coding	−4.43	0.00 × 10
*RP11-603J24.7*	processed_pseudogene	−4.46	3.44 × 10^−95^

**Table 2:** The top 20 (best fold change) upregulated and downregulated miRNAs, piRNAs, tRNA-derived-ncRNAs, snoRNAs are reported.

Gene Name	Category	log_2_ (FoldChange)	*p* Adjusted
*SNORD98*	snoRNA	4.03	1.95 × 10^−3^
*SNORA47*	snoRNA	3.55	2.34 × 10^−11^
*SNORD71*	snoRNA	3.25	2.83 × 10^−8^
*SNORD12C*	snoRNA	3.13	3.25 × 10^−3^
*SNORD28*	snoRNA	3.07	2.34 × 10^−11^
*piR-hsa-23566*	piRNA	3.01	1.54 × 10^−5^
*SNORD37*	snoRNA	2.87	4.50 × 10^−4^
*SNORD90*	snoRNA	2.72	4.61 × 10^−3^
*SNORD18B*	snoRNA	2.71	6.04 × 10^−3^
*SNORD127*	snoRNA	2.67	1.26 × 10^−2^
*SNORA62*	snoRNA	2.55	2.61 × 10^−10^
*SNORD19B*	snoRNA	2.48	2.94 × 10^−2^
*SNORD83A*	snoRNA	2.35	3.60 × 10^−8^
*piR-hsa-1834*	piRNA	2.34	1.15 × 10^−2^
*SNORD46*	snoRNA	2.33	1.39 × 10^−4^
*piR-hsa-26039*	piRNA	2.32	1.01 × 10^−5^
*SNORD34*	snoRNA	2.27	3.36 × 10^−2^
*SNORD78*	snoRNA	2.20	1.64 × 10^−7^
*SNORD123*	snoRNA	2.18	1.40 × 10^−4^
*SNORD49A*	snoRNA	2.15	1.54 × 10^−5^
*piR-hsa-27513*	piRNA	−4.53	7.26 × 10^−4^
*tRFdb-3004a-617*	tRNA-derived ncRNA	−4.73	4.84 × 10^−5^
*hsa-miR-155-5p*	miRNA	−4.82	7.75 × 10^−6^
*ts-112*	tRNA-derived ncRNA	−4.83	1.59 × 10^−5^
*piR-hsa-7116*	piRNA	−4.91	7.12 × 10^−6^
*piR-hsa-28382*	piRNA	−5.09	1.18 × 10^−4^
*piR-hsa-17793*	piRNA	−5.32	8.62 × 10^−4^
*piR-hsa-2467*	piRNA	−5.49	3.59 × 10^−5^
*piR-hsa-28205*	piRNA	−5.76	1.09 × 10^−4^
*5P_tRNA-His-GTG-1-8*	tRNA-derived ncRNA	−6.28	4.44 × 10^−5^
*piR-hsa-28478*	piRNA	−6.29	5.08 × 10^−4^
*piR-hsa−5939*	piRNA	−6.56	3.80 × 10^−7^
*piR-hsa-25046*	piRNA	−6.67	7.45 × 10^−8^
*ts-44*	tRNA-derived ncRNA	−7.33	1.02 × 10^−5^
*piR-hsa-12789*	piRNA	−7.42	1.48 × 10^−4^
*hsa-miR-6087*	miRNA	−7.99	2.68 × 10^−5^
*hsa-miR-1306-5p*	miRNA	−8.19	5.79 × 10^−16^
*hsa-miR-196b-5p*	miRNA	−8.45	2.07 × 10^−5^
*piR-hsa-9105*	piRNA	−9.15	1.64 × 10^−7^
*piR-hsa-12275*	piRNA	−10.76	2.15 × 10^−7^

**Table 3:** The top 10 hub genes with the best Gene Significance score in the turquoise and blue modules. The turquoise module is associated with the eribulin treatment, whereas the blue module with controls.

Gene Symbol	Gene Name	Module	GS Score	Functions *
*PXDN*	Peroxidasin	turquoise	0.9949	Peroxidase secreted into the extracellular matrix, contributes to the collagen IV network-dependent fibronectin/FN and laminin assembly, which is required for full extracellular matrix (ECM)-mediated signaling.
*PVT1*	Pvt1 oncogene	turquoise	0.9946	LncRNA oncogene, also in breast cancer. It also promotes extracellular matrix degradation (PMID: 35399100)
*WDR27*	WD repeat domain 27	turquoise	0.9944	Scaffolding for proteins
*SLC22A5*	Solute carrier family 22 member 5	turquoise	0.9944	Carnitine and polyspecific organic cation transporter, critical for elimination of many endogenous cations, toxins and drugs, including etoposide, oxaliplatin, imatinib, vincristine, vinblastine, paclitaxel, sunitinib, vinorelbine, cisplatin, oxaliplatin (PMID: 31861504)
*MYO1G*	Myosin IG	turquoise	0.9943	Unconventional myosin required during immune response
*LDLRAD4*	Low density lipoprotein receptor class A domain containing 4	turquoise	0.9941	Involved in negative regulation of cell migration; negative regulation of epithelial to mesenchymal transition. Functions as a negative regulator of TGF-beta signaling and thereby probably plays a role in cell proliferation, differentiation, apoptosis, motility, extracellular matrix production and immunosuppression.
*CAPN11*	Calpain 11	turquoise	0.9941	Calcium-dependent cysteine proteases of substrates involved in cytoskeletal remodeling and signal transduction
*PHIP*	Pleckstrin homology domain interacting protein	turquoise	0.9933	Regulates glucose transporter translocation
*KTN1-AS1*	KTN1 antisense RNA 1	turquoise	0.9929	LncRNA
*DSE*	Dermatan sulfate epimerase	turquoise	0.9929	Biosynthesis of the dermatan sulfate, a component of extracellular matrix (ECM)
*TRIOBP*	TRIO and F-actin binding protein	blue	0.9999	Associates with and stabilizes F-actin structures
*KIF1C*	Kinesin family member 1C	blue	0.9999	Microtubule-dependent molecular motor that transport organelles and move chromosomes during cell division
*NET1*	Neuroepithelial cell transforming 1	blue	0.9999	Guanine nucleotide exchange factor (GEF)
*MAP4*	Microtubule associated protein 4	blue	0.9999	Microtubule-associated protein, promotes microtubule assembly
*CDA*	Cytidine deaminase	blue	0.9999	Enzyme involved in pyrimidine salvaging
*ABLIM3*	Actin binding LIM protein family member 3	blue	0.9998	Interacts with actin filaments and is a component of adherens junctions
*CARD19*	Caspase recruitment domain family member 19	blue	0.9998	Mitochondrial protein
*ACTG1*	Actin gamma 1	blue	0.9998	Actin gamma
*TCEAL3*	Transcription elongation factor A like 3	blue	0.9998	Transcription elongation factor
*TOMM20*	Translocase of outer mitochondrial membrane 20	blue	0.9998	Mitochondrial import receptor

* Functions: as reported in NCBI Gene and Uniprot, if not specified.

**Table 4:** Validated targets of up- and down-regulated miRNAs in EVs isolated following eribulin treatment.

Up-Regulated miRNAs in EVs after Eribulin-Treatment	Validated Targets	Role *
*hsa-let-7a-5p*	*AURKA*	CIN
*hsa-miR-29a-3p*	*KLF4*	CIN
*hsa-miR-29a-3p*	*MDM2*	CIN
**Down-Regulated miRNAs in EVs after Eribulin-Treatment**	**Validated Targets**	**Role**
*hsa-miR-125a-5p*	*TP53*	CIN
*hsa-miR-144-3p*	*FBXW7*	CIN
*hsa-miR-155-5p*	*APC*	CIN
*hsa-miR-214-3p*	*CTNNB1*	CIN
*hsa-miR-214-3p*	*ABCB1 (MDR1, P-glycoprotein)*	MDR
*hsa-miR-214-3p*	*CD274 (PD-L1)*	IMMUNE
*hsa-miR-328-3p*	*ABCG2 (BCRP)*	MDR
*hsa-miR-381-3p*	*ID1*	CIN
*hsa-miR-451a*	*ABCB1 (MDR1, P-glycoprotein)*	MDR

* CIN: chromosomal instability; MDR: multidrug resistance; IMMUNE: immune checkpoint.

## Data Availability

The data presented in this study are available on request from the corresponding author.

## Supplementary Materials

The following supporting information can be downloaded at: https://www.mdpi.com/article/10.3390/cells13060479/s1, Figure S1: Evaluation of the RNA quality in EVs; Figure S2: Boxplots with normalized counts of long ncRNAs and mRNAs and small RNAs; Table S1: Quantity of the isolated EV RNAs; Table S2: Ranges of reads for each sample; Table S3: Total number of reads; Table S4: Full list of differentially expressed long RNAs; Table S5: Full list of differentially expressed small RNAs; Table S6: Enrichment Pathway analysis of up-regulated genes; Table S7: Enrichment Pathway analysis of down-regulated genes; Table S8: Modules identified in the network by WGCNA functions and their relative size; Table S9: iPiDi-PUL tool predictions; Table S10: tRNA fragment expression data from tRFtarget database.
