# Supplementary material for: Effects of Eribulin on the RNA Content of Extracellular Vesicles Released by Metastatic Breast Cancer Cells
Source: Cells. 2024 Mar 8;13(6):479. doi: 10.3390/cells13060479 (PMC10969587; doi:10.3390/cells13060479)
Supplement: Supplementary file 1 [file cells-13-00479-s001.zip › cells-2653028-supplementary.pdf]

SUPPLEMENTARY MATERIAL for:

Effects of Eribulin on the RNA Content of Extracellular Vesicles Released by Metastatic Breast Cancer Cells

by Giulietti M et al.

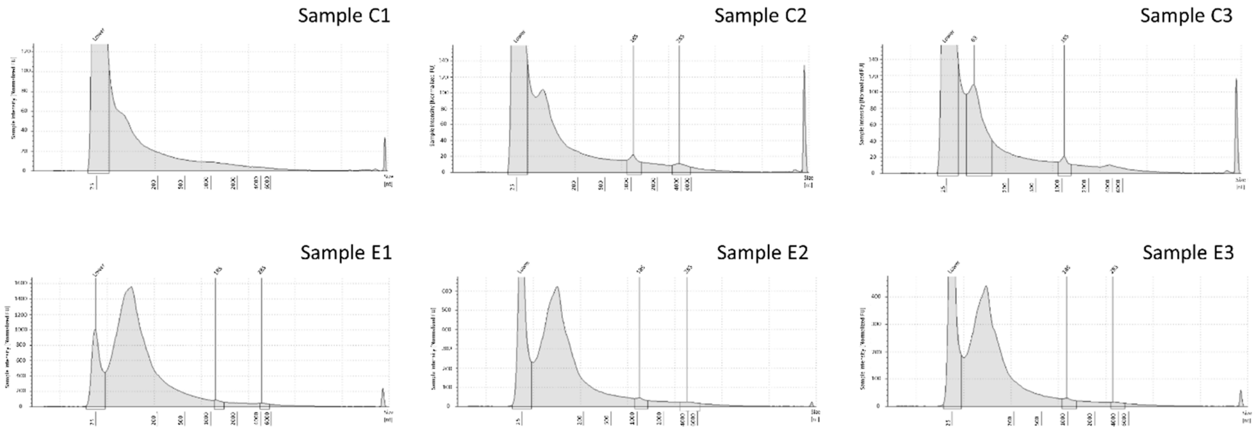

Supplementary Figure S1. Evaluation of the RNA quality in EVs from MDA-MB-231 (control samples: C1, C2, C3; eribulin-treatment: E1, E2, E3). Electrophoretic spectra of EV RNA obtained with RNA Nano 6000 Kit in an Agilent 2100 Bioanalyzer are shown. FU, fluorescence units; nt, nucleotides.

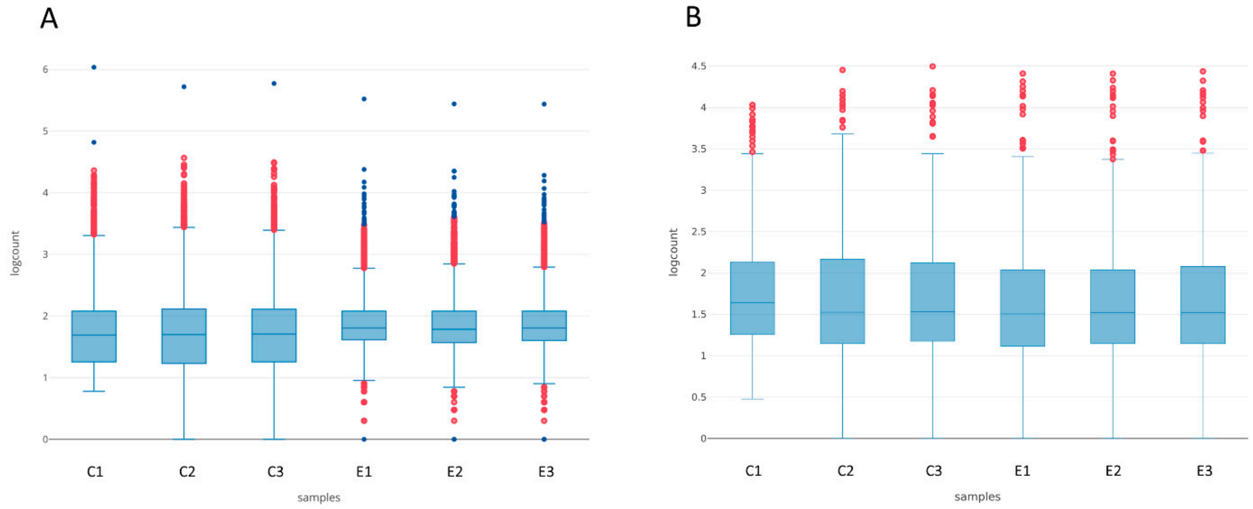

Supplementary Figure S2: Boxplots with normalized counts of long ncRNAs and mRNAs (A) and small RNAs (B). The count distribution and some statistics are represented in these plots. C1, C2, C3: biological replicates of EV RNAs from untreated cells. E1, E2, E3: biological replicates of EV RNAs from eribulin treated cells. Red and blue dots represent outliers.

Supplementary Table S1. Quantity of the isolated EV RNAs.

Sample name	Sample volume (µl)	Concentration (ng/µl)	Total quantity (ng)
C1	40	0.13	2.50
C2	40	0.22	4.36
C3	40	0.20	4.08
E1	40	2.10	42.00
E2	40	0.95	19.06
E3	40	0.75	14.94

Supplementary Table S2. Ranges of reads (minimum and maximum number of reads) for each sample. This information details the Supplementary Figure S2. Note that, as widely accepted we filtered out genes with an average number of read <10 across samples, therefore it is still possible to find genes with a minimum number of reads equal to 0 in some samples of this table.

RNA-seq type	Sample name	MIN number of reads, log10		MAX number of reads, log10	
Long RNA library	C1	4	0.6	1,158,511	6.06
	C2	0	n/a	626,945	5.80
	C3	0	n/a	681,428	5.83
	E1	0	n/a	286,185	5.46
	E2	0	n/a	244,751	5.39
	E3	0	n/a	236,733	5.37
Small RNA library	C1	3	0.48	10,974	4.04
	C2	0	n/a	28,631	4.46
	C3	0	n/a	31,518	4.50
	E1	0	n/a	25,483	4.41
	E2	0	n/a	25,018	4.40
	E3	0	n/a	27,166	4.43

Supplementary Table S3. Total number of reads (before and after normalization) in control and eribulin-treated groups, and in relation to the long and small libraries.

RNA-seq type	Sample type	Total number of reads (raw count)	Total number of reads (after normalization)
Long RNA library	Control	10,730,479	9,404,380
	Eribulin-treated	9,802,108	5,142,157
Small RNA library	Control	758,456	648,301
	Eribulin-treated	1,177,824	715,850

Supplementary Table S4. Full list of differentially expressed long RNAs (i.e. mainly mRNAs and lncRNAs). The table shows gene name, gene type, amount of differential expression in terms of logarithm of the base 2 of the Fold change (log2FC), adjusted P-value.

Name	Biotype	adjusted p-value	log_2(FoldChange)
AQP4-AS1	lncRNA	2.71E-08	7.593
AC002463.3	lncRNA	1.02E-07	7.390
GABRG3	protein_coding	1.31E-07	7.356
CASS4	protein_coding	4.00E-07	7.178
NEFM	protein_coding	2.53E-09	6.937
MLIP	protein_coding	4.40E-09	6.872
CALN1	protein_coding	5.66E-08	6.609
FER1L5	protein_coding	4.47E-08	6.591
OTOG	protein_coding	7.29E-08	6.545
CELF2-AS2	lncRNA	1.49E-07	6.446
ATP10B	protein_coding	1.63E-09	6.388
RP11-799O21.2	lncRNA	2.70E-07	6.373
FAM83F	protein_coding	2.54E-07	6.362
TDRD12	protein_coding	2.82E-07	6.357
RP4-715N11.2	lncRNA	3.15E-07	6.348
POU2F3	protein_coding	3.02E-07	6.347
GRM8	protein_coding	3.00E-09	6.340
NID2	protein_coding	3.84E-07	6.326
LINC02822	lncRNA	4.40E-07	6.325
MMP16	protein_coding	3.76E-07	6.321
FRMPD1	protein_coding	4.79E-07	6.297
AGBL4	protein_coding	1.57E-08	6.165
ITPRID1	protein_coding	2.08E-10	6.160
CNTN5	protein_coding	1.98E-10	6.137
CFAP74	protein_coding	3.74E-08	6.129
SLC30A10	protein_coding	3.87E-08	6.048
KSR2	protein_coding	1.93E-11	6.035
SLIT3	protein_coding	2.70E-11	6.021
RP11-622O11.6	lncRNA	8.46E-08	5.986
RP11-191L9.4	lncRNA	1.90E-14	5.985
RNA5-8SN3	rRNA	9.80E-09	5.970
JAML	protein_coding	2.38E-09	5.968
SOX5	protein_coding	1.25E-07	5.918
TCP10L3	transcribed_unprocessed_pseudogene	3.22E-07	5.886
RP11-400D2.2	lncRNA	9.50E-09	5.864
GOLGA6L22	protein_coding	9.12E-09	5.863
OPRD1	protein_coding	2.61E-07	5.861

LINC00687	lncRNA	3.49E-07	5.836
LINC02669	lncRNA	3.49E-07	5.827
LINC01902	lncRNA	1.22E-08	5.826
ACTN2	protein_coding	1.51E-10	5.823
FAM177B	protein_coding	4.55E-07	5.790
SCN1A	protein_coding	9.50E-09	5.786
SDK2	protein_coding	3.60E-11	5.760
GRIK3	protein_coding	7.77E-06	5.738
RP11-1396O13.23		9.70E-07	5.729
SLC22A23	protein_coding	2.09E-08	5.713
CSMD2	protein_coding	3.42E-19	5.712
GRIA4	protein_coding	2.11E-09	5.698
RP4-671O14.7		2.37E-09	5.680
LINC02397	lncRNA	2.70E-09	5.677
CNTNAP5	protein_coding	1.22E-09	5.663
LINC01122	lncRNA	1.90E-11	5.655
HNF4A	protein_coding	2.86E-11	5.651
MYO18B	protein_coding	4.26E-09	5.640
RGS22	protein_coding	1.14E-07	5.632
LARGE1	protein_coding	8.02E-08	5.625
RNA5-8SN1	rRNA	5.02E-09	5.616
LINC02208	lncRNA	2.26E-09	5.612
PALMD	protein_coding	6.98E-08	5.600
ATP13A4	protein_coding	2.70E-09	5.595
RP11-794P6.1		1.75E-07	5.562
ADCY1	protein_coding	2.08E-07	5.550
SLC26A8	protein_coding	2.36E-07	5.549
RUNX1T1	protein_coding	2.05E-11	5.543
RP11-161I6.2		5.50E-09	5.541
RP11-752L20.3		1.47E-08	5.532
RNF157	protein_coding	1.65E-06	5.527
KCNT1	protein_coding	2.03E-07	5.525
CAGE1	protein_coding	1.19E-07	5.525
SILC1	lncRNA	7.91E-12	5.520
MUC12	protein_coding	4.20E-24	5.518
MYOM1	protein_coding	1.03E-08	5.516
NTM	protein_coding	1.05E-08	5.485
RP11-369E15.3		3.03E-08	5.483
MACROH2A2	protein_coding	2.19E-09	5.483
MIR7515HG	lncRNA	4.02E-08	5.477
LINC00877	lncRNA	5.04E-08	5.450
KCNJ16	protein_coding	5.42E-07	5.443
RNA5S2	rRNA	8.12E-07	5.438
HEPHL1	protein_coding	9.89E-07	5.437
GRIN2A	protein_coding	3.56E-08	5.436
RP4-737E23.7		2.93E-08	5.424

RP1	protein_coding	1.85E-08	5.418
ASXL3	protein_coding	6.99E-08	5.417
BPIFB1	protein_coding	5.90E-08	5.410
LINC00971	lncRNA	2.12E-08	5.408
CTNND2	protein_coding	3.87E-08	5.402
SULF1	protein_coding	1.04E-07	5.381
ITIH2	protein_coding	7.77E-07	5.373
CSMD1	protein_coding	1.55E-10	5.335
ADD2	protein_coding	2.38E-08	5.326
ZAN	protein_coding	6.60E-09	5.321
LINC00598	lncRNA	0.0044332	5.313
ABCB1	protein_coding	2.08E-07	5.301
SOX2-OT	lncRNA	2.55E-17	5.281
AMPH	protein_coding	1.49E-05	5.272
DPP6	protein_coding	1.76E-10	5.268
PLCB1	protein_coding	2.29E-11	5.264
LINC01255	lncRNA	5.51E-08	5.260
TNN	protein_coding	4.55E-07	5.260
ADAMTSL3	protein_coding	1.27E-07	5.257
MXRA5	protein_coding	6.47E-10	5.252
DAAM2-AS1	lncRNA	8.02E-13	5.250
CLCN1	protein_coding	4.41E-07	5.242
RP11-81F8.1		5.60E-10	5.241
LINC01151	lncRNA	2.69E-09	5.239
DSCAML1	protein_coding	1.29E-08	5.235
AC097467.2		5.19E-08	5.232
C2orf16	protein_coding	1.49E-09	5.219
CDH26	protein_coding	2.82E-07	5.217
SSUH2	protein_coding	5.27E-07	5.214
NFASC	protein_coding	9.43E-14	5.212
RPH3A	protein_coding	5.30E-11	5.205
MROH9	protein_coding	2.65E-08	5.200
PHACTR3	protein_coding	1.22E-07	5.198
SORCS1	protein_coding	8.59E-08	5.197
COL6A5	protein_coding	6.70E-07	5.184
LINC00958	lncRNA	7.25E-07	5.182
NCAM1	protein_coding	3.75E-08	5.178
RP11-259O2.1		6.99E-08	5.174
CHGB	protein_coding	1.05E-08	5.170
MDGA2	protein_coding	3.33E-09	5.153
PCDH10	protein_coding	4.30E-07	5.148
SLC24A4	protein_coding	3.61E-10	5.146
IGF1	protein_coding	1.46E-07	5.145
MYH1	protein_coding	4.71E-08	5.144
ALOX12P2	transcribed_unprocessed_pseudogene	4.96E-07	5.143
AC079807.4		5.38E-08	5.142

ABCA12	protein_coding	3.89E-08	5.140
ZBTB16	protein_coding	1.55E-12	5.135
MYBPC3	protein_coding	1.38E-06	5.130
LINC01828	lncRNA	1.24E-06	5.128
FGFR2	protein_coding	1.15E-09	5.126
FAM153CP	lncRNA	2.56E-10	5.126
HMCN1	protein_coding	7.17E-13	5.124
MYBPC1	protein_coding	7.34E-08	5.119
RPTN	protein_coding	1.32E-06	5.117
SGK2	protein_coding	5.43E-07	5.114
A2ML1	protein_coding	1.90E-06	5.113
TFAP2B	protein_coding	8.92E-08	5.103
CACNA2D3	protein_coding	2.68E-07	5.102
ATP1A2	protein_coding	3.01E-07	5.089
LRRC6	protein_coding	2.35E-06	5.083
TMEM178B	protein_coding	7.24E-08	5.083
A2M	protein_coding	5.94E-08	5.076
EPHA7	protein_coding	6.97E-07	5.068
TCHHL1	protein_coding	2.36E-06	5.063
CDH6	protein_coding	2.01E-08	5.057
SORCS3	protein_coding	1.16E-06	5.051
CD101	protein_coding	1.20E-06	5.051
ERICH3-AS1	lncRNA	6.11E-07	5.044
RBFOX1	protein_coding	1.65E-07	5.036
STMND1	protein_coding	1.31E-05	5.035
RP11-491F9.1		1.80E-08	5.034
SYT9	protein_coding	3.24E-06	5.032
IL10RA	protein_coding	1.17E-08	5.032
KIF25	protein_coding	1.62E-06	5.030
WSCD2	protein_coding	6.67E-07	5.025
SSPOP	transcribed_unitary_pseudogene	2.45E-10	5.019
WDFY4	protein_coding	5.00E-13	5.018
LINC01622	lncRNA	6.99E-07	5.016
LINC01498	lncRNA	3.33E-07	5.012
LINC00343	lncRNA	6.39E-07	5.011
HYDIN2	transcribed_unprocessed_pseudogene	1.20E-13	5.002
DCDC2	protein_coding	3.33E-07	5.001
MACROD2	protein_coding	2.77E-07	4.995
LINC01287	lncRNA	2.25E-09	4.995
MARCHF1	protein_coding	2.65E-07	4.992
CNKSR2	protein_coding	9.82E-05	4.984
ENTPD1	protein_coding	5.69E-06	4.981
CTD-3088G3.8		9.32E-07	4.975
LINC01358	lncRNA	8.24E-09	4.971
DNAH10	protein_coding	1.03E-06	4.966
BNIP5	protein_coding	6.85E-08	4.964

SFMBT2	protein_coding	2.81E-06	4.961
IL12A-AS1	lncRNA	1.61E-06	4.953
KCNA2	protein_coding	3.40E-11	4.950
STEAP4	protein_coding	3.09E-07	4.948
LY9	protein_coding	1.40E-06	4.944
RP11-419I17.1		2.91E-07	4.942
RIPOR2	protein_coding	8.39E-09	4.936
ECM2	protein_coding	3.49E-07	4.933
AL591893.1		1.02E-07	4.929
SYCP2	protein_coding	3.76E-09	4.928
BASP1-AS1	lncRNA	5.71E-13	4.925
KIAA0319	protein_coding	6.36E-09	4.922
RP11-281A20.2		5.02E-09	4.919
GHR	protein_coding	9.81E-08	4.916
RP11-147G16.1		4.67E-08	4.898
ZNF667	protein_coding	3.71E-08	4.897
CFAP57	protein_coding	2.07E-06	4.896
C4orf50	protein_coding	3.18E-08	4.895
LSAMP	protein_coding	2.60E-06	4.890
LINC02702	lncRNA	3.21E-06	4.885
SNAP25-AS1	lncRNA	1.82E-07	4.885
MEF2C-AS1	lncRNA	2.02E-07	4.882
OPRM1	protein_coding	1.71E-09	4.876
RP11-373N22.3		2.97E-10	4.875
CTD-2314G24.2		3.00E-06	4.868
NID1	protein_coding	1.48E-06	4.868
DCN	protein_coding	5.24E-06	4.866
CTNNA3	protein_coding	5.95E-09	4.866
PRKN	protein_coding	6.62E-07	4.861
ACSM2B	protein_coding	5.61E-11	4.859
MEGF10	protein_coding	4.14E-06	4.857
UNC80	protein_coding	1.66E-09	4.855
LINC01206	lncRNA	1.06E-09	4.851
DGKG	protein_coding	2.10E-09	4.850
CNTNAP2	protein_coding	2.81E-08	4.847
LCT	protein_coding	4.68E-05	4.845
KC6	lncRNA	2.58E-07	4.843
IGSF22	protein_coding	6.45E-06	4.843
PTPRT	protein_coding	3.68E-09	4.842
LINC01814	lncRNA	3.94E-07	4.841
RGSL1	protein_coding	6.46E-09	4.828
OTOF	protein_coding	1.12E-06	4.828
NPM1P51	processed_pseudogene	4.66E-06	4.826
LINC02073	lncRNA	9.10E-07	4.822
ALK	protein_coding	1.81E-06	4.807
PAMR1	protein_coding	5.41E-06	4.799

LINC00923	lncRNA	1.51E-07	4.796
LINC02055	lncRNA	0.011642	4.790
RP3-468B3.2		3.51E-13	4.788
PNLIPRP1	protein_coding	2.68E-07	4.786
MUC16	protein_coding	0.0001249	4.783
ADGRF2	protein_coding	6.90E-06	4.781
MYT1L	protein_coding	0.0113118	4.767
SLC5A9	protein_coding	1.73E-06	4.764
AF064858.6		2.34E-06	4.756
GLB1L3	protein_coding	1.20E-05	4.749
TG	protein_coding	3.72E-10	4.749
MCF2L2	protein_coding	1.33E-11	4.746
GRIA2	protein_coding	2.77E-07	4.744
OR56A1	protein_coding	2.38E-07	4.742
COL19A1	protein_coding	0.0078832	4.734
SLA	protein_coding	2.81E-05	4.727
PRDM9	protein_coding	6.65E-09	4.726
VWA5B1	protein_coding	4.42E-06	4.724
COL6A6	protein_coding	3.60E-07	4.720
SPTBN4	protein_coding	8.24E-08	4.719
RP11-527D7.1		9.24E-06	4.718
STEAP1B	protein_coding	1.13E-07	4.711
STAB2	protein_coding	5.09E-08	4.701
GFRA1	protein_coding	5.83E-07	4.693
SLCO2B1	protein_coding	1.22E-08	4.692
RP11-1023L17.2		1.40E-10	4.691
LINC00534	lncRNA	6.78E-12	4.687
RP5-921G16.1		2.82E-11	4.686
L3MBTL1	protein_coding	3.87E-07	4.684
FGD5	protein_coding	2.12E-07	4.682
LRRC7	protein_coding	0.0074287	4.671
GADL1	protein_coding	5.12E-06	4.670
DSCAM	protein_coding	2.37E-07	4.669
TMPRSS4	protein_coding	2.68E-07	4.665
FLJ16779	lncRNA	2.13E-07	4.665
RP5-1120P11.3		1.96E-14	4.665
DAAM2	protein_coding	1.02E-17	4.663
NOL4	protein_coding	2.19E-06	4.662
NRAP	protein_coding	7.57E-07	4.661
FAM135B	protein_coding	3.80E-08	4.660
PKN2-AS1	lncRNA	6.26E-09	4.660
ENPEP	protein_coding	1.51E-08	4.654
LINC01811	lncRNA	3.13E-08	4.649
PCK1	protein_coding	2.86E-06	4.645
GUCY1A1	protein_coding	1.13E-06	4.642
CCDC26	lncRNA	4.10E-05	4.641

UNC5C	protein_coding	1.66E-07	4.641
LINC01934	lncRNA	1.86E-07	4.641
LDLRAD4	protein_coding	3.69E-15	4.641
RIMS2	protein_coding	3.80E-11	4.636
PTPRO	protein_coding	9.91E-07	4.631
MCF2L	protein_coding	5.82E-08	4.629
LINC02532	lncRNA	1.62E-06	4.628
PROX1-AS1	lncRNA	1.26E-08	4.627
LINC01630	lncRNA	2.70E-07	4.626
STYXL2	protein_coding	3.18E-05	4.621
LINC01435	lncRNA	4.99E-07	4.620
PCSK6	protein_coding	2.96E-06	4.620
PPP2R2B	protein_coding	4.80E-07	4.620
FER1L6	protein_coding	1.46E-08	4.617
SETBP1	protein_coding	3.56E-11	4.616
SLC6A1	protein_coding	8.59E-08	4.614
DNAH6	protein_coding	0.0037211	4.611
RP11-109I13.2		1.90E-06	4.608
LLNLF-96A1.1		4.12E-18	4.606
KCNB1	protein_coding	4.78E-12	4.602
SCN8A	protein_coding	1.04E-09	4.601
COL14A1	protein_coding	9.45E-09	4.601
DCHS2	protein_coding	8.65E-08	4.600
MAPK10	protein_coding	1.81E-15	4.600
ADGB	protein_coding	6.88E-05	4.599
LINC01837	lncRNA	4.22E-06	4.595
GPNMB	protein_coding	4.81E-06	4.594
RP11-90C4.1		1.29E-06	4.594
ANKS1B	protein_coding	2.61E-11	4.592
TRPC6	protein_coding	4.24E-06	4.591
KCNJ6	protein_coding	2.65E-08	4.590
MSC-AS1	lncRNA	9.00E-08	4.590
ANO1	protein_coding	2.10E-06	4.587
CASC15	lncRNA	0.0001361	4.586
WDR64	protein_coding	4.71E-07	4.584
VASH2	protein_coding	2.90E-06	4.581
FGD2	protein_coding	1.11E-09	4.581
ABCC8	protein_coding	1.03E-10	4.576
CADPS	protein_coding	0.007209	4.575
NTNG1	protein_coding	2.02E-06	4.572
ABCC2	protein_coding	8.14E-07	4.570
TMC1	protein_coding	1.08E-10	4.569
KCNC1	protein_coding	1.06E-07	4.567
TTLL9	protein_coding	3.03E-05	4.565
GRIN2B	protein_coding	1.04E-11	4.563
TECTA	protein_coding	9.89E-06	4.562

TNR	protein_coding	2.59E-07	4.558
CDH23	protein_coding	3.06E-09	4.558
PDZRN3	protein_coding	3.24E-06	4.556
CHRNA4	protein_coding	2.58E-06	4.554
PWRN1	lncRNA	0.0038042	4.551
GABRA2	protein_coding	7.77E-07	4.550
LINC01725	lncRNA	6.44E-06	4.549
DAB1	protein_coding	6.30E-06	4.547
RP11-115J16.1		1.77E-09	4.546
HMCN2	protein_coding	0.0084095	4.545
ABCA9	protein_coding	2.82E-07	4.545
RP11-203H2.1		5.76E-06	4.542
LINC02552	lncRNA	6.47E-07	4.541
EMX2OS	lncRNA	8.21E-06	4.539
TRPM3	protein_coding	1.81E-12	4.535
LGI1	protein_coding	3.71E-08	4.535
RP11-573D15.8		3.13E-07	4.530
GDA	protein_coding	3.70E-07	4.527
LRP1B	protein_coding	2.85E-10	4.525
RP11-436D23.1		2.00E-08	4.525
ELAVL4	protein_coding	3.80E-05	4.524
DLX6-AS1	lncRNA	1.51E-06	4.523
SRRM4	protein_coding	6.85E-06	4.521
RP11-159D12.12		8.61E-06	4.520
ABCB5	protein_coding	1.26E-06	4.518
ITIH5	protein_coding	1.31E-07	4.516
PTPRZ1	protein_coding	3.70E-07	4.514
WNT2B	protein_coding	9.57E-07	4.511
GPLD1	protein_coding	2.40E-07	4.511
PCAT1	lncRNA	2.17E-11	4.510
BMPER	protein_coding	2.65E-06	4.508
ITGA4	protein_coding	9.95E-06	4.506
CCDC162P	transcribed_unitary_pseudogene	4.62E-07	4.505
ADCY2	protein_coding	4.24E-07	4.502
CFAP47	protein_coding	8.16E-08	4.501
USP6	protein_coding	1.29E-06	4.500
THSD7A	protein_coding	9.55E-08	4.493
TMEM132B	protein_coding	2.14E-07	4.488
LINC00466	lncRNA	2.26E-05	4.480
ADGRB3	protein_coding	1.50E-07	4.475
CYP2E1	protein_coding	8.10E-10	4.473
DMP1	protein_coding	1.39E-05	4.468
SLC17A4	protein_coding	3.76E-08	4.468
TSHZ2	protein_coding	2.47E-09	4.453
LINC00907	lncRNA	5.43E-10	4.446
LNC-LBCS	lncRNA	1.36E-13	4.446

UNC13C	protein_coding	2.71E-06	4.445
TRDN-AS1	lncRNA	1.84E-05	4.445
TEX41	lncRNA	1.33E-11	4.441
LINC01036	lncRNA	1.19E-05	4.441
IQCA1L	protein_coding	1.91E-05	4.436
ABCA10	protein_coding	4.22E-06	4.435
THRB-AS1	lncRNA	7.77E-06	4.433
EYA2	protein_coding	1.14E-06	4.433
KCNN3	protein_coding	2.29E-08	4.430
CYP3A5	protein_coding	1.67E-05	4.429
TCF4	protein_coding	2.84E-12	4.426
AC013463.2		2.66E-08	4.421
BPI	protein_coding	2.54E-05	4.415
SCN11A	protein_coding	1.52E-06	4.412
RGS8	protein_coding	5.96E-08	4.408
REXO1L1P	transcribed_processed_pseudogene	6.57E-14	4.407
MAP3K19	protein_coding	8.47E-09	4.401
RP11-550I24.2		3.48E-05	4.400
RP11-15B24.5		3.24E-06	4.394
DOCK8	protein_coding	1.76E-09	4.393
ERICH3	protein_coding	1.49E-07	4.390
LINC02325	lncRNA	1.17E-05	4.389
ZNF667-AS1	lncRNA	1.27E-05	4.388
GRIP2	protein_coding	1.76E-06	4.387
KIAA2012	protein_coding	9.31E-07	4.387
GRM4	protein_coding	3.69E-07	4.385
STX18-AS1	lncRNA	1.47E-09	4.384
ADGRV1	protein_coding	1.99E-16	4.382
ABCC13	transcribed_unprocessed_pseudogene	8.02E-06	4.379
PAH	protein_coding	6.84E-06	4.378
RP11-822P4.2		4.66E-06	4.371
CSRNP3	protein_coding	8.31E-06	4.367
MYH7B	protein_coding	5.77E-06	4.366
SLC12A5	protein_coding	7.81E-06	4.364
DNAH8	protein_coding	0.0074799	4.360
ANO3	protein_coding	2.01E-06	4.360
ASTN1	protein_coding	7.70E-07	4.356
RP11-702N8.3		0.0001255	4.355
ATP8A2	protein_coding	1.94E-08	4.354
U3	snoRNA	2.36E-07	4.353
ABCA6	protein_coding	1.26E-07	4.353
RP11-429A20.3		2.22E-06	4.351
LINC01515	lncRNA	5.63E-07	4.348
CDRT1	protein_coding	1.75E-05	4.346
CORIN	protein_coding	5.32E-07	4.342
GABRG2	protein_coding	3.06E-09	4.340

LRP2	protein_coding	1.61E-10	4.338
MEG8	lncRNA	2.96E-06	4.338
AC079305.11		1.78E-05	4.330
COPG2IT1	lncRNA	3.48E-11	4.329
B3GAT1-DT	lncRNA	0.0001199	4.328
SLC44A3-AS1	transcribed_processed_pseudogene	1.75E-06	4.326
KIF1A	protein_coding	1.56E-06	4.323
DNAH7	protein_coding	1.44E-08	4.320
NCKAP1L	protein_coding	6.30E-06	4.318
KIF5A	protein_coding	2.13E-06	4.318
LINC02241	lncRNA	1.93E-07	4.317
MYH8	protein_coding	6.56E-06	4.316
ZNF793	protein_coding	0.0001533	4.313
GPRIN3	protein_coding	4.45E-06	4.313
RP11-381K20.2		1.39E-05	4.308
CNTN4	protein_coding	0.0001145	4.308
MYOCD	protein_coding	3.85E-06	4.306
SCN7A	protein_coding	1.60E-06	4.306
CACNB2	protein_coding	2.23E-06	4.305
MIR3976HG	lncRNA	0.006873	4.305
PHACTR1	protein_coding	2.36E-09	4.304
CFAP61	protein_coding	3.15E-05	4.300
NELL1	protein_coding	9.17E-06	4.288
CACNA1S	protein_coding	4.97E-06	4.278
GUCY1A2	protein_coding	1.90E-06	4.272
FLT1	protein_coding	4.47E-05	4.270
CHL1	protein_coding	1.16E-05	4.269
PTCHD4	protein_coding	1.29E-10	4.269
CHRM3	protein_coding	2.38E-07	4.267
CTCFL	protein_coding	3.73E-06	4.267
C3	protein_coding	0.0001713	4.263
LRRIQ1	protein_coding	2.42E-05	4.260
MASP1	protein_coding	3.73E-06	4.260
MROH2B	protein_coding	2.42E-05	4.254
MYO3A	protein_coding	2.93E-07	4.252
MYH4	protein_coding	2.93E-08	4.247
CXXC4-AS1	lncRNA	1.40E-05	4.247
IKZF3	protein_coding	5.89E-07	4.246
XXyac-YR38GF2.1		5.28E-06	4.240
CNTN2	protein_coding	1.61E-06	4.239
ATP13A5	protein_coding	8.19E-06	4.239
RP1-158P9.2		2.42E-05	4.236
AC006115.7		7.06E-08	4.231
ELN	protein_coding	3.93E-06	4.228
TENM1	protein_coding	5.31E-05	4.227
LINC01440	lncRNA	1.15E-05	4.221

LINC02607	lncRNA	3.28E-07	4.220
CACNA1G	protein_coding	5.29E-06	4.217
PCA3	lncRNA	9.43E-08	4.216
TENM2	protein_coding	5.20E-06	4.215
PKNOX2	protein_coding	1.13E-07	4.208
AC013460.1		2.58E-06	4.207
TDRD6	protein_coding	9.71E-06	4.206
ZNF385D	protein_coding	1.03E-07	4.206
LINC00461	lncRNA	3.64E-05	4.205
SLC8A1-AS1	lncRNA	9.02E-07	4.204
FCGR2A	protein_coding	3.79E-06	4.204
KY	protein_coding	7.44E-05	4.198
CACNA1D	protein_coding	1.69E-09	4.197
PLXNB1	protein_coding	1.11E-05	4.196
FYB1	protein_coding	4.96E-08	4.195
C6	protein_coding	8.83E-07	4.194
PCDH9	protein_coding	3.03E-05	4.192
CRX	protein_coding	7.81E-06	4.191
FMN2	protein_coding	6.46E-09	4.187
NOS1	protein_coding	0.0222009	4.186
LAMA4	protein_coding	6.17E-06	4.185
AP3B2	protein_coding	9.75E-06	4.185
MYH13	protein_coding	5.97E-07	4.179
F5	protein_coding	0.0226237	4.176
ANKRD11P1	processed_pseudogene	3.42E-06	4.175
RP11-111A21.1		1.07E-05	4.171
SPAG17	protein_coding	2.34E-08	4.169
SCN3A	protein_coding	2.07E-05	4.167
LINC00494	lncRNA	2.07E-05	4.163
AKAP6	protein_coding	1.28E-07	4.163
NKAIN3	protein_coding	3.32E-09	4.160
A1CF	protein_coding	1.03E-05	4.159
SNHG14	lncRNA	2.71E-13	4.158
LAMA1	protein_coding	2.85E-08	4.157
OTOGL	protein_coding	5.44E-08	4.156
ATP1A4	protein_coding	7.53E-08	4.155
TRIM9	protein_coding	4.84E-06	4.152
PKD1L2	polymorphic_pseudogene	2.04E-06	4.150
DPP4	protein_coding	2.13E-08	4.148
RPGRIP1	protein_coding	7.35E-05	4.147
CCDC168	protein_coding	5.90E-08	4.143
CNTN6	protein_coding	4.47E-05	4.140
VIM2P		1.17E-09	4.138
CMYA5	protein_coding	6.26E-08	4.137
COL22A1	protein_coding	2.87E-09	4.137
MIR99AHG	lncRNA	1.48E-07	4.135

MGAM	protein_coding	7.79E-05	4.134
LINC01597	lncRNA	2.53E-06	4.133
DPP10-AS1	lncRNA	1.18E-05	4.131
LINC00342	lncRNA	2.07E-06	4.131
CALML3-AS1	lncRNA	5.41E-06	4.130
SOX6	protein_coding	1.30E-06	4.129
SYN3	protein_coding	2.61E-05	4.129
LINC01016	lncRNA	1.15E-05	4.127
INHBA	protein_coding	7.37E-07	4.127
RHPN2P1	unprocessed_pseudogene	2.99E-05	4.126
PAPPA2	protein_coding	2.85E-07	4.124
SEMA3E	protein_coding	6.17E-07	4.122
SYT14	protein_coding	1.46E-05	4.118
BAGE2	transcribed_unprocessed_pseudogene	4.93E-06	4.118
LINC01344	lncRNA	3.89E-05	4.112
LINC00649	lncRNA	1.51E-05	4.112
SLC14A2	protein_coding	0.0001616	4.111
SAMMSON	lncRNA	1.12E-08	4.108
TSIX	lncRNA	2.34E-08	4.108
SEL1L3	protein_coding	3.29E-07	4.102
TRHDE-AS1	lncRNA	9.11E-06	4.098
HTR4	protein_coding	4.98E-05	4.096
TREM1	protein_coding	4.23E-05	4.096
RP11-329N11.1		6.07E-05	4.095
RAB44	protein_coding	1.76E-05	4.093
ANKRD31	protein_coding	2.35E-05	4.093
COL5A3	protein_coding	9.05E-07	4.092
MAGI2	protein_coding	1.03E-10	4.092
FRMPD4	protein_coding	5.64E-06	4.088
PALM3	protein_coding	2.34E-05	4.084
STAG3	protein_coding	3.96E-05	4.083
LINC00578	lncRNA	6.15E-05	4.081
RP3-470L22.2		3.25E-05	4.078
CADM1	protein_coding	7.51E-07	4.066
OPN5	protein_coding	1.27E-05	4.062
EYS	protein_coding	5.14E-08	4.059
RP3-394A18.1		1.72E-08	4.058
LYPLAL1-DT	lncRNA	9.66E-05	4.054
LINC00499	lncRNA	0.0130633	4.054
CACNA1E	protein_coding	2.44E-08	4.050
RETREG1	protein_coding	2.96E-06	4.050
ITGAL	protein_coding	5.29E-05	4.047
CPB2-AS1	lncRNA	3.38E-05	4.046
CDH12	protein_coding	3.83E-05	4.042
IGSF9B	protein_coding	6.30E-11	4.039
KCNK12	protein_coding	4.49E-05	4.039

ANK3	protein_coding	6.69E-14	4.038
FGF10	protein_coding	1.66E-07	4.038
EGFLAM	protein_coding	2.46E-05	4.037
RP4-681L3.3		9.64E-05	4.035
PDE3A	protein_coding	9.50E-05	4.032
FLJ40194	lncRNA	1.97E-05	4.029
CH507-513H4.3		5.44E-07	4.029
PDE11A	protein_coding	5.63E-10	4.029
TRIM66	protein_coding	2.44E-07	4.027
KLHL3	protein_coding	2.37E-06	4.027
GDNF-AS1	lncRNA	1.18E-06	4.025
ABCA4	protein_coding	1.43E-06	4.024
GOLGA6L2	protein_coding	3.56E-08	4.024
GABRA4	protein_coding	0.000248	4.020
LINC02465	lncRNA	2.03E-06	4.019
RP11-168K9.1		0.014045	4.018
ATP8B4	protein_coding	3.67E-05	4.016
AC011288.2		3.49E-07	4.015
PGBD5	protein_coding	0.0001237	4.014
ARHGAP28	protein_coding	4.15E-10	4.014
ITGA9	protein_coding	2.23E-05	4.009
MAP2K6	protein_coding	6.20E-06	4.007
CACNA1F	protein_coding	7.57E-07	4.004
RP11-427E2.1		4.04E-05	4.003
L3MBTL4	protein_coding	3.15E-05	4.001
GABRB2	protein_coding	5.45E-05	4.000
KCNJ15	protein_coding	2.42E-05	3.998
TRPV3	protein_coding	2.61E-05	3.997
PRRX1	protein_coding	3.03E-06	3.994
COL9A1	protein_coding	4.32E-07	3.994
LINC01088	lncRNA	8.61E-06	3.985
FAT2	protein_coding	2.60E-07	3.982
THBS2	protein_coding	0.0001371	3.978
RP1-223B1.1		4.62E-13	3.978
LINC01020	lncRNA	9.55E-05	3.976
ROBO2	protein_coding	4.50E-08	3.974
METAP2P1	processed_pseudogene	8.24E-06	3.973
MIR29B2CHG	lncRNA	3.70E-07	3.968
SPTB	protein_coding	2.82E-07	3.965
ATP7B	protein_coding	5.19E-06	3.963
CTD-2516F10.2		2.11E-06	3.961
PDE3B	protein_coding	3.69E-05	3.953
HULC	lncRNA	4.26E-05	3.952
RP3-329A5.8		5.95E-05	3.951
CYP46A1	protein_coding	2.78E-06	3.951
RP11-510D4.1		0.0001168	3.950

RAPGEF5	protein_coding	2.82E-05	3.948
SLC4A4	protein_coding	4.86E-07	3.945
SLC13A3	protein_coding	1.75E-05	3.945
MALRD1	protein_coding	2.06E-05	3.944
GTF2IP13	transcribed_unprocessed_pseudogene	0.0002821	3.944
SLC8A1	protein_coding	2.43E-07	3.943
AC010091.1		7.60E-06	3.942
LINC00299	lncRNA	0.0166884	3.942
ABCA8	protein_coding	4.63E-05	3.940
MYO7A	protein_coding	9.78E-07	3.938
DNAH17	protein_coding	4.65E-10	3.937
OFCC1	transcribed_unitary_pseudogene	1.78E-07	3.934
CD84	protein_coding	0.0002274	3.933
EIF1B-AS1	lncRNA	5.22E-07	3.932
IGFN1	protein_coding	1.25E-05	3.932
PCDH15	protein_coding	1.11E-06	3.930
CSMD3	protein_coding	3.72E-08	3.930
FAT3	protein_coding	2.68E-11	3.928
BTN2A3P	transcribed_unprocessed_pseudogene	4.30E-06	3.922
FAM189A2	protein_coding	4.00E-05	3.920
SVEP1	protein_coding	3.77E-06	3.918
PRKCQ-AS1	lncRNA	3.31E-05	3.915
LINC02008	lncRNA	0.0001347	3.914
KCNQ3	protein_coding	5.83E-07	3.914
LRP4	protein_coding	2.52E-08	3.910
ADAMTS4	protein_coding	2.30E-05	3.909
SCUBE3	protein_coding	1.16E-08	3.906
DGKK	protein_coding	3.59E-05	3.901
ACSM2A	protein_coding	7.90E-06	3.900
C1S	protein_coding	5.01E-05	3.899
AP000997.2		5.36E-05	3.895
RASGRF2	protein_coding	4.00E-05	3.893
ATP2B2	protein_coding	4.06E-07	3.888
PTPRN2	protein_coding	0.0002103	3.888
MYRIP	protein_coding	0.0001217	3.886
SI	protein_coding	2.43E-05	3.885
PEG3	protein_coding	2.35E-05	3.884
AC008697.1		1.10E-07	3.882
TLE4	protein_coding	0.0004015	3.881
PIK3R5	protein_coding	4.68E-05	3.877
CCDC39	protein_coding	5.29E-06	3.874
SLC7A14-AS1	lncRNA	0.0001414	3.874
RAB11FIP4	protein_coding	0.0002463	3.874
VWF	protein_coding	0.001168	3.873
MYLK4	protein_coding	1.16E-05	3.865
RYR3	protein_coding	1.35E-09	3.863

DGKB	protein_coding	0.0001051	3.861
NPHS1	protein_coding	0.0002531	3.859
AC074391.1		1.14E-06	3.857
COL24A1	protein_coding	3.57E-07	3.852
DISC2		2.04E-06	3.851
RP11-680H20.2		0.0001948	3.847
ST6GAL1	protein_coding	7.77E-07	3.846
FLG2	protein_coding	7.20E-05	3.843
SLC1A2	protein_coding	3.51E-07	3.836
NAALADL2	protein_coding	1.20E-06	3.836
TFAP2D	protein_coding	0.0003039	3.835
FREM2	protein_coding	0.0001132	3.835
CTB-95D12.1		0.0001328	3.830
XIRP2	protein_coding	9.17E-06	3.828
LMCD1-AS1	lncRNA	2.21E-05	3.827
POU6F2	protein_coding	9.01E-07	3.826
RELN	protein_coding	4.39E-09	3.824
C7	protein_coding	6.97E-06	3.824
GRIA1	protein_coding	5.17E-05	3.821
TMC3-AS1	lncRNA	3.69E-05	3.820
DMD	protein_coding	0.0045866	3.812
COL5A2	protein_coding	6.93E-06	3.811
XXbac-BPG55C20.7		1.14E-05	3.811
TTC41P	transcribed_unitary_pseudogene	1.14E-05	3.808
IMPG2	protein_coding	0.0001661	3.807
LINC01276	lncRNA	5.42E-05	3.806
CCSER1	protein_coding	8.63E-06	3.805
TF	protein_coding	1.12E-08	3.805
SPTA1	protein_coding	0.0109679	3.800
RP11-677M14.7		8.17E-05	3.796
APOB	protein_coding	3.89E-05	3.795
MYCBPAP	protein_coding	1.21E-05	3.791
GABRB3	protein_coding	0.0001033	3.790
LINC00486	lncRNA	2.94E-06	3.789
KCNH5	protein_coding	0.0002424	3.784
BMPR1B	protein_coding	1.21E-05	3.781
CASC19	lncRNA	1.46E-08	3.778
LINC02731	lncRNA	0.0001872	3.778
DMBT1L1	transcribed_unitary_pseudogene	4.08E-05	3.778
WDR17	protein_coding	3.50E-06	3.771
LAMTOR5-AS1	lncRNA	1.88E-07	3.769
KALRN	protein_coding	2.35E-13	3.769
ANO4	protein_coding	0.0003576	3.768
PEX5L	protein_coding	3.95E-05	3.767
RYR2	protein_coding	1.31E-09	3.764
ST8SIA1	protein_coding	4.74E-05	3.763

LINC00320	lncRNA	2.97E-05	3.755
PREX2	protein_coding	0.0001681	3.753
MPPED2	protein_coding	9.94E-05	3.753
DELEC1	lncRNA	0.0001308	3.744
DTHD1	protein_coding	5.11E-05	3.743
DDX43	protein_coding	0.0001217	3.739
LINC01204	lncRNA	0.000111	3.737
NEFMP1	processed_pseudogene	4.93E-05	3.737
TCHH	protein_coding	3.99E-10	3.732
RIMS1	protein_coding	2.62E-10	3.731
SRPX2	protein_coding	4.23E-05	3.727
LINC01551	lncRNA	0.0006476	3.725
AMZ1	protein_coding	4.68E-05	3.721
MYH2	protein_coding	8.24E-06	3.720
SIPA1L2	protein_coding	3.96E-06	3.714
SORBS1	protein_coding	6.54E-06	3.714
AJ006998.2		0.0001991	3.713
MEG3	lncRNA	2.93E-07	3.709
LINC02217	lncRNA	0.0002745	3.709
TTC21A	protein_coding	4.56E-06	3.703
PRKG2	protein_coding	1.48E-05	3.703
DRAIC	lncRNA	9.31E-09	3.696
RP11-281P23.3		0.0002789	3.691
SEC31B	protein_coding	2.91E-05	3.687
TMTC1	protein_coding	6.14E-05	3.683
RP11-421L21.3		0.0001132	3.680
HYDIN	protein_coding	8.84E-10	3.679
CD300E	protein_coding	2.21E-05	3.676
CCDC181	protein_coding	0.0001135	3.676
CACNB4	protein_coding	9.69E-09	3.669
PDGFRA	protein_coding	9.20E-05	3.667
DNAH3	protein_coding	4.17E-07	3.665
TTN-AS1	lncRNA	3.59E-12	3.665
ARHGEF33	protein_coding	0.0003994	3.664
NKD1	protein_coding	0.0002247	3.664
AGBL1	protein_coding	0.0001684	3.661
AC009264.1		0.0004601	3.657
RORC	protein_coding	8.41E-05	3.656
CTA-243E7.1		7.98E-05	3.655
LINC02884	lncRNA	5.97E-05	3.654
EML5	protein_coding	6.04E-06	3.650
FAM184B	protein_coding	0.0001246	3.646
LINC01847	lncRNA	0.0010686	3.644
LINC00870	lncRNA	0.0001371	3.643
TMEM67	protein_coding	1.03E-05	3.640
MGAT4C	protein_coding	3.83E-05	3.638

CDH2	protein_coding	8.85E-06	3.637
PDE6A	protein_coding	0.0004751	3.636
LHFPL5	protein_coding	4.22E-06	3.634
PTGFRN	protein_coding	0.0010547	3.632
EML6	protein_coding	5.11E-09	3.629
WT1	protein_coding	0.0002665	3.627
ADCY10	protein_coding	0.0014775	3.624
PLA2R1	protein_coding	1.16E-05	3.622
DLGAP1	protein_coding	0.0001168	3.620
KIRREL3	protein_coding	0.0001001	3.617
LINC01146	lncRNA	0.0001558	3.614
LINC00635	lncRNA	0.0024646	3.612
RORA	protein_coding	0.0005228	3.609
MBOAT1	protein_coding	1.58E-05	3.607
ZDHHC13	protein_coding	1.73E-05	3.605
NEB	protein_coding	1.22E-11	3.602
IKZF1	protein_coding	0.000187	3.598
LINC01320	lncRNA	9.79E-06	3.597
SOX9-AS1	lncRNA	2.33E-06	3.588
CTNNA2	protein_coding	5.31E-05	3.586
MCTP2	protein_coding	1.14E-06	3.584
AC006460.2		5.53E-05	3.578
FRAS1	protein_coding	9.50E-09	3.577
CLIC5	protein_coding	1.67E-05	3.575
ABCA13	protein_coding	4.45E-05	3.575
KIAA0825	protein_coding	0.000366	3.572
PRLR	protein_coding	4.11E-05	3.570
CTTNBP2	protein_coding	0.0001379	3.570
MYO15A	protein_coding	3.83E-06	3.564
ABI3BP	protein_coding	1.25E-06	3.564
ANGPT1	protein_coding	6.16E-05	3.563
PHEX	protein_coding	7.69E-06	3.559
ST3GAL6-AS1	lncRNA	2.54E-05	3.558
CMAHP	transcribed_unitary_pseudogene	4.68E-07	3.558
ELMO1	protein_coding	7.99E-06	3.556
MAP7D2	protein_coding	0.0003134	3.554
TRAF3IP3	protein_coding	5.41E-05	3.553
PAX6	protein_coding	7.03E-05	3.552
MOBP	protein_coding	0.0009973	3.551
CFAP43	protein_coding	1.34E-06	3.551
KIAA1549L	protein_coding	0.0001602	3.548
XACT	lncRNA	0.0003478	3.545
RP5-1014D13.2		4.13E-06	3.544
ACSM3	protein_coding	0.0001505	3.543
ENPP2	protein_coding	0.0002212	3.538
ADGRE2	protein_coding	2.01E-06	3.537

PDE4B	protein_coding	3.82E-05	3.536
NR2F2-AS1	lncRNA	2.21E-05	3.533
CHAT	protein_coding	0.0006796	3.531
HAND2-AS1	lncRNA	0.000735	3.529
ARHGAP20	protein_coding	8.36E-05	3.527
RP5-912I13.2		8.06E-05	3.521
SYNPO2	protein_coding	1.89E-05	3.516
MAP1A	protein_coding	7.47E-05	3.515
RP11-197K6.1		0.0001437	3.513
OSMR-AS1	lncRNA	9.08E-05	3.513
PECAM1	protein_coding	3.16E-05	3.513
SEMA6A	protein_coding	0.0003457	3.511
AC009518.4		7.02E-05	3.510
GALNT14	protein_coding	0.0003305	3.508
RALGPS1	protein_coding	9.77E-06	3.507
DOCK3	protein_coding	2.29E-07	3.507
ADGRL3	protein_coding	7.54E-05	3.499
CDHR3	protein_coding	3.09E-06	3.496
LINC00607	lncRNA	6.31E-05	3.493
LINC01524	lncRNA	7.21E-05	3.487
NWD1	protein_coding	8.24E-06	3.484
CTD-2553C6.1		0.0001991	3.481
LINC00861	lncRNA	0.0001251	3.481
SCN3B	protein_coding	0.0007423	3.479
MYH14	protein_coding	3.55E-05	3.479
CFTR	protein_coding	2.33E-05	3.477
RP1-206D15.6		0.0001775	3.476
RP11-115D19.1		0.0001069	3.464
NSRP1P1	processed_pseudogene	0.0017205	3.463
KCNQ1OT1	lncRNA	6.63E-27	3.459
LINC01192	lncRNA	0.0003612	3.456
RBM20	protein_coding	4.10E-06	3.455
DLEU1	lncRNA	1.16E-07	3.451
NTRK2	protein_coding	0.0003338	3.445
GABBR2	protein_coding	0.0001292	3.444
ADGRF4	protein_coding	0.0003912	3.441
SNORD3A	snoRNA	3.12E-52	3.436
ANKFN1	protein_coding	6.43E-06	3.434
RP1-258E1.2		1.26E-05	3.430
ZRANB2-AS2	lncRNA	4.65E-06	3.430
MIR3681HG	lncRNA	8.35E-06	3.428
CDH18	protein_coding	0.0001231	3.424
ADAM12	protein_coding	5.43E-06	3.419
SLC6A15	protein_coding	0.0002413	3.417
VCAN	protein_coding	0.0001474	3.417
GARNL3	protein_coding	1.43E-07	3.416

BCAS1	protein_coding	2.04E-06	3.414
SPEF2	protein_coding	5.59E-11	3.409
SULT1C2	protein_coding	1.32E-05	3.407
RP11-19F9.2		0.0001391	3.399
TEX14	protein_coding	7.08E-06	3.394
ALDH1A2	protein_coding	0.0001068	3.393
FSTL4	protein_coding	0.0004406	3.392
DTNA	protein_coding	8.60E-06	3.392
ZNF483	protein_coding	0.0006907	3.381
LINC01284	lncRNA	9.97E-05	3.379
USH2A	protein_coding	0.0193161	3.378
JAKMIP3	protein_coding	0.000459	3.377
NR4A3	protein_coding	5.51E-05	3.377
XKR4	protein_coding	6.33E-05	3.377
ZNF891	protein_coding	2.55E-05	3.373
RAD51B	protein_coding	3.05E-05	3.371
EPB41L4A	protein_coding	1.94E-06	3.369
ESRRG	protein_coding	3.97E-05	3.368
EGF	protein_coding	0.0001347	3.367
MPP4	protein_coding	0.0002211	3.366
PSG4	protein_coding	3.50E-05	3.364
RP11-154D6.1		0.0017557	3.362
COL11A1	protein_coding	0.0005037	3.359
CTD-2353F22.1		0.000339	3.359
TTBK1	protein_coding	0.0004483	3.355
IQGAP2	protein_coding	8.71E-06	3.355
SGIP1	protein_coding	0.0001084	3.353
L1TD1	protein_coding	0.0018914	3.353
FHAD1	protein_coding	7.72E-10	3.353
WDR87	protein_coding	7.49E-09	3.351
RP1-140K8.5		7.35E-05	3.350
KYNU	protein_coding	9.72E-08	3.346
PSMD7-DT	lncRNA	0.0030457	3.346
CRELD1	protein_coding	0.0002103	3.344
GRM5	protein_coding	6.11E-05	3.339
STRADA	protein_coding	0.0002704	3.339
SAP30L-AS1	lncRNA	0.0001719	3.335
GNG2	protein_coding	0.0001373	3.329
DMKN	protein_coding	4.50E-05	3.329
FER1L4	transcribed_unitary_pseudogene	3.59E-05	3.329
NRK	protein_coding	0.0009213	3.328
MEIS1	protein_coding	0.0010448	3.327
ST3GAL3	protein_coding	8.24E-06	3.324
LINC01197	lncRNA	0.0001461	3.324
SHROOM4	protein_coding	0.0001505	3.322
ZBED3-AS1	lncRNA	9.86E-05	3.320

ITPR1	protein_coding	1.14E-17	3.317
DNAH11	protein_coding	7.54E-12	3.312
RP13-143G15.4		0.0004759	3.308
FCN1	protein_coding	3.24E-05	3.308
RAB3B	protein_coding	2.52E-05	3.306
RNF217-AS1	lncRNA	0.0003555	3.305
PAXBP1-AS1	lncRNA	0.0003747	3.303
LINC00470	lncRNA	0.0028026	3.302
GDAP1	protein_coding	5.29E-06	3.302
ST8SIA5	protein_coding	0.0007775	3.301
STOX2	protein_coding	0.0001099	3.298
TRPM6	protein_coding	0.0002108	3.295
MUC19	protein_coding	1.86E-05	3.294
ARPP21	protein_coding	0.0004438	3.294
PRKG1	protein_coding	4.40E-06	3.291
LINC00271	lncRNA	0.0001474	3.286
RNF150	protein_coding	9.61E-05	3.283
CACNG8	protein_coding	4.23E-05	3.281
HELLPAR	lncRNA	0.001143	3.280
COL21A1	protein_coding	4.79E-05	3.279
ABCB4	protein_coding	0.0003032	3.263
LINC01138	lncRNA	0.0001281	3.261
PPP5D1	transcribed_unprocessed_pseudogene	0.000287	3.260
RALY-AS1	lncRNA	5.47E-05	3.256
PPFIA3	protein_coding	0.0002274	3.250
ITGA1	protein_coding	2.31E-08	3.249
SYBU	protein_coding	2.96E-05	3.246
ERBB3	protein_coding	1.46E-05	3.245
MIR646HG	lncRNA	6.58E-10	3.244
BCL11A	protein_coding	0.0002107	3.243
DAPK1	protein_coding	0.0014565	3.242
COL15A1	protein_coding	2.53E-05	3.241
LINC01226	lncRNA	0.0036952	3.233
TTN	protein_coding	0.0285051	3.219
HDAC2-AS2	lncRNA	1.02E-05	3.218
CASC11	lncRNA	0.0033661	3.217
NALCN	protein_coding	1.75E-06	3.211
NEK10	protein_coding	7.38E-05	3.211
ANK2	protein_coding	2.29E-18	3.210
TPRG1	protein_coding	0.0001836	3.204
TTC39A	protein_coding	0.0006039	3.202
OR2F1	protein_coding	0.0029118	3.199
ANKRD36BP2	transcribed_unprocessed_pseudogene	0.0020621	3.193
PLA2G4C	protein_coding	0.0025293	3.191
CNTNAP3	protein_coding	9.34E-06	3.190
CLCN5	protein_coding	1.52E-05	3.188

ERICH6B	protein_coding	0.0013404	3.185
RP11-33A14.1		0.0033471	3.184
TTC13	protein_coding	4.16E-05	3.183
AC092071.1		0.0001712	3.180
DNAH9	protein_coding	0.029103	3.177
PLD5	protein_coding	0.0033218	3.177
SNCAIP	protein_coding	0.0023539	3.176
TMEM232	protein_coding	0.0011079	3.172
PROM1	protein_coding	0.0064977	3.172
DNAH12	protein_coding	6.40E-05	3.169
CHD5	protein_coding	0.0011222	3.168
FRY	protein_coding	5.93E-05	3.165
DIXDC1	protein_coding	0.0001133	3.165
BAALC-AS1	lncRNA	1.69E-05	3.160
THSD7B	protein_coding	0.0008306	3.159
TMEM144	protein_coding	0.000304	3.158
PDZD2	protein_coding	3.35E-08	3.151
RP11-673E1.1		5.33E-05	3.147
SLC1A3	protein_coding	2.08E-08	3.145
PTPRB	protein_coding	0.0012744	3.144
HECW1	protein_coding	4.32E-07	3.144
ST18	protein_coding	0.0045307	3.144
DCC	protein_coding	0.006972	3.139
CEP43	protein_coding	4.76E-07	3.130
LINC01609	lncRNA	0.0007921	3.129
DSC2	protein_coding	0.0010593	3.124
ST3GAL5	protein_coding	2.35E-10	3.120
AXDND1	protein_coding	0.0002613	3.118
MIR4500HG	lncRNA	4.21E-05	3.117
LIFR-AS1	lncRNA	0.0009973	3.115
CGN	protein_coding	0.0002813	3.114
LINC01748	lncRNA	0.0013921	3.113
PLCB4	protein_coding	0.0012417	3.108
GAS7	protein_coding	0.0001606	3.107
RUBCNL	protein_coding	0.0089779	3.107
MUC5AC	protein_coding	7.98E-05	3.106
DGKE	protein_coding	0.0004055	3.101
CFAP54	protein_coding	0.0004262	3.101
CCDC187	protein_coding	0.0016229	3.096
BSN	protein_coding	0.0002876	3.094
SLC38A9	protein_coding	0.0001025	3.092
NRCAM	protein_coding	0.0011477	3.091
CCDC144NL-AS1	lncRNA	3.05E-05	3.089
RTCA-AS1	lncRNA	0.0003138	3.089
ADAM22	protein_coding	0.0003035	3.087
CPNE5	protein_coding	0.0004478	3.081

RASGEF1B	protein_coding	0.0018037	3.077
CEP63	protein_coding	3.24E-05	3.074
AFF3	protein_coding	1.50E-05	3.072
NUP210	protein_coding	5.16E-05	3.071
ZNF767P	transcribed_unprocessed_pseudogene	7.66E-05	3.069
CTD-2201E18.3		0.0002113	3.068
LINC01572	lncRNA	0.0001389	3.066
CNBD2	protein_coding	0.0004508	3.063
PPP1R9A	protein_coding	0.0003994	3.061
RP11-190A12.10		0.0002066	3.057
RP11-6G22.1		3.65E-05	3.057
ZFPM2	protein_coding	0.0035996	3.056
POT1-AS1	lncRNA	8.30E-07	3.052
LINC02223	lncRNA	0.0004363	3.052
CEACAM1	protein_coding	0.0013532	3.048
CPS1	protein_coding	0.0001588	3.044
RYR1	protein_coding	0.0443739	3.041
PKHD1L1	protein_coding	0.0104776	3.036
CDKL5	protein_coding	2.09E-05	3.036
CNTNAP1	protein_coding	6.73E-05	3.034
PPM1F-AS1	lncRNA	1.05E-05	3.033
SCART1	protein_coding	0.000974	3.032
LOXHD1	protein_coding	0.0102922	3.032
RP11-10K16.1		0.0004677	3.031
CFAP70	protein_coding	5.93E-05	3.028
Y_RNA	misc_RNA	0.0005442	3.026
MUC5B	protein_coding	0.0040694	3.023
DISC1	protein_coding	0.0005472	3.023
CYP1B1-AS1	lncRNA	3.00E-06	3.021
RP11-37B2.1		9.23E-07	3.020
ARHGAP30	protein_coding	0.0007078	3.019
VWA3B	protein_coding	0.0056907	3.018
PRKCH	protein_coding	0.000456	3.017
CCDC144CP	transcribed_processed_pseudogene	0.0023696	3.005
LINC01234	lncRNA	0.0009866	3.005
GBP5	protein_coding	0.0034504	3.003
GPR26	protein_coding	0.0014366	3.001
GOLGA2P5	transcribed_unprocessed_pseudogene	5.29E-05	2.999
SCIN	protein_coding	0.0007831	2.999
MYH15	protein_coding	9.73E-08	2.995
PXYLP1	protein_coding	0.000994	2.992
PAPPA	protein_coding	0.0006854	2.991
PLXNA4	protein_coding	1.67E-06	2.988
PTPRH	protein_coding	0.0003657	2.986
PRNCR1	lncRNA	0.0002292	2.985
PLCL1	protein_coding	0.0003077	2.978

OLMALINC	lncRNA	2.42E-05	2.977
ARMH4	protein_coding	0.0011679	2.976
EFCAB13	protein_coding	0.0001643	2.971
ZBTB7C	protein_coding	0.0029788	2.960
KLF8	protein_coding	0.0008626	2.959
NPL	protein_coding	0.0012103	2.952
PPFIA2	protein_coding	0.001616	2.944
PPARGC1B	protein_coding	0.0001005	2.943
SUSD4	protein_coding	0.0005939	2.940
NHSL2	protein_coding	0.0018274	2.939
NUTM2B-AS1	lncRNA	0.0017238	2.938
CD36	protein_coding	0.0060385	2.932
NRXN1	protein_coding	0.0304219	2.929
TGFBR3	protein_coding	3.46E-06	2.923
EPM2A	protein_coding	0.0003913	2.922
CELSR1	protein_coding	4.87E-05	2.922
ARMCX4	protein_coding	0.0028991	2.916
RP11-646I6.3		0.0039043	2.912
AGPAT4	protein_coding	0.0012514	2.911
MAILR	lncRNA	0.0002005	2.909
HSPA12A	protein_coding	2.82E-07	2.908
ADAMTS9-AS2	lncRNA	0.0033217	2.907
ABCA5	protein_coding	5.97E-05	2.906
COL1A1	protein_coding	0.0001263	2.906
SYP	protein_coding	0.0034012	2.901
CECR2	protein_coding	0.0125638	2.900
RP11-696N14.1		3.08E-05	2.899
MSI2	protein_coding	5.43E-05	2.897
KCNMA1	protein_coding	0.0399945	2.892
ADAMTS16	protein_coding	6.24E-05	2.892
PDE1C	protein_coding	7.81E-06	2.891
DLEC1	protein_coding	0.0035893	2.890
CH507-528H12.1		2.45E-08	2.888
C10orf90	protein_coding	0.0038439	2.880
TMEM41A	protein_coding	3.77E-05	2.875
MED12L	protein_coding	0.0004296	2.872
KIAA1755	protein_coding	0.0069561	2.872
ZFHX2	protein_coding	0.0001096	2.872
PREX1	protein_coding	0.0021879	2.869
ANKRD44	protein_coding	0.0001739	2.865
TNFSF15	protein_coding	0.0017436	2.862
PCDH7	protein_coding	0.0004795	2.861
SH3YL1	protein_coding	0.0010966	2.857
ACOXL	protein_coding	0.0010849	2.857
EPHA1	protein_coding	0.0016524	2.854
ADTRP	protein_coding	0.0003258	2.852

RP11-319G6.1		0.0007773	2.841
PLEKHH1	protein_coding	0.0001793	2.840
COBL	protein_coding	0.0001257	2.839
SRRM5	protein_coding	3.47E-05	2.836
IFI44L	protein_coding	0.000113	2.832
MYO16	protein_coding	0.0025315	2.816
OTULINL	protein_coding	3.66E-05	2.810
KLHL6	protein_coding	0.0050206	2.806
THRB	protein_coding	0.0042412	2.798
ABCA3	protein_coding	0.0024975	2.795
DOCK10	protein_coding	0.0002001	2.787
DGKI	protein_coding	3.57E-07	2.786
ADGRF1	protein_coding	0.0004409	2.785
PRTG	protein_coding	0.0001593	2.782
IRAG1	protein_coding	0.0100194	2.781
PTPRQ	protein_coding	0.002525	2.777
PDGFRB	protein_coding	0.0006444	2.770
SP140	protein_coding	2.13E-05	2.768
NCAM2	protein_coding	0.0009178	2.765
ADGRD1	protein_coding	0.0005839	2.757
EFHC1	protein_coding	8.41E-11	2.750
ERN1	protein_coding	1.26E-07	2.748
ELAPOR1	protein_coding	0.0039847	2.745
OBSCN	protein_coding	2.87E-06	2.744
DPF3	protein_coding	0.0012352	2.743
DEPDC5	protein_coding	0.0005655	2.742
ITGB8	protein_coding	0.000564	2.738
NOS3	protein_coding	0.0011715	2.736
FRMD4B	protein_coding	1.52E-05	2.732
CC2D2B	protein_coding	0.009779	2.729
ROBO1	protein_coding	0.0001885	2.729
MEGF6	protein_coding	0.0005063	2.729
TENM3	protein_coding	0.0006745	2.723
SMARCE1	protein_coding	0.0005931	2.720
RP11-823P9.3		0.010005	2.719
GALNTL6	protein_coding	0.008829	2.717
SLC4A8	protein_coding	1.34E-05	2.716
FILIP1	protein_coding	1.86E-05	2.712
SCN9A	protein_coding	0.0001385	2.711
RP11-295P9.3		0.0004431	2.706
LINC02615	lncRNA	0.0005011	2.701
RP1-23O21.2		0.0005559	2.700
AL163953.3		0.0002361	2.692
PPFIA4	protein_coding	0.0002538	2.691
PLEKHG4B	protein_coding	0.0001519	2.690
KIF21B	protein_coding	0.000255	2.688

MYRF	protein_coding	0.002624	2.687
ATL1	protein_coding	9.16E-07	2.686
CUBN	protein_coding	4.46E-05	2.679
COL16A1	protein_coding	0.0026357	2.675
XDH	protein_coding	0.000908	2.674
PCLO	protein_coding	2.68E-10	2.669
ABCC9	protein_coding	0.0012764	2.667
LINC00654	lncRNA	0.0143441	2.665
PTN	protein_coding	0.0026173	2.660
FAM9A	protein_coding	0.0074647	2.644
MAGI2-AS3	lncRNA	0.0003664	2.643
GVQW3	protein_coding	0.0002438	2.637
MED17	protein_coding	7.49E-05	2.635
SLC47A1	protein_coding	0.0034868	2.633
GREB1	protein_coding	0.0115278	2.630
LINC01588	lncRNA	1.21E-07	2.628
IRF1-AS1	lncRNA	0.0021845	2.621
LINC00856	lncRNA	0.0122761	2.620
EVC2	protein_coding	0.0029788	2.619
RECK	protein_coding	0.0039773	2.618
ASAH2	protein_coding	0.0029837	2.613
SLC13A4	protein_coding	0.0329267	2.596
TUB	protein_coding	0.0078319	2.596
DENND2C	protein_coding	0.0016669	2.595
POC1B-AS1	lncRNA	0.0006575	2.582
NOD1	protein_coding	0.0037865	2.579
C2CD6	protein_coding	0.0033547	2.579
DNAI4	protein_coding	0.0027915	2.578
C3orf35	lncRNA	0.0026441	2.578
DCLK1	protein_coding	0.0033453	2.570
MAP3K15	protein_coding	0.0020762	2.568
COL4A4	protein_coding	0.0058104	2.565
PLXNC1	protein_coding	0.0046844	2.564
FTX	lncRNA	1.29E-05	2.564
MYH3	protein_coding	2.24E-08	2.561
EYA4	protein_coding	0.0048122	2.556
TRIM52	protein_coding	0.0038646	2.549
MAST1	protein_coding	0.0022593	2.547
DNMT3A	protein_coding	1.29E-05	2.537
FSIP2	protein_coding	0.0005725	2.529
NR6A1	protein_coding	0.0003801	2.525
BTN2A2	protein_coding	7.60E-05	2.524
WDR27	protein_coding	0.0001158	2.519
GS1-519E5.1		1.61E-06	2.519
RGS5	protein_coding	0.0003913	2.511
RAPGEF3	protein_coding	0.0002915	2.509

NLGN1	protein_coding	0.0004592	2.507
RP11-712B9.2		0.0036866	2.505
ADAMTS1	protein_coding	0.0012417	2.502
GALC	protein_coding	0.0019452	2.500
PABPC1L	protein_coding	2.05E-05	2.500
NPR3	protein_coding	0.0051475	2.499
LINC01229	lncRNA	0.0077629	2.499
DNAH5	protein_coding	2.41E-08	2.499
DENND2A	protein_coding	0.0010558	2.498
ZNF737	protein_coding	0.0061271	2.498
TNS1	protein_coding	0.0014745	2.495
SLC35E3	protein_coding	2.35E-05	2.488
ZKSCAN4	protein_coding	0.0036787	2.486
SLC38A6	protein_coding	0.0002788	2.484
SYT12	protein_coding	0.0074357	2.483
SGSH	protein_coding	0.0055742	2.483
C5	protein_coding	5.08E-05	2.480
MARK1	protein_coding	0.0001398	2.476
FGGY	protein_coding	0.0003457	2.476
CFAP44	protein_coding	0.005846	2.474
PRDM16	protein_coding	0.0076606	2.464
TVP23C	protein_coding	0.0076304	2.462
LINC00472	lncRNA	6.99E-06	2.452
LMBRD1	protein_coding	0.0011716	2.447
CLHC1	protein_coding	0.0061879	2.445
CCDC142	protein_coding	0.0061703	2.444
NPHP1	protein_coding	2.97E-05	2.439
SORCS2	protein_coding	0.0042237	2.438
NEO1	protein_coding	0.0032643	2.437
RP11-242D8.1		0.0013046	2.437
ABAT	protein_coding	0.0041637	2.436
ATP9A	protein_coding	0.0001283	2.436
CCDC40	protein_coding	0.0005242	2.435
PDLIM3	protein_coding	0.0020292	2.434
ZNF141	protein_coding	0.0024922	2.426
ADAMTSL1	protein_coding	0.000304	2.424
LONRF2	protein_coding	0.0033751	2.424
ADCK1	protein_coding	0.0102283	2.421
ANKRD36C	protein_coding	3.70E-07	2.418
ANKRD36	protein_coding	4.32E-07	2.418
RP11-463O12.5		0.0035182	2.414
QRICH2	protein_coding	0.0011492	2.414
TMEM30A-DT	lncRNA	0.0022954	2.408
NRP2	protein_coding	3.55E-05	2.408
GREB1L	protein_coding	2.02E-05	2.403
NDUFA9	protein_coding	0.0124764	2.397

GDPD5	protein_coding	0.0034352	2.395
SLIT2	protein_coding	2.92E-06	2.392
TMEM132A	protein_coding	0.0020687	2.392
ATP8A1	protein_coding	0.00453	2.390
LRGUK	protein_coding	0.0201793	2.388
ATOH8	protein_coding	0.0193018	2.384
CYP2U1-AS1	lncRNA	0.0025611	2.379
RNASET2	protein_coding	7.06E-05	2.378
SEMA4D	protein_coding	2.09E-05	2.376
GRIK2	protein_coding	0.0018839	2.375
CSF1R	protein_coding	0.00422	2.374
MCM3AP-AS1	lncRNA	0.0018228	2.374
PDE2A	protein_coding	0.005876	2.373
PGAP1	protein_coding	0.006378	2.371
TRABD2A	protein_coding	0.0054519	2.368
ABCG2	protein_coding	0.0113238	2.366
CACNB3	protein_coding	0.0209144	2.365
ADAMTS9	protein_coding	0.0003129	2.360
TBCK	protein_coding	8.69E-05	2.359
WDPCP	protein_coding	0.0013364	2.358
FASTKD1	protein_coding	0.0067528	2.352
SUSD6	protein_coding	0.0043105	2.349
DZIP1L	protein_coding	0.0030827	2.347
INTS6-AS1	lncRNA	0.0451496	2.339
SYT1	protein_coding	0.0011466	2.339
COX10-AS1	lncRNA	0.0052992	2.338
BANK1	protein_coding	0.0016648	2.338
ACER3	protein_coding	2.42E-05	2.334
RIMKLA	protein_coding	0.0094705	2.333
CFH	protein_coding	0.0001469	2.328
CCDC150	protein_coding	3.79E-05	2.326
LRIG1	protein_coding	0.0043232	2.325
SORL1	protein_coding	5.47E-08	2.324
ARHGAP24	protein_coding	0.0025079	2.320
UBA6-AS1	lncRNA	0.0001199	2.317
CYFIP2	protein_coding	1.20E-05	2.314
IMMP2L	protein_coding	0.0139784	2.311
PAG1	protein_coding	0.0003242	2.310
CNKSR3	protein_coding	0.0003317	2.306
PTK7	protein_coding	0.0001391	2.305
ZC2HC1C	protein_coding	0.0008497	2.305
MYO5B	protein_coding	0.0036684	2.305
C1RL-AS1	lncRNA	0.0133867	2.304
WDR91	protein_coding	0.0006892	2.303
TPK1	protein_coding	0.0060612	2.303
IL15	protein_coding	0.0259512	2.300

MFAP3L	protein_coding	0.0109173	2.298
DGKH	protein_coding	0.000408	2.296
MERTK	protein_coding	0.0028787	2.296
PROS1	protein_coding	0.0008626	2.291
PNPLA7	protein_coding	0.0271977	2.289
TRDMT1	protein_coding	0.00036	2.280
HCG11	lncRNA	0.0036403	2.280
POLG2	protein_coding	0.0081011	2.279
TULP1	protein_coding	0.0312186	2.279
ATP1A1-AS1	lncRNA	5.69E-05	2.276
ODAD2	protein_coding	0.0043387	2.275
HHAT	protein_coding	0.0108344	2.275
ITGA10	protein_coding	0.0026067	2.269
USP49	protein_coding	0.0039449	2.269
ZBTB37	protein_coding	0.000255	2.267
MDGA1	protein_coding	3.78E-09	2.267
SHPRH	protein_coding	9.83E-05	2.267
CTD-2017D11.1		0.007012	2.266
CEMIP2	protein_coding	8.53E-05	2.265
SEMA4F	protein_coding	0.0033751	2.261
ZNF649	protein_coding	0.0040683	2.259
SDK1	protein_coding	2.13E-05	2.259
ACTR3C	protein_coding	0.0065929	2.257
MPP7	protein_coding	0.0125374	2.254
MIR924HG	lncRNA	2.74E-05	2.251
CREB5	protein_coding	0.0035182	2.250
SLC22A15	protein_coding	0.0054535	2.248
PDE4DIPP2	transcribed_unprocessed_pseudogene	0.0001259	2.248
LINC00662	lncRNA	8.84E-06	2.242
BTBD8	protein_coding	0.0042412	2.234
LINC00665	lncRNA	0.0048598	2.231
SNED1	protein_coding	0.0113934	2.231
SPTBN2	protein_coding	0.001552	2.227
PLAAT5	protein_coding	0.0070588	2.224
KDR	protein_coding	0.0339407	2.222
RAB33B-AS1	lncRNA	0.0330131	2.219
VLDLR	protein_coding	4.51E-05	2.219
EIF2AK3	protein_coding	4.22E-06	2.218
KCP	protein_coding	0.0100194	2.217
S1PR3	protein_coding	0.0182073	2.214
SGCE	protein_coding	0.0001257	2.214
SPTBN5	protein_coding	0.0095555	2.212
TPT1-AS1	lncRNA	0.0067772	2.204
ABHD6	protein_coding	0.0105986	2.204
LERFS	lncRNA	0.0007586	2.202
ABCC5	protein_coding	8.72E-05	2.200

CACNA2D1	protein_coding	0.0188869	2.200
KSR1	protein_coding	0.0027684	2.198
CLCN2	protein_coding	0.01093	2.197
PPP1R12B	protein_coding	6.25E-05	2.196
LINC00630	lncRNA	0.0049037	2.196
ELMOD2	protein_coding	0.0162844	2.194
COL17A1	protein_coding	2.75E-05	2.191
SLC39A11	protein_coding	0.0045074	2.182
KCNC4	protein_coding	0.0001505	2.178
TMEM161B-AS1	lncRNA	6.84E-06	2.174
LINC00894	lncRNA	0.0182852	2.174
FBN1	protein_coding	4.04E-05	2.173
FBXL2	protein_coding	0.0045639	2.172
EFCAB5	protein_coding	0.0276103	2.166
PIGO	protein_coding	0.0209705	2.162
MFAP3	protein_coding	0.0012955	2.161
ELF3	protein_coding	0.0143076	2.160
PBX1	protein_coding	3.15E-05	2.160
CKMT2-AS1	lncRNA	0.011858	2.160
SEMA5A	protein_coding	0.0022305	2.159
CCDC136	protein_coding	9.68E-05	2.158
RPS6KA5	protein_coding	5.31E-05	2.153
RAP1GAP	protein_coding	0.0218105	2.153
TNC	protein_coding	5.43E-05	2.152
VPS13B	protein_coding	9.79E-06	2.152
NR4A2	protein_coding	0.0150461	2.149
LINC02228	lncRNA	0.0043105	2.147
DCAF8	protein_coding	0.0036275	2.144
PSEN2	protein_coding	0.039406	2.143
CFAP58	protein_coding	0.0079148	2.138
PLEKHH2	protein_coding	0.0010255	2.138
RP11-1023L17.1		7.00E-05	2.134
STXBP4	protein_coding	0.004224	2.131
RELT	protein_coding	0.0100034	2.131
FLRT2	protein_coding	3.28E-05	2.126
FZD4	protein_coding	0.0166478	2.122
ATXN7L2	protein_coding	0.0180578	2.114
LTBP1	protein_coding	2.17E-05	2.114
ERC2	protein_coding	0.032891	2.114
GOLGA8B	protein_coding	0.0006142	2.103
FJX1	protein_coding	0.0001778	2.100
ENTPD1-AS1	lncRNA	5.58E-05	2.100
ANKRD36B	protein_coding	0.001955	2.100
FAM72A	protein_coding	0.0194389	2.099
FCGBP	protein_coding	0.007543	2.099
CASP10	protein_coding	0.0216686	2.097

GRIK4	protein_coding	0.0006877	2.096
HECW2	protein_coding	5.33E-05	2.092
B4GALNT3	protein_coding	0.0056794	2.091
ZNF26	protein_coding	0.001121	2.091
CELSR2	protein_coding	0.0024618	2.089
NEBL	protein_coding	0.000113	2.088
MAML3	protein_coding	0.0255512	2.083
TOGARAM2	protein_coding	0.0111433	2.083
RALGAPA1	protein_coding	0.0005828	2.082
DIO2	protein_coding	0.0482952	2.080
CCDC18-AS1	lncRNA	0.0141427	2.075
PLEKHS1	protein_coding	0.0180198	2.074
SLC38A7	protein_coding	0.0231663	2.072
ZNF81	protein_coding	0.0044127	2.069
SPRY4-AS1	lncRNA	0.010892	2.068
INTU	protein_coding	0.001362	2.066
AC025171.1		0.0405704	2.064
MICAL3	protein_coding	1.28E-05	2.064
CSF3R	protein_coding	0.0304239	2.063
ENTPD3	protein_coding	0.0263752	2.062
AC021218.2		0.0219152	2.059
PTCH1	protein_coding	0.000467	2.051
FAT4	protein_coding	3.77E-05	2.047
TLR4	protein_coding	0.0064297	2.044
CEP170P1	transcribed_unprocessed_pseudogene	0.0146731	2.043
ITGA9-AS1	lncRNA	0.0002317	2.040
ZNF44	protein_coding	0.0433054	2.039
LAMA3	protein_coding	1.30E-05	2.039
SYNE1	protein_coding	3.86E-08	2.039
ILDR2	protein_coding	0.0010765	2.037
USP35	protein_coding	0.0226852	2.032
HHIP	protein_coding	0.0112833	2.031
PPARGC1A	protein_coding	0.0110174	2.024
SEMA3A	protein_coding	0.001842	2.022
MFSD8	protein_coding	0.0260975	2.021
CH507-513H4.9		0.0074563	2.017
AMY2B	protein_coding	0.0304391	2.016
PRR5L	protein_coding	0.0045707	2.009
PCDHGB2	protein_coding	0.0121965	2.007
SCN5A	protein_coding	0.0002064	2.006
SNX14	protein_coding	0.0129254	2.005
DUBR	lncRNA	0.0019994	2.002
SLC35D1	protein_coding	0.0210995	2.002
MIPOL1	protein_coding	0.0006216	2.002
P4HA2	protein_coding	4.61E-05	1.999
NPIPB5	protein_coding	0.0062138	1.999

EXPH5	protein_coding	4.30E-05	1.997
CNNM2	protein_coding	0.0001408	1.992
SNHG17	lncRNA	0.0003662	1.990
COL12A1	protein_coding	1.49E-07	1.988
LPAL2	transcribed_unprocessed_pseudogene	0.0298866	1.988
ATP10A	protein_coding	0.0084156	1.983
COBLL1	protein_coding	0.0023359	1.983
XYLB	protein_coding	0.0133468	1.982
CRACD	protein_coding	0.0180198	1.981
ZNF619	protein_coding	0.0239645	1.981
PAN2	protein_coding	0.0036829	1.980
SEMA4C	protein_coding	0.0162116	1.978
DPYSL3	protein_coding	0.0042418	1.975
HEMK1	protein_coding	0.0064318	1.974
LIAS	protein_coding	0.0114847	1.971
AHI1	protein_coding	5.04E-05	1.968
SAMD12	protein_coding	0.0006047	1.967
ALPK1	protein_coding	0.0052927	1.966
SLC11A2	protein_coding	0.0014955	1.964
IL17RA	protein_coding	0.0033783	1.963
DHODH	protein_coding	0.0300038	1.961
MYO1D	protein_coding	0.0230625	1.961
ZSCAN12	protein_coding	0.0005233	1.960
CASC2	lncRNA	0.0012949	1.957
MEIS2	protein_coding	0.0245156	1.955
ZNF546	protein_coding	0.0293665	1.953
SLC35A3	protein_coding	0.0014646	1.952
NMNAT3	protein_coding	0.0071916	1.949
GPAM	protein_coding	0.0114385	1.947
CMTR2	protein_coding	0.0147667	1.946
THSD4	protein_coding	0.0020929	1.945
RBM48	protein_coding	0.0290655	1.945
SYNJ1	protein_coding	0.0209144	1.944
RP1-152L7.5		0.0211993	1.941
UNC50	protein_coding	0.008478	1.939
TMEM161B	protein_coding	0.0128143	1.937
SDHA	protein_coding	0.0228293	1.935
CCDC144B	transcribed_unprocessed_pseudogene	0.0073181	1.935
LRRC8C	protein_coding	0.0287711	1.935
ZNF577	protein_coding	0.0009926	1.935
CDKL1	protein_coding	0.0020265	1.933
DGKA	protein_coding	0.007396	1.930
TYW5	protein_coding	0.0143377	1.929
ZSCAN16-AS1	lncRNA	0.0038804	1.928
STARD9	protein_coding	1.18E-05	1.927
KDM7A	protein_coding	0.000638	1.925

GABPB1-AS1	lncRNA	0.0125552	1.925
CRPPA	protein_coding	0.0213509	1.924
COLGALT2	protein_coding	0.0460663	1.918
ASTN2	protein_coding	0.0072718	1.917
LINC00511	lncRNA	2.55E-06	1.917
CLSTN3	protein_coding	0.0453517	1.917
LEPR	protein_coding	0.0439487	1.916
POMT2	protein_coding	0.0010887	1.915
RP11-513I15.6		0.012331	1.913
LNX1	protein_coding	0.049321	1.912
GUSBP1	transcribed_unprocessed_pseudogene	0.0045562	1.909
STON2	protein_coding	0.0463645	1.909
DYNC2H1	protein_coding	0.0010372	1.906
TTC21B	protein_coding	0.0002534	1.903
TERF1	protein_coding	0.0012851	1.901
ZSCAN31	protein_coding	0.001582	1.900
DDX12P	unprocessed_pseudogene	0.0212634	1.898
CHST10	protein_coding	0.0166635	1.898
GSAP	protein_coding	0.0274695	1.898
STK36	protein_coding	0.0136636	1.898
EML1	protein_coding	0.0152195	1.895
PTPN13	protein_coding	0.0005229	1.893
PIGN	protein_coding	0.0124515	1.889
GAB1	protein_coding	0.006942	1.888
RMDN2	protein_coding	0.0177531	1.888
MBTPS2	protein_coding	0.0187571	1.887
MALAT1	lncRNA	1.28E-63	1.887
SEPTIN7-DT	lncRNA	0.0136289	1.881
ENTPD3-AS1	lncRNA	0.0246898	1.880
DNHD1	protein_coding	0.0001883	1.879
FLG-AS1	lncRNA	0.0323807	1.879
CPVL	protein_coding	0.031103	1.879
ABCC10	protein_coding	0.0022394	1.876
PTGER4	protein_coding	0.0351238	1.876
ZNF224	protein_coding	0.0138226	1.876
CPE	protein_coding	0.0464137	1.873
NECTIN1	protein_coding	0.0097779	1.873
PCNX1	protein_coding	5.68E-05	1.868
LINC01505	lncRNA	0.0002077	1.868
MATN2	protein_coding	0.007058	1.868
ZC3H12A	protein_coding	0.0227861	1.864
RTTN	protein_coding	0.0051493	1.862
LGR4	protein_coding	0.0007451	1.859
CCDC7	protein_coding	0.0009213	1.859
COL7A1	protein_coding	0.0002278	1.857
GEN1	protein_coding	0.0013158	1.855

LRRC49	protein_coding	0.0410132	1.855
PPP4R1L	transcribed_unprocessed_pseudogene	0.0027336	1.852
USP13	protein_coding	0.0034081	1.850
SH3PXD2B	protein_coding	0.0054628	1.848
SLC22A5	protein_coding	0.0264283	1.847
SLCO3A1	protein_coding	0.0244036	1.846
DNAH2	protein_coding	3.76E-06	1.843
PSD4	protein_coding	0.0305801	1.843
CCT6B	protein_coding	0.0255592	1.842
CDAN1	protein_coding	0.0254227	1.839
DNM1	protein_coding	0.0095043	1.838
INPP5B	protein_coding	0.0071425	1.838
COL27A1	protein_coding	0.0100635	1.837
GARS1-DT	lncRNA	5.69E-06	1.837
HDAC8	protein_coding	0.0015399	1.836
RP11-214O1.3		0.0476815	1.834
CYP4V2	protein_coding	0.0067653	1.833
ZNF827	protein_coding	3.06E-05	1.833
ZNF250	protein_coding	0.0190657	1.832
TMEM218	protein_coding	0.0034308	1.829
LTBP3	protein_coding	0.0008283	1.828
PGM2L1	protein_coding	0.0035246	1.827
FGD4	protein_coding	0.0360088	1.827
ASB3	protein_coding	0.0095159	1.826
STIM2	protein_coding	0.0080536	1.826
DNAJC18	protein_coding	0.0192971	1.825
AP4S1	protein_coding	0.0399789	1.825
ZNF431	protein_coding	0.006873	1.825
ZNF440	protein_coding	0.0137983	1.823
ZNF862	protein_coding	0.0045978	1.823
ENTPD5	protein_coding	0.0018219	1.822
RNFT2	protein_coding	0.033719	1.818
ST3GAL6	protein_coding	0.0340859	1.814
WWOX	protein_coding	0.0145186	1.813
MTERF1	protein_coding	0.0393771	1.812
MCC	protein_coding	0.0029274	1.811
THAP6	protein_coding	0.0193524	1.809
NEK11	protein_coding	0.0137776	1.808
NOMO2	protein_coding	0.0097133	1.806
COL6A3	protein_coding	0.0002204	1.804
TAF1C	protein_coding	0.0468195	1.803
FGF1	protein_coding	0.0332664	1.800
KIDINS220	protein_coding	8.54E-07	1.799
NFIA	protein_coding	1.07E-08	1.798
SLC12A6	protein_coding	0.0382814	1.798
ZNF704	protein_coding	0.0097236	1.797

ATP6V0A4	protein_coding	0.0126916	1.793
FIG4	protein_coding	0.003684	1.793
SLC5A6	protein_coding	0.0485825	1.790
JPH2	protein_coding	0.0145908	1.789
IL1RAP	protein_coding	0.0104379	1.787
CROCCP2	transcribed_unprocessed_pseudogene	0.0001383	1.786
ITFG2	protein_coding	0.0089587	1.786
KIAA1328	protein_coding	0.0019309	1.786
ZNF200	protein_coding	0.0100194	1.785
IL31RA	protein_coding	0.0043875	1.776
ACAD10	protein_coding	0.0253104	1.775
GPR161	protein_coding	0.0038344	1.774
RAP1GAP2	protein_coding	0.0138226	1.773
FOSB	protein_coding	0.0074468	1.770
COL4A1	protein_coding	0.0036668	1.768
ICA1	protein_coding	0.0393518	1.767
PDE4D	protein_coding	0.0007681	1.766
HEXA	protein_coding	0.0099385	1.763
SYTL2	protein_coding	0.0003518	1.762
ACVR1	protein_coding	0.0183261	1.759
ZFYVE16	protein_coding	0.0020493	1.758
SNORD3C	snoRNA	0.0004117	1.754
ZNF736	protein_coding	0.0291501	1.753
DTNB	protein_coding	0.0203852	1.751
NF1	protein_coding	2.55E-05	1.751
SLC9A8	protein_coding	0.0205606	1.745
ZNF248	protein_coding	0.0309136	1.745
FOXP1	protein_coding	0.0006418	1.743
ZNF518A	protein_coding	0.0080421	1.742
ATP6V0A2	protein_coding	0.0023034	1.742
SQLE	protein_coding	0.0118278	1.740
ZNF605	protein_coding	0.015005	1.737
BDH2	protein_coding	0.0499258	1.737
EXT2	protein_coding	8.59E-05	1.737
CEP126	protein_coding	0.0140524	1.733
DISP1	protein_coding	0.0025801	1.725
P2RY6	protein_coding	0.0223089	1.722
CTC-260E6.6		0.0230412	1.721
ZNF276	protein_coding	0.019065	1.720
KIAA1109	protein_coding	5.26E-07	1.720
SMYD4	protein_coding	0.0364672	1.719
GCNT1	protein_coding	0.0006203	1.719
GALNT6	protein_coding	0.0189957	1.718
ATP6V0A1	protein_coding	0.0097236	1.717
XXYLT1	protein_coding	0.0042412	1.716
PVT1	lncRNA	1.44E-05	1.714

MAST4	protein_coding	8.24E-06	1.714
OSBPL6	protein_coding	0.0229882	1.714
SPEG	protein_coding	0.0169273	1.713
AMMECR1L	protein_coding	0.0021321	1.708
CLCN6	protein_coding	0.0016693	1.708
KNDC1	protein_coding	0.0255981	1.707
NEAT1	lncRNA	4.71E-08	1.707
ZFHX4	protein_coding	0.0007849	1.704
TRMT11	protein_coding	0.0211534	1.703
SLC5A3	protein_coding	0.0158989	1.702
CCDC57	protein_coding	1.90E-05	1.702
TRIM22	protein_coding	0.022386	1.701
B4GALT6	protein_coding	0.0164996	1.701
B4GALT4	protein_coding	0.033566	1.701
AK9	protein_coding	0.0118126	1.698
FOXP2	protein_coding	0.0310254	1.698
RALGAPA2	protein_coding	0.0109362	1.697
HMMR	protein_coding	0.0304391	1.696
SLC9A6	protein_coding	0.0134765	1.696
TNRC6C	protein_coding	1.15E-05	1.693
NRDE2	protein_coding	0.0004824	1.692
SZT2	protein_coding	5.43E-06	1.691
IFTAP	protein_coding	0.0031391	1.691
FANCC	protein_coding	0.0130633	1.688
FAM20C	protein_coding	8.77E-06	1.688
TM2D1	protein_coding	0.0164204	1.686
SEL1L	protein_coding	2.50E-05	1.685
GMDS-DT	lncRNA	8.71E-06	1.683
INSR	protein_coding	0.0021522	1.682
POMT1	protein_coding	0.0027915	1.680
TSPOAP1	protein_coding	0.008478	1.679
MMACHC	protein_coding	0.0409178	1.675
ZNF397	protein_coding	0.0300585	1.673
CTD-2368P22.1		0.0401325	1.670
FST	protein_coding	0.0118892	1.670
GALNT5	protein_coding	0.0019342	1.669
PIK3C2B	protein_coding	0.0143697	1.664
SEPTIN7P14	transcribed_unprocessed_pseudogene	0.0281536	1.663
ZSCAN9	protein_coding	0.0449391	1.663
SCAI	protein_coding	0.0122202	1.662
MTBP	protein_coding	0.0245801	1.662
LINC01006	lncRNA	0.0016086	1.662
MAPK8IP3	protein_coding	1.18E-05	1.657
GNAI1	protein_coding	0.0004341	1.656
ALG14	protein_coding	0.0107991	1.655
DNM3	protein_coding	0.0484374	1.652

MEF2C	protein_coding	0.0043615	1.652
IGFBP1	protein_coding	0.0137718	1.651
SPG7	protein_coding	2.94E-06	1.651
KCNAB2	protein_coding	0.0273973	1.649
TMTC3	protein_coding	0.0273584	1.649
EGR1	protein_coding	1.35E-05	1.648
SLC35B4	protein_coding	0.0080894	1.646
NBEA	protein_coding	0.0030503	1.645
SLC25A12	protein_coding	0.0479778	1.644
TSGA10	protein_coding	0.0292085	1.639
TMEM87B	protein_coding	0.0278197	1.638
STRIP2	protein_coding	0.0055856	1.637
URB1	protein_coding	0.0005263	1.637
MROH8	protein_coding	0.0355803	1.636
LCLAT1	protein_coding	0.0266469	1.635
B3GALT5	protein_coding	0.0005208	1.631
COL13A1	protein_coding	0.0001331	1.630
CAMK4	protein_coding	0.0270831	1.629
CEP85L	protein_coding	0.0486935	1.629
PRDM15	protein_coding	0.0025078	1.628
IQCG	protein_coding	0.0047569	1.628
KIAA1217	protein_coding	1.03E-10	1.628
CENATAC	protein_coding	0.0227861	1.627
LNPEP	protein_coding	0.0019952	1.627
NIPAL1	protein_coding	0.0486935	1.624
KCNQ5	protein_coding	0.0007786	1.624
CTD-2366F13.1		0.0469952	1.623
CCDC146	protein_coding	0.0452717	1.622
JRK	protein_coding	0.0351703	1.620
TICRR	protein_coding	0.0026171	1.619
ADGRL2	protein_coding	0.0025219	1.619
SCARB2	protein_coding	8.53E-05	1.618
KLC1	protein_coding	0.0017327	1.612
DZIP3	protein_coding	0.0032922	1.611
SLC37A3	protein_coding	0.0029942	1.609
PRCP	protein_coding	1.18E-05	1.609
MFSD14A	protein_coding	0.0312205	1.609
DDR2	protein_coding	0.0399232	1.609
PPFIBP2	protein_coding	0.0045356	1.609
FLI1	protein_coding	0.0182791	1.608
PHKA2	protein_coding	0.0087493	1.606
MT-ND6	protein_coding	0.0004759	1.603
ARAP2	protein_coding	0.0185829	1.603
ZRANB3	protein_coding	0.0408197	1.602
EP400P1	transcribed_unprocessed_pseudogene	0.0346973	1.601
ATP8B1	protein_coding	0.0397305	1.598

RP11-384K6.6		0.0486103	1.595
RFX3	protein_coding	0.0416672	1.595
LIPG	protein_coding	0.0069133	1.595
SCYL3	protein_coding	0.0360547	1.591
PTPRE	protein_coding	0.0122716	1.590
ARSJ	protein_coding	0.0211398	1.587
TTF2	protein_coding	0.0008921	1.586
WDR19	protein_coding	0.013871	1.585
DNAJC16	protein_coding	0.0077223	1.585
PABPN1	protein_coding	0.0026746	1.582
BICD1	protein_coding	0.0158989	1.579
WDR59	protein_coding	0.0342688	1.578
CHST2	protein_coding	0.0184062	1.576
LARS2	protein_coding	0.0012417	1.574
ADGRG6	protein_coding	0.0235878	1.573
THADA	protein_coding	0.0016101	1.571
LRRC27	protein_coding	0.0087402	1.571
FOS	protein_coding	0.0033087	1.571
WNK3	protein_coding	0.0259326	1.567
ATP8B3	protein_coding	0.0367322	1.566
ZNF354C	protein_coding	0.0190657	1.560
SUCLG2-AS1	lncRNA	0.0158127	1.557
LINC02609	lncRNA	0.0456149	1.556
RRBP1	protein_coding	3.76E-19	1.553
MCM9	protein_coding	0.0319194	1.549
ERAP1	protein_coding	0.001143	1.547
RSRP1	protein_coding	0.0032313	1.546
CTC1	protein_coding	0.0401763	1.544
NLRC5	protein_coding	0.0041344	1.544
SNX29	protein_coding	0.0134244	1.544
LPIN3	protein_coding	0.0158777	1.544
RP11-796E2.4		0.0213606	1.541
G2E3	protein_coding	0.0048042	1.541
CDON	protein_coding	0.0156326	1.541
LHFPL6	protein_coding	0.0253764	1.534
ACSS3	protein_coding	0.0323811	1.529
CHST12	protein_coding	0.0105724	1.525
IFT172	protein_coding	0.0006338	1.520
CFAP251	protein_coding	0.013964	1.518
ARHGAP26	protein_coding	0.0001598	1.518
RASD1	protein_coding	0.0103285	1.517
RP1L1	protein_coding	0.0463858	1.516
SLC1A4	protein_coding	0.0399789	1.514
PRDM10	protein_coding	0.004106	1.511
NOS1AP	protein_coding	0.0216112	1.509
FAM3C2P	processed_pseudogene	0.0117985	1.507

ZMYM2	protein_coding	0.0191615	1.507
ST3GAL1	protein_coding	0.0031845	1.504
CTSL	protein_coding	1.39E-05	1.500
PIK3R1	protein_coding	0.0062018	1.500
MPP3	protein_coding	0.0384835	1.500
NDC1	protein_coding	0.0158777	1.499
SEMA3B	protein_coding	0.0359917	1.498
TMX3	protein_coding	0.0396838	1.496
DNAJB14	protein_coding	0.0013158	1.496
KIFAP3	protein_coding	0.0096769	1.495
NCSTN	protein_coding	0.0070097	1.494
GK5	protein_coding	0.042033	1.494
TBXAS1	protein_coding	0.022048	1.493
MMP1	protein_coding	0.0117451	1.492
PHGDH	protein_coding	0.0228895	1.491
SNAPC4	protein_coding	0.0011528	1.490
FKTN	protein_coding	0.003305	1.490
TRIM2	protein_coding	0.0473835	1.485
PLEKHM1P1	transcribed_unprocessed_pseudogene	0.044014	1.485
CUL9	protein_coding	8.32E-05	1.484
SCARB1	protein_coding	0.0336083	1.484
TYW1	protein_coding	0.0130015	1.483
TMEM185B	protein_coding	0.0231281	1.483
INCENP	protein_coding	5.12E-23	1.482
ZBED5	protein_coding	0.0367071	1.482
MGAT2	protein_coding	2.21E-05	1.482
ZDHHC17	protein_coding	0.0473098	1.481
TMEM63B	protein_coding	0.0026736	1.479
ATAD2B	protein_coding	0.0199608	1.478
DOCK2	protein_coding	0.0011625	1.477
MLH3	protein_coding	0.0262605	1.474
F2RL1	protein_coding	0.0015204	1.469
BRD9	protein_coding	1.52E-08	1.467
DTWD1	protein_coding	0.0266469	1.466
MOCS1	protein_coding	0.0254172	1.465
KIAA1549	protein_coding	0.0037037	1.465
LDLR	protein_coding	3.35E-16	1.462
NR3C2	protein_coding	0.0421562	1.461
LBR	protein_coding	0.0002508	1.459
CREBZF	protein_coding	0.000742	1.459
NBPF12	protein_coding	0.0078716	1.458
ZFYVE26	protein_coding	8.59E-05	1.457
METTL22	protein_coding	0.0486584	1.452
TCP11L2	protein_coding	0.0478652	1.452
GCNT2	protein_coding	4.38E-06	1.449
TPM2	protein_coding	0.0043952	1.448

CCNL1	protein_coding	0.0202244	1.446
ATF3	protein_coding	0.0006483	1.446
LTO1	protein_coding	0.0458951	1.445
ULK4	protein_coding	0.0089754	1.443
ALG5	protein_coding	0.0238053	1.443
GS1-124K5.12		0.0289097	1.443
BTD	protein_coding	0.0386875	1.442
ATP6AP2	protein_coding	0.0018412	1.442
BDNF	protein_coding	0.0006041	1.440
ZNF528-AS1	lncRNA	0.0423394	1.439
ERCC8	protein_coding	0.0013046	1.438
FAM13A	protein_coding	0.0069087	1.435
C2CD2L	protein_coding	0.0224038	1.435
ATRN	protein_coding	0.0028026	1.427
LRSAM1	protein_coding	0.0020518	1.426
PRDM11	protein_coding	0.0270993	1.426
POMK	protein_coding	0.033566	1.425
BTBD9	protein_coding	0.0104069	1.423
HMBOX1	protein_coding	0.0308228	1.423
FUCA1	protein_coding	0.0259326	1.422
DEPDC1B	protein_coding	0.0049399	1.419
COQ8B	protein_coding	0.0452644	1.418
DNMT3B	protein_coding	0.0417464	1.417
CTNND1	protein_coding	0.0161017	1.416
LRP12	protein_coding	0.0461594	1.416
CEP57L1	protein_coding	0.033566	1.414
LUCAT1	lncRNA	0.0242589	1.409
PCBP1-AS1	lncRNA	0.0004169	1.406
HKDC1	protein_coding	0.0171279	1.406
TRAF1	protein_coding	0.0287333	1.406
NBEAL1	protein_coding	0.0051379	1.405
ATR	protein_coding	0.005977	1.405
VPS13D	protein_coding	3.89E-05	1.404
MIA3	protein_coding	0.0002877	1.404
C20orf194	protein_coding	0.0461781	1.401
ROBO4	protein_coding	0.0152991	1.401
SETD4	protein_coding	0.0368965	1.399
IL4R	protein_coding	0.027907	1.398
SRGAP1	protein_coding	0.0008806	1.397
TRAF5	protein_coding	0.0486416	1.395
UTP20	protein_coding	0.0012169	1.394
HYOU1	protein_coding	1.20E-12	1.393
WDR47	protein_coding	0.0410691	1.392
ZNF333	protein_coding	0.0176241	1.389
KIF24	protein_coding	0.0275618	1.389
FAM135A	protein_coding	0.0173378	1.387

EEA1	protein_coding	3.81E-05	1.387
CROCC	protein_coding	0.0140333	1.387
DSE	protein_coding	0.0247071	1.387
FAM126B	protein_coding	0.0382574	1.385
UBR3	protein_coding	0.039406	1.383
NOTCH2	protein_coding	2.36E-07	1.382
TSPAN3	protein_coding	0.0006062	1.381
POR	protein_coding	0.0084038	1.380
CTSC	protein_coding	0.0056692	1.376
RHBDF2	protein_coding	0.0121565	1.375
BCAS3	protein_coding	0.0332994	1.374
ABCB9	protein_coding	0.0345811	1.373
DMXL1	protein_coding	0.0202131	1.370
UGGT1	protein_coding	0.0008367	1.370
ZNF394	protein_coding	0.008801	1.367
MX2	protein_coding	0.0196196	1.364
TRPS1	protein_coding	0.0247442	1.361
FZD6	protein_coding	0.025432	1.358
MESD	protein_coding	0.0332478	1.357
KLHDC4	protein_coding	0.0094992	1.356
ITPR2	protein_coding	0.0007956	1.355
DHX57	protein_coding	0.0119264	1.354
MFSD14C	transcribed_unprocessed_pseudogene	0.0230412	1.351
DDX60L	protein_coding	0.0054669	1.351
EXOC6	protein_coding	0.0423689	1.348
TRAPPC9	protein_coding	0.0251128	1.343
ADAMTS6	protein_coding	0.0442045	1.342
ATP10D	protein_coding	0.0076691	1.341
CBX4	protein_coding	0.0002272	1.341
SC5D	protein_coding	0.0145908	1.339
NLRP1	protein_coding	0.0117747	1.339
ATAD1	protein_coding	0.029646	1.339
PLB1	protein_coding	0.0403082	1.337
MIR100HG	lncRNA	0.0002382	1.336
CPT1A	protein_coding	0.0330972	1.335
LMO7	protein_coding	2.03E-05	1.334
CEPT1	protein_coding	0.0490894	1.333
MAP2	protein_coding	0.0332487	1.332
PLXNA2	protein_coding	0.0026989	1.332
KDM4A	protein_coding	0.0213509	1.330
CANT1	protein_coding	5.84E-05	1.328
PEAK1	protein_coding	0.0052645	1.328
AKAP9	protein_coding	2.55E-06	1.327
TIAM2	protein_coding	0.0181019	1.327
ALS2	protein_coding	0.0040972	1.326
PTPRM	protein_coding	0.000559	1.326

LINC00261	lncRNA	0.031317	1.326
NOTCH1	protein_coding	0.0001587	1.323
TRANK1	protein_coding	0.0230711	1.322
ATG2B	protein_coding	0.0034336	1.322
ZFAT	protein_coding	0.0076606	1.321
LINC00963	lncRNA	0.0188644	1.320
LMLN	protein_coding	0.0343186	1.319
STK33	protein_coding	0.0436691	1.319
F2R	protein_coding	0.0430409	1.317
SFXN5	protein_coding	0.0249609	1.315
VPS13A	protein_coding	0.0043155	1.314
ITGAV	protein_coding	0.0299013	1.312
ZNF398	protein_coding	0.0049734	1.308
OSER1-DT	lncRNA	0.0483049	1.305
GNPTAB	protein_coding	0.0425454	1.305
SUN2	protein_coding	0.0065106	1.304
TMEM63A	protein_coding	0.0183792	1.303
RABGAP1L	protein_coding	0.0017985	1.301
LINC01322	lncRNA	0.0036482	1.300
PRKCE	protein_coding	0.0102849	1.299
SYNE2	protein_coding	4.45E-08	1.298
CLCC1	protein_coding	0.0488547	1.296
PCSK7	protein_coding	0.0018922	1.295
PXDN	protein_coding	9.50E-05	1.290
CREBRF	protein_coding	0.0065929	1.289
SLC8B1	protein_coding	0.014045	1.289
SLX4	protein_coding	0.0228064	1.289
C2CD3	protein_coding	0.0003061	1.287
PIGG	protein_coding	0.0098289	1.282
NAV2	protein_coding	2.97E-12	1.282
VANGL1	protein_coding	0.0025219	1.281
ESPL1	protein_coding	0.0210475	1.281
PKD1	protein_coding	0.0130922	1.281
SLC3A2	protein_coding	1.40E-06	1.281
INO80D	protein_coding	0.0139426	1.278
HSP90B1	protein_coding	3.84E-20	1.276
BRPF1	protein_coding	0.0007758	1.275
MBD5	protein_coding	0.0289013	1.275
CC2D2A	protein_coding	0.0259282	1.275
ZNF331	protein_coding	0.0155556	1.274
CHD6	protein_coding	4.23E-08	1.267
HIVEP1	protein_coding	0.0012049	1.266
PLCE1	protein_coding	0.0249609	1.263
COL8A1	protein_coding	0.0276543	1.260
UBN2	protein_coding	0.0141427	1.260
IGHMBP2	protein_coding	0.0308227	1.256

SNORA73B	snoRNA	4.83E-06	1.256
SLC35F5	protein_coding	0.0143441	1.254
DSG2	protein_coding	0.0300038	1.251
HSPA5	protein_coding	2.90E-11	1.248
LMBR1	protein_coding	0.0268698	1.247
C6orf89	protein_coding	0.0068994	1.247
NPHP4	protein_coding	0.0204683	1.244
ADCY9	protein_coding	0.0299833	1.244
FANCD2	protein_coding	0.0225011	1.241
WLS	protein_coding	0.0410501	1.239
ERBB2	protein_coding	0.0100667	1.239
BICD2	protein_coding	7.43E-05	1.238
PLOD3	protein_coding	0.0171353	1.236
SYVN1	protein_coding	0.0194774	1.236
HECTD4	protein_coding	0.0001218	1.235
CCDC88C	protein_coding	0.000886	1.233
DOCK4	protein_coding	0.0143652	1.232
SLC7A11	protein_coding	0.0176596	1.232
TSPYL2	protein_coding	0.002943	1.232
PKD2	protein_coding	0.018521	1.229
PCNX2	protein_coding	0.0191612	1.228
CARD8	protein_coding	0.0060991	1.226
CARMIL1	protein_coding	0.0137801	1.225
ADAM9	protein_coding	6.12E-07	1.225
TNFAIP8L1	protein_coding	0.0273584	1.223
ARL6IP1	protein_coding	0.000601	1.222
ADAM10	protein_coding	0.0009896	1.220
KIAA2013	protein_coding	0.0004203	1.220
POU2F1	protein_coding	0.0204931	1.217
SASH1	protein_coding	0.032715	1.214
OSMR	protein_coding	0.0127467	1.214
CSPP1	protein_coding	0.0037275	1.214
NEGR1	protein_coding	0.0438508	1.214
XPR1	protein_coding	0.0349014	1.213
LRP8	protein_coding	0.0269818	1.210
CEP350	protein_coding	7.39E-06	1.209
SOGA1	protein_coding	4.60E-06	1.207
ERLIN1	protein_coding	0.0162689	1.207
GPATCH2L	protein_coding	0.000754	1.206
PLEKHA1	protein_coding	0.0362648	1.205
IGF2R	protein_coding	5.01E-05	1.203
UBR1	protein_coding	0.0392783	1.203
TRAF2	protein_coding	0.020452	1.202
RN7SKP203	misc_RNA	0.0007836	1.202
NKTR	protein_coding	0.0002704	1.201
ZSWIM8	protein_coding	0.0126175	1.200

PIEZO1	protein_coding	5.98E-06	1.200
GCLC	protein_coding	0.0177596	1.196
DMXL2	protein_coding	0.0498097	1.193
CHST3	protein_coding	0.0335268	1.190
COL4A2	protein_coding	0.0026165	1.190
SPTLC2	protein_coding	0.0343996	1.189
RBM47	protein_coding	0.0065804	1.189
FZD7	protein_coding	0.0182071	1.188
RBM19	protein_coding	5.36E-05	1.188
ILVBL	protein_coding	0.0466363	1.188
GALNT2	protein_coding	0.000559	1.185
EMC1	protein_coding	0.013286	1.180
BAHCC1	protein_coding	0.0279864	1.180
PHLDB1	protein_coding	0.0001674	1.179
PRKRIP1	protein_coding	0.0277008	1.178
FNIP2	protein_coding	0.0331042	1.178
TSPAN14	protein_coding	0.0190891	1.176
LIFR	protein_coding	0.0187927	1.176
ABCC3	protein_coding	0.0190012	1.171
CDKN2D	protein_coding	0.0202244	1.168
CCNL2	protein_coding	0.0111433	1.166
JADE1	protein_coding	0.0100975	1.165
HEATR1	protein_coding	0.0379262	1.160
PIM3	protein_coding	0.0337676	1.158
ICAM1	protein_coding	0.0418968	1.156
ZNF160	protein_coding	0.0274695	1.156
ADGRA3	protein_coding	0.0151466	1.155
LRP1	protein_coding	0.0208363	1.149
LAPTM4A	protein_coding	0.0421435	1.148
ZBTB40	protein_coding	0.0077629	1.148
SIL1	protein_coding	0.0426052	1.147
ATL2	protein_coding	0.0055856	1.145
NR2F1-AS1	lncRNA	0.0229483	1.145
KIF18A	protein_coding	0.0056164	1.145
ROR1	protein_coding	0.0206769	1.144
ASPM	protein_coding	3.73E-06	1.142
STT3A	protein_coding	0.002679	1.142
BPNT2	protein_coding	0.0185525	1.141
NFE2L3	protein_coding	0.0086267	1.136
TNKS1BP1	protein_coding	0.0023939	1.135
CLASP2	protein_coding	0.0084703	1.130
DDHD1	protein_coding	0.0162479	1.129
KIF14	protein_coding	5.93E-05	1.128
VPS8	protein_coding	0.0254815	1.127
DNAH14	protein_coding	0.0032031	1.125
HRH1	protein_coding	0.0365045	1.124

AP1AR	protein_coding	0.029646	1.123
ATP11A	protein_coding	0.0129196	1.123
PLEKHO1	protein_coding	0.01568	1.123
DENND2B	protein_coding	0.0300903	1.122
CLSTN1	protein_coding	9.29E-05	1.121
ZNF202	protein_coding	0.0314547	1.120
CDH11	protein_coding	0.0046274	1.118
PCNX4	protein_coding	0.0002424	1.118
UXS1	protein_coding	0.0051863	1.116
EPB41	protein_coding	0.0070076	1.114
SHANK2	protein_coding	0.0146648	1.112
SLC7A5	protein_coding	6.53E-05	1.111
STARD8	protein_coding	0.0228367	1.109
TTC17	protein_coding	0.0070335	1.106
RGS3	protein_coding	0.0026116	1.106
SPPL3	protein_coding	0.0458681	1.105
PLXNB2	protein_coding	0.0004895	1.104
REEP4	protein_coding	0.0162907	1.101
SNX13	protein_coding	0.0214942	1.099
ODR4	protein_coding	0.0146731	1.098
FILIP1L	protein_coding	0.0351918	1.097
ARMC9	protein_coding	0.0252328	1.091
TMEM30A	protein_coding	0.0009324	1.090
TMEM127	protein_coding	0.0406839	1.088
CD46	protein_coding	0.0080402	1.087
NAV3	protein_coding	0.0218179	1.084
MTDH	protein_coding	1.46E-06	1.083
JAG1	protein_coding	0.0029243	1.082
SH3RF3	protein_coding	0.0409801	1.081
CDC25C	protein_coding	0.0324668	1.080
KDM3A	protein_coding	0.0014129	1.080
CCDC88A	protein_coding	6.83E-05	1.076
TVP23B	protein_coding	0.039922	1.073
ATP2A2	protein_coding	4.16E-05	1.072
AGRN	protein_coding	0.0009371	1.072
CENPE	protein_coding	8.30E-06	1.071
KMT2B	protein_coding	8.54E-05	1.069
PVR	protein_coding	0.0411411	1.068
SPDL1	protein_coding	0.0021321	1.067
SGMS1	protein_coding	0.0451782	1.066
JARID2	protein_coding	0.0007901	1.066
VEGFA	protein_coding	9.18E-06	1.066
ZC3H4	protein_coding	0.000576	1.064
PTPRG	protein_coding	0.002081	1.064
LAMB1	protein_coding	0.0251929	1.063
RBBP6	protein_coding	0.0049734	1.062

PLEKHG3	protein_coding	0.0129835	1.060
SLC38A10	protein_coding	0.0405704	1.058
KBTBD2	protein_coding	0.0209144	1.057
DEF6	protein_coding	0.0056578	1.057
HMGCR	protein_coding	0.0214821	1.056
TSC1	protein_coding	0.0007231	1.056
LAMA5	protein_coding	8.37E-05	1.054
TNIK	protein_coding	0.0182457	1.053
CRIM1	protein_coding	0.0001775	1.052
DYRK1A	protein_coding	0.0195198	1.052
MIR4435-2HG	lncRNA	0.0007508	1.051
CEP295	protein_coding	0.0332145	1.050
CEP95	protein_coding	0.0373043	1.050
CWF19L2	protein_coding	0.0429105	1.048
ABCA1	protein_coding	0.0030162	1.044
EPHB2	protein_coding	0.0120943	1.041
SACM1L	protein_coding	0.034206	1.041
GOLGA1	protein_coding	0.0200997	1.040
BRWD1	protein_coding	0.0085323	1.039
CEP85	protein_coding	0.0223724	1.038
DBF4B	protein_coding	0.007455	1.034
NBPF1	protein_coding	0.013964	1.034
NPC1	protein_coding	0.0037125	1.033
HEG1	protein_coding	0.0007042	1.033
PTPRF	protein_coding	0.0027568	1.032
GPR107	protein_coding	0.027214	1.029
LUC7L3	protein_coding	3.95E-10	1.029
ATP11B	protein_coding	0.0194499	1.026
TACC3	protein_coding	2.38E-07	1.026
POLK	protein_coding	0.0318129	1.023
RUFY3	protein_coding	0.0197207	1.023
DDRGK1	protein_coding	0.0072699	1.022
NEDD9	protein_coding	0.0145985	1.022
BRAF	protein_coding	0.0073217	1.017
APP	protein_coding	1.26E-07	1.016
CLCN4	protein_coding	0.0423039	1.016
FAM3C	protein_coding	0.0003652	1.014
PTPRS	protein_coding	0.0045141	1.014
INPP4A	protein_coding	0.0260916	1.014
RBM5	protein_coding	0.0370909	1.013
SEMA3C	protein_coding	0.016188	1.012
GON4L	protein_coding	0.0014922	1.011
SGPL1	protein_coding	0.0375753	1.011
SUGP2	protein_coding	0.0011966	1.010
THBS1	protein_coding	6.02E-06	1.009
INTS6	protein_coding	0.0314827	1.007

IGF1R	protein_coding	0.0371498	1.006
ITGA3	protein_coding	9.58E-12	1.005
PAX8-AS1	lncRNA	0.0293765	1.004
SLC25A25	protein_coding	0.0419733	1.004
CD9	protein_coding	0.0160119	1.000
SFN	protein_coding	0.0003814	-1.002
R3HDM4	protein_coding	0.0077223	-1.004
NSMCE1	protein_coding	0.0339967	-1.004
SNRPB	protein_coding	0.000733	-1.006
RAB31	protein_coding	0.0001102	-1.007
TOX2	protein_coding	0.0009411	-1.007
TRIM44	protein_coding	9.73E-07	-1.007
FBXW5	protein_coding	2.84E-05	-1.007
MLX	protein_coding	0.0005664	-1.007
STOML2	protein_coding	5.00E-05	-1.008
PABPC4	protein_coding	3.50E-08	-1.009
MAPK1IP1L	protein_coding	3.83E-05	-1.009
UBE2J1	protein_coding	0.0031703	-1.010
EIF3H	protein_coding	1.69E-05	-1.011
PTOV1	protein_coding	0.0019154	-1.011
DCAKD	protein_coding	0.0304219	-1.013
RNVU1-31	snRNA	1.85E-05	-1.014
CLSPN	protein_coding	1.99E-06	-1.014
PRPF38B	protein_coding	4.84E-06	-1.015
MYEOV	protein_coding	0.0003201	-1.015
NDUFAF8	protein_coding	3.96E-05	-1.015
SEPTIN10	protein_coding	7.55E-05	-1.016
PSMB7	protein_coding	3.77E-05	-1.016
MT1E	protein_coding	0.0323135	-1.016
UQCRB	protein_coding	1.01E-06	-1.018
COPG1	protein_coding	6.54E-06	-1.018
PEBP1	protein_coding	2.36E-05	-1.018
TRIM16L	transcribed_unprocessed_pseudogene	0.0270232	-1.019
RN7SL4P	misc_RNA	0.0208832	-1.019
DDAH1	protein_coding	6.65E-05	-1.019
LSM4	protein_coding	2.51E-07	-1.020
COPZ1	protein_coding	0.0074799	-1.021
SHMT2	protein_coding	0.0002741	-1.023
PRDX1	protein_coding	1.99E-07	-1.023
ANXA2P2	processed_pseudogene	0.0001925	-1.024
MEA1	protein_coding	1.21E-09	-1.024
PTPN18	protein_coding	0.0231281	-1.025
GLTP	protein_coding	0.0003905	-1.025
CNN2	protein_coding	1.70E-08	-1.028
TIMM8B	protein_coding	0.0020491	-1.028
MRPS10	protein_coding	7.21E-06	-1.028

RPL10A	protein_coding	1.17E-09	-1.028
DPYSL2	protein_coding	4.39E-09	-1.029
NDUFS4	protein_coding	0.0004743	-1.031
PEX10	protein_coding	0.0182457	-1.032
UFM1	protein_coding	0.0012422	-1.033
RPL23A	protein_coding	1.42E-21	-1.033
PDLIM4	protein_coding	0.0459081	-1.033
PRDX5	protein_coding	5.86E-06	-1.033
TMSB10	protein_coding	1.17E-21	-1.034
C12orf57	protein_coding	0.0048328	-1.035
TIMM13	protein_coding	0.000389	-1.035
MCM6	protein_coding	0.0260422	-1.036
RPS24	protein_coding	9.18E-09	-1.036
PARK7	protein_coding	3.32E-06	-1.037
MRPL52	protein_coding	0.0007119	-1.038
GPI	protein_coding	8.98E-05	-1.040
SEC13	protein_coding	2.60E-07	-1.040
UROD	protein_coding	0.000974	-1.041
TPI1	protein_coding	9.74E-16	-1.042
ATP5F1A	protein_coding	7.72E-07	-1.042
HMGB1P6	processed_pseudogene	0.003853	-1.042
OXA1L	protein_coding	0.0113802	-1.042
MLYCD	protein_coding	0.0206079	-1.043
COX4I1	protein_coding	1.97E-08	-1.043
CCDC25	protein_coding	0.0017043	-1.043
LDHA	protein_coding	4.71E-16	-1.043
PGK1	protein_coding	4.32E-10	-1.044
MYL6	protein_coding	8.21E-06	-1.047
SNRPA	protein_coding	0.000924	-1.047
CDK2AP2	protein_coding	0.0005636	-1.048
LCMT1	protein_coding	0.049922	-1.048
AIP	protein_coding	0.0015441	-1.048
YPEL5	protein_coding	5.42E-05	-1.051
RAB34	protein_coding	0.0003071	-1.051
IK	protein_coding	7.03E-08	-1.053
BANF1	protein_coding	0.0010868	-1.053
NENF	protein_coding	0.0039182	-1.053
TUBA1B	protein_coding	6.91E-21	-1.053
GNG12	protein_coding	3.70E-06	-1.053
NAA38	protein_coding	0.0074047	-1.054
ACAT2	protein_coding	0.0003418	-1.054
EIF5A	protein_coding	1.78E-12	-1.054
GOT2	protein_coding	0.0007072	-1.055
SOCS4	protein_coding	0.0034336	-1.055
GGA2	protein_coding	0.0128185	-1.057
APEX1	protein_coding	0.0001882	-1.057

SKA2	protein_coding	3.13E-08	-1.057
ORMDL3	protein_coding	0.0342515	-1.057
SNHG16	lncRNA	0.0001365	-1.057
NUDT15	protein_coding	0.0346687	-1.058
ENO1	protein_coding	7.56E-19	-1.058
CYB5B	protein_coding	0.0034344	-1.058
UQCR10	protein_coding	0.0023615	-1.058
AHCY	protein_coding	1.72E-05	-1.061
RPL15	protein_coding	1.99E-13	-1.062
SERINC2	protein_coding	0.0158777	-1.062
KRT18	protein_coding	4.53E-14	-1.063
DSTN	protein_coding	2.04E-12	-1.063
SPTLC3	protein_coding	0.001367	-1.064
PA2G4	protein_coding	0.0017238	-1.064
LRRC42	protein_coding	0.0107437	-1.064
AKR1C3	protein_coding	0.0002375	-1.066
VDAC1	protein_coding	1.01E-07	-1.066
NDUFS7	protein_coding	0.0147633	-1.066
DGCR6L	protein_coding	0.0148578	-1.066
MRPS15	protein_coding	2.97E-05	-1.067
RWDD1	protein_coding	0.0004205	-1.067
SRP14	protein_coding	8.21E-09	-1.068
ARL2	protein_coding	0.0477498	-1.068
DAP	protein_coding	1.46E-11	-1.068
BCKDK	protein_coding	0.0157979	-1.069
WDR54	protein_coding	0.0412385	-1.069
EIF4B	protein_coding	2.22E-07	-1.070
VAMP3	protein_coding	0.0002508	-1.072
WDR43	protein_coding	1.87E-06	-1.073
LSM7	protein_coding	0.0346741	-1.073
PYCR1	protein_coding	0.0004276	-1.074
ACTB	protein_coding	9.42E-31	-1.075
SNX2	protein_coding	0.0003097	-1.076
IFT20	protein_coding	0.0211142	-1.076
ROMO1	protein_coding	0.0001325	-1.076
CENPI	protein_coding	0.0029532	-1.076
EIF2S3	protein_coding	5.47E-07	-1.077
MRPS16	protein_coding	0.0071076	-1.078
BEX3	protein_coding	1.59E-05	-1.079
RP11-54H7.4		0.0013607	-1.080
GADD45GIP1	protein_coding	1.56E-07	-1.081
NFU1	protein_coding	0.0142464	-1.082
CARD19	protein_coding	0.0024749	-1.083
COX7C	protein_coding	5.59E-14	-1.083
ACTR1B	protein_coding	0.0031261	-1.084
CFL2	protein_coding	0.0037275	-1.084

PPP1R14B	protein_coding	2.57E-07	-1.084
KLHDC3	protein_coding	3.95E-05	-1.085
PDHB	protein_coding	0.0003607	-1.085
BUD31	protein_coding	0.0002169	-1.086
COA3	protein_coding	0.0176596	-1.090
ELOF1	protein_coding	0.0094361	-1.090
IPO7	protein_coding	1.71E-09	-1.090
AP2S1	protein_coding	0.000578	-1.091
ATP5F1C	protein_coding	4.36E-06	-1.091
POLR2C	protein_coding	0.0094517	-1.092
RPS25	protein_coding	1.17E-14	-1.094
RAD23A	protein_coding	4.62E-07	-1.095
CENPX	protein_coding	0.0158328	-1.096
MRPL11	protein_coding	0.0019036	-1.096
PPIL1	protein_coding	0.0003242	-1.097
SLIRP	protein_coding	0.0024382	-1.097
RPL14P1	processed_pseudogene	0.0418968	-1.098
SZRD1	protein_coding	2.43E-06	-1.098
UQCRC1	protein_coding	3.60E-05	-1.099
SLC2A4RG	protein_coding	0.000411	-1.099
EEF2	protein_coding	1.61E-16	-1.100
EIF4EBP2	protein_coding	2.30E-05	-1.100
POLR2J	protein_coding	9.70E-07	-1.101
STMN3	protein_coding	0.0435957	-1.102
NHS	protein_coding	0.0255512	-1.102
VEGFB	protein_coding	5.05E-08	-1.103
COX7A2	protein_coding	0.001497	-1.103
FAM210B	protein_coding	0.0253764	-1.104
CMBL	protein_coding	0.0016086	-1.105
TUBA1A	protein_coding	1.88E-05	-1.105
EIF6	protein_coding	5.14E-05	-1.106
TMSB4X	protein_coding	4.61E-18	-1.107
OLA1	protein_coding	5.01E-08	-1.109
MTIF3	protein_coding	0.0080273	-1.109
PLPPR2	protein_coding	0.0381057	-1.109
ATP6V1F	protein_coding	0.0008187	-1.109
PSMB5	protein_coding	0.0023539	-1.110
TOMM22	protein_coding	0.0308445	-1.111
NDUFB4	protein_coding	0.0364229	-1.116
ELOB	protein_coding	3.77E-09	-1.116
ECSIT	protein_coding	0.0091578	-1.116
MDH2	protein_coding	2.05E-08	-1.118
YEATS4	protein_coding	0.0413046	-1.119
PKIG	protein_coding	7.86E-05	-1.119
CSDE1	protein_coding	1.23E-18	-1.120
RGS10	protein_coding	1.68E-05	-1.124

CHRAC1	protein_coding	0.0027292	-1.124
BCL2L1	protein_coding	4.87E-11	-1.124
TMEM230	protein_coding	1.92E-05	-1.125
PPP1R13L	protein_coding	0.0072509	-1.128
RBMS2	protein_coding	0.0002317	-1.132
CORO1B	protein_coding	9.95E-08	-1.132
SERPINB1	protein_coding	9.55E-06	-1.133
TXN	protein_coding	2.35E-08	-1.135
PFN1	protein_coding	4.89E-19	-1.135
CAT	protein_coding	0.0050382	-1.135
MRPL21	protein_coding	0.002984	-1.137
NPM3	protein_coding	0.0267304	-1.138
SHARPIN	protein_coding	0.0006151	-1.139
POLR2L	protein_coding	4.95E-06	-1.140
UQCRQ	protein_coding	1.93E-08	-1.140
RPL3P4	processed_pseudogene	0.0018135	-1.142
POLD2	protein_coding	8.69E-07	-1.142
SCRN1	protein_coding	2.58E-07	-1.142
FPGS	protein_coding	0.0074957	-1.143
G6PD	protein_coding	2.01E-09	-1.145
ARMCX3	protein_coding	0.004957	-1.146
PAFAH1B3	protein_coding	0.0052858	-1.147
NPRL3	protein_coding	0.004164	-1.147
RNF11	protein_coding	1.57E-05	-1.147
MICOS13	protein_coding	0.0211142	-1.148
PAXX	protein_coding	0.0213272	-1.150
POLR2G	protein_coding	0.007209	-1.150
ARHGDIB	protein_coding	2.11E-07	-1.150
NUBP2	protein_coding	0.0354194	-1.150
RPL36	protein_coding	2.46E-14	-1.150
ELK1	protein_coding	0.0005239	-1.151
BTBD2	protein_coding	0.0009152	-1.151
PHB2	protein_coding	4.63E-06	-1.153
RHOD	protein_coding	0.0398949	-1.153
TNS3	protein_coding	8.58E-09	-1.154
CCDC9B	protein_coding	4.02E-06	-1.154
DMAC2	protein_coding	0.0050526	-1.154
CHMP3	protein_coding	0.0033783	-1.156
ARPC3	protein_coding	9.81E-08	-1.157
FIBP	protein_coding	6.00E-05	-1.158
RPL6	protein_coding	1.91E-17	-1.160
UBE2N	protein_coding	0.0002783	-1.160
CDK4	protein_coding	1.74E-05	-1.160
S100A4	protein_coding	2.06E-09	-1.161
PAICS	protein_coding	5.90E-08	-1.162
BTF3	protein_coding	1.94E-12	-1.162

TIMM17B	protein_coding	0.0013849	-1.162
NBPF26	protein_coding	0.0127936	-1.163
BCAT1	protein_coding	1.66E-05	-1.165
RP11-181C21.4		0.0162962	-1.166
RPS13	protein_coding	1.65E-11	-1.166
COMMD6	protein_coding	0.0110162	-1.167
RPSAP47	processed_pseudogene	0.0236594	-1.168
UFC1	protein_coding	8.34E-05	-1.169
POLE4	protein_coding	0.0168648	-1.170
NBPF20	protein_coding	0.0018214	-1.171
COPS3	protein_coding	2.23E-05	-1.173
C19orf48	transcribed_unitary_pseudogene	0.004416	-1.173
SERTAD4-AS1	lncRNA	0.0293012	-1.174
YBX1	protein_coding	4.79E-25	-1.175
CENPL	protein_coding	0.0006742	-1.176
ST13	protein_coding	3.85E-10	-1.177
USP12	protein_coding	0.0019297	-1.179
PSMD8	protein_coding	7.09E-12	-1.179
PUSL1	protein_coding	0.0420152	-1.180
CTNNAL1	protein_coding	4.11E-14	-1.182
RRAS	protein_coding	0.0041865	-1.183
NBPF8	transcribed_unprocessed_pseudogene	0.0036756	-1.186
SRA1	protein_coding	0.0114442	-1.188
GTF2F2	protein_coding	0.0017078	-1.188
RPS19	protein_coding	2.17E-24	-1.192
SNRPC	protein_coding	1.78E-12	-1.193
XX-FW83563B9.5		0.0335153	-1.195
CYB5R3	protein_coding	4.40E-09	-1.195
SLC39A4	protein_coding	0.0324579	-1.195
PPP1CA	protein_coding	8.58E-09	-1.196
TMEM134	protein_coding	0.0385308	-1.196
RPL34	protein_coding	5.05E-10	-1.197
RPS20	protein_coding	4.29E-21	-1.197
PPM1G	protein_coding	8.01E-15	-1.197
CETN2	protein_coding	0.0029788	-1.198
RPL7P1	processed_pseudogene	0.0235869	-1.198
DCXR	protein_coding	0.005593	-1.199
FEN1	protein_coding	0.0002967	-1.200
RAC2	protein_coding	9.73E-08	-1.201
RPS6	protein_coding	5.37E-23	-1.202
GPX4	protein_coding	1.09E-23	-1.203
SYF2	protein_coding	1.27E-07	-1.203
MAPK3	protein_coding	0.0194553	-1.204
NDUFB10	protein_coding	5.79E-08	-1.204
MMADHC	protein_coding	0.0021077	-1.204
ADI1	protein_coding	2.54E-05	-1.209

CDC45	protein_coding	0.0231281	-1.210
RPL23	protein_coding	1.09E-16	-1.210
EIF3K	protein_coding	1.95E-05	-1.212
PRDX6	protein_coding	1.15E-07	-1.213
NCKIPSD	protein_coding	0.0343769	-1.214
SSBP1	protein_coding	1.71E-06	-1.216
RHOA	protein_coding	1.75E-18	-1.216
DYNC2I2	protein_coding	1.31E-05	-1.220
NUTF2	protein_coding	7.48E-06	-1.220
RPL30	protein_coding	9.99E-17	-1.222
TGM2	protein_coding	1.38E-10	-1.222
EHD2	protein_coding	8.59E-08	-1.223
RPL19	protein_coding	1.06E-34	-1.224
MLST8	protein_coding	5.33E-06	-1.226
RHOC	protein_coding	0.0001333	-1.227
PTMA	protein_coding	1.83E-30	-1.228
VMA21	protein_coding	0.0006447	-1.231
RPL24	protein_coding	9.92E-19	-1.231
SNTA1	protein_coding	0.0011893	-1.231
NOB1	protein_coding	5.42E-05	-1.231
RPLP2	protein_coding	5.21E-17	-1.231
RPS2P5	processed_pseudogene	4.70E-06	-1.233
IDH3G	protein_coding	0.0005559	-1.233
TAGLN2	protein_coding	5.41E-18	-1.234
NDUFAF3	protein_coding	0.0026638	-1.235
SLC25A5	protein_coding	4.13E-15	-1.236
RPL6P27	transcribed_processed_pseudogene	0.0036287	-1.237
MAF1	protein_coding	4.21E-06	-1.238
RPL37A	protein_coding	8.13E-19	-1.238
UBL5	protein_coding	0.0001639	-1.239
MOCS2	protein_coding	4.25E-05	-1.242
MT2A	protein_coding	1.51E-14	-1.243
NAMPTP1	processed_pseudogene	0.0134223	-1.244
DERA	protein_coding	0.003061	-1.245
TNNT1	protein_coding	3.26E-07	-1.247
TPT1	protein_coding	4.53E-13	-1.247
RPS12	protein_coding	1.40E-18	-1.249
ACTG1	protein_coding	1.31E-41	-1.251
NSA2	protein_coding	5.48E-10	-1.252
URM1	protein_coding	0.0011923	-1.253
MAPKAPK3	protein_coding	6.21E-05	-1.253
RPL22P1	processed_pseudogene	0.0137983	-1.254
PTRHD1	protein_coding	0.0042714	-1.255
PRDX2	protein_coding	3.96E-06	-1.255
GUCD1	protein_coding	5.19E-06	-1.256
CAVIN3	protein_coding	2.80E-06	-1.257

EEF1A1P5	processed_pseudogene	7.59E-12	-1.257
PDLIM1	protein_coding	5.77E-06	-1.258
EEF1B2	protein_coding	8.50E-18	-1.259
DAD1	protein_coding	0.0002184	-1.260
KIAA1522	protein_coding	7.91E-05	-1.262
TALDO1	protein_coding	3.11E-16	-1.262
CLNS1A	protein_coding	7.27E-07	-1.263
RPS15A	protein_coding	0.0009761	-1.265
TKT	protein_coding	5.24E-19	-1.265
NACA	protein_coding	1.24E-16	-1.267
EIF5B	protein_coding	6.02E-13	-1.270
CAPG	protein_coding	6.18E-10	-1.271
DHFR	protein_coding	0.0001225	-1.271
NIPSNAP2	protein_coding	0.0034424	-1.272
ATP5MJ	protein_coding	9.99E-08	-1.273
HIRIP3	protein_coding	0.001516	-1.274
RPL39	protein_coding	1.02E-10	-1.276
HNRNPA1	protein_coding	4.56E-21	-1.276
RPS14	protein_coding	1.81E-12	-1.278
NRGN	protein_coding	0.0188994	-1.278
FTL	protein_coding	1.47E-27	-1.279
RPL3	protein_coding	4.72E-17	-1.279
KATNAL1	protein_coding	0.00038	-1.279
RPL7	protein_coding	8.28E-21	-1.280
ZNF880	protein_coding	0.0320123	-1.281
SLC25A43	protein_coding	0.0130841	-1.282
SH3BGRL	protein_coding	0.000613	-1.284
UXT	protein_coding	4.49E-05	-1.285
BNIP3L	protein_coding	8.52E-06	-1.287
KRT7	protein_coding	4.78E-19	-1.287
NT5DC2	protein_coding	1.19E-09	-1.290
DPP3	protein_coding	0.0001218	-1.291
SNHG29	lncRNA	2.41E-10	-1.291
S100A16	protein_coding	3.04E-10	-1.292
RABGGTA	protein_coding	0.016152	-1.292
RPS2P46	processed_pseudogene	1.39E-06	-1.295
EIF3I	protein_coding	7.46E-13	-1.297
EEF1A1	protein_coding	0.0002212	-1.297
FBL	protein_coding	2.82E-07	-1.300
RPL7A	protein_coding	1.95E-26	-1.300
RPS7	protein_coding	2.06E-19	-1.300
EEF1B2P3	processed_pseudogene	0.0215166	-1.300
POLR2I	protein_coding	4.93E-06	-1.301
RPL10P16	processed_pseudogene	0.0411779	-1.301
NQO1	protein_coding	3.40E-09	-1.304
RPS27A	protein_coding	8.17E-18	-1.304

MAFK	protein_coding	0.0097992	-1.304
HIPK2	protein_coding	1.37E-20	-1.304
HMGB1	protein_coding	2.46E-13	-1.308
ABHD4	protein_coding	0.0006782	-1.308
S100A6	protein_coding	2.46E-31	-1.309
RN7SL1	misc_RNA	0.0019793	-1.311
NDUFS5	protein_coding	3.00E-15	-1.312
DUSP23	protein_coding	0.0029274	-1.312
RP11-290D2.6		0.0377533	-1.312
NT5C	protein_coding	0.0009973	-1.313
SMIM4	protein_coding	0.0062018	-1.313
HIGD2A	protein_coding	0.0004652	-1.317
RPS23	protein_coding	1.81E-34	-1.319
ARPC1A	protein_coding	0.0066703	-1.319
RPS27	protein_coding	2.38E-22	-1.320
RNF181	protein_coding	7.01E-05	-1.323
RPL35A	protein_coding	1.41E-15	-1.323
ARHGAP1	protein_coding	0.0002838	-1.327
LAMTOR4	protein_coding	0.0002382	-1.328
RPL35	protein_coding	7.79E-32	-1.329
GTF3A	protein_coding	1.89E-09	-1.329
GPX1	protein_coding	8.87E-13	-1.330
MYL9	protein_coding	3.02E-11	-1.330
C1orf122	protein_coding	0.0039863	-1.331
RPL13A	protein_coding	4.99E-30	-1.332
MRPL9	protein_coding	0.0001419	-1.336
MORC4	protein_coding	3.29E-09	-1.336
RPLP0P6	processed_pseudogene	4.32E-05	-1.338
HYI	protein_coding	6.63E-05	-1.339
TMEM219	protein_coding	0.0034372	-1.339
TOMM7	protein_coding	1.50E-06	-1.339
ZCCHC24	protein_coding	5.27E-05	-1.339
RNASEH2C	protein_coding	0.00016	-1.341
KRT19	protein_coding	7.08E-26	-1.341
CCNI	protein_coding	4.92E-19	-1.342
WTIP	protein_coding	0.0002288	-1.346
SLC9A3R2	protein_coding	2.07E-18	-1.347
PPIA	protein_coding	1.06E-31	-1.349
ECI1	protein_coding	0.0150652	-1.352
RPS28P7	processed_pseudogene	0.0001925	-1.353
RPL5	protein_coding	3.08E-26	-1.355
RPL39P3	processed_pseudogene	0.0077083	-1.356
UCP2	protein_coding	0.0003813	-1.357
RBM3	protein_coding	1.43E-13	-1.357
RPL4	protein_coding	1.11E-32	-1.362
MCM4	protein_coding	5.00E-07	-1.364

C14orf119	protein_coding	0.0051643	-1.366
RPS3AP6	processed_pseudogene	0.0478652	-1.367
FKBP1A	protein_coding	1.35E-09	-1.368
WBP2	protein_coding	2.81E-10	-1.369
MRPL57	protein_coding	0.0060504	-1.370
UBA52	protein_coding	5.62E-18	-1.371
S100A10	protein_coding	1.44E-33	-1.371
PGLS	protein_coding	8.59E-06	-1.372
TUBA4A	protein_coding	0.0002914	-1.373
RPS3A	protein_coding	9.38E-26	-1.376
ERCC1	protein_coding	7.01E-06	-1.378
DEF8	protein_coding	0.0002624	-1.378
RP11-84E17.1		2.02E-08	-1.379
ARRDC2	protein_coding	0.0034248	-1.379
RPLP1	protein_coding	1.30E-23	-1.380
OST4	protein_coding	2.53E-06	-1.380
RPS7P1	processed_pseudogene	9.57E-06	-1.389
BAD	protein_coding	4.55E-07	-1.389
UBE2L6	protein_coding	0.000613	-1.391
RPL27	protein_coding	9.75E-27	-1.394
NDUFV3	protein_coding	5.74E-09	-1.396
IRAK1	protein_coding	4.80E-16	-1.396
RPS11	protein_coding	4.72E-31	-1.397
ATP5F1E	protein_coding	3.94E-16	-1.401
MAGED1	protein_coding	0.0003308	-1.402
NME4	protein_coding	0.0001971	-1.403
AC009245.3		0.0058625	-1.403
NDUFB11	protein_coding	1.81E-06	-1.404
ACOT13	protein_coding	0.0014129	-1.405
RPS4X	protein_coding	1.95E-32	-1.406
RP11-425L10.1		2.22E-10	-1.408
CCND1	protein_coding	1.30E-23	-1.410
KRT80	protein_coding	3.55E-09	-1.411
COPE	protein_coding	5.37E-05	-1.415
RRP8	protein_coding	3.60E-05	-1.417
COX6B1	protein_coding	1.52E-05	-1.417
PABPC1	protein_coding	7.19E-29	-1.418
GUK1	protein_coding	1.18E-15	-1.419
COTL1	protein_coding	9.38E-22	-1.422
RPS26	protein_coding	1.58E-11	-1.423
CDK6	protein_coding	1.83E-08	-1.423
BST2	protein_coding	6.32E-07	-1.424
PSME1	protein_coding	3.55E-09	-1.430
RPSAP15	processed_pseudogene	0.002985	-1.431
IMPDH2	protein_coding	4.73E-15	-1.432
GXYLT1	protein_coding	0.0004253	-1.432

RPS3AP26	processed_pseudogene	0.0029399	-1.433
MPG	protein_coding	0.0004986	-1.433
CDT1	protein_coding	0.0016033	-1.433
HNRNPA1P7	processed_pseudogene	6.19E-07	-1.434
IFITM3	protein_coding	1.74E-08	-1.434
RPL12	protein_coding	5.12E-41	-1.435
NDUFS8	protein_coding	2.26E-08	-1.436
POLR2E	protein_coding	1.36E-12	-1.437
RPL8	protein_coding	1.47E-31	-1.438
RPSAP58	processed_pseudogene	1.59E-06	-1.443
PDLIM7	protein_coding	9.47E-08	-1.444
AKR7A2	protein_coding	1.27E-05	-1.444
AGO1	protein_coding	7.11E-10	-1.452
TSPO	protein_coding	0.0002246	-1.453
CDA	protein_coding	1.94E-05	-1.453
RPS15P4	processed_pseudogene	0.0002762	-1.454
CHP1	protein_coding	5.44E-08	-1.454
CTD-2287O16.1		0.031103	-1.456
MRPL17	protein_coding	4.86E-06	-1.456
RP5-940J5.9		0.0051487	-1.457
MRPL54	protein_coding	0.0101653	-1.457
CCDC34	protein_coding	4.32E-07	-1.459
RABIF	protein_coding	0.0258792	-1.459
RPS16	protein_coding	2.00E-28	-1.460
ZBED5-AS1	lncRNA	5.01E-05	-1.461
VPS25	protein_coding	0.0012309	-1.462
RP4-614O4.13		0.0271334	-1.463
TXN2	protein_coding	0.0004665	-1.463
RNU6-6P	snRNA	9.13E-06	-1.464
HSPB1	protein_coding	5.64E-11	-1.465
HTATSF1	protein_coding	2.12E-16	-1.470
DPM3	protein_coding	0.0081557	-1.470
RPL10	protein_coding	4.41E-25	-1.472
SRM	protein_coding	1.19E-11	-1.474
HNRNPA3	protein_coding	1.07E-16	-1.477
RPS21	protein_coding	4.59E-23	-1.481
PPIAP22	processed_pseudogene	0.003152	-1.482
RP4-706A16.3		7.48E-06	-1.483
RPL37	protein_coding	4.02E-37	-1.486
ANP32A	protein_coding	1.24E-09	-1.488
RPL18	protein_coding	5.48E-24	-1.495
AP1S2	protein_coding	3.58E-07	-1.496
PLP2	protein_coding	1.15E-18	-1.500
CTB-63M22.1		3.69E-05	-1.502
TUBBP1	transcribed_processed_pseudogene	0.0001365	-1.511
RTL8A	protein_coding	0.0001453	-1.512

C17orf67	protein_coding	0.0130633	-1.512
ITPRIPL2	protein_coding	3.35E-05	-1.513
NPM1	protein_coding	9.67E-27	-1.516
RPL11	protein_coding	2.33E-34	-1.516
TRIM52-AS1	lncRNA	0.0338683	-1.518
GPX3	protein_coding	5.27E-07	-1.518
RTL8B	protein_coding	0.005756	-1.519
RPL15P3	processed_pseudogene	0.0061728	-1.520
RAB1B	protein_coding	6.01E-12	-1.521
RP11-466H18.1		3.99E-10	-1.523
RPS15	protein_coding	1.28E-38	-1.523
LGALS1	protein_coding	2.15E-25	-1.526
CLIC3	protein_coding	0.0214507	-1.529
RPL14	protein_coding	8.50E-42	-1.531
RPL21	protein_coding	4.64E-27	-1.531
RPS29	protein_coding	1.14E-06	-1.535
SLC25A23	protein_coding	9.99E-09	-1.541
METTL26	protein_coding	0.0137101	-1.541
FBP1	protein_coding	0.0032657	-1.544
RPS3	protein_coding	1.27E-43	-1.549
RPSA	protein_coding	2.40E-44	-1.552
RPS2	protein_coding	1.61E-40	-1.556
SAP18	protein_coding	3.21E-15	-1.557
LHFPL4	protein_coding	0.0175202	-1.562
CTNNBIP1	protein_coding	2.94E-06	-1.562
S100A2	protein_coding	0.0041854	-1.563
PSMC3IP	protein_coding	7.18E-09	-1.574
RPL41	protein_coding	6.30E-11	-1.578
APRT	protein_coding	0.0006013	-1.582
MYBL2	protein_coding	4.65E-08	-1.584
SCAND1	protein_coding	0.0001194	-1.584
RPL13	protein_coding	7.46E-40	-1.592
MTND2P28	unprocessed_pseudogene	0.0364672	-1.595
RP11-632C17__A.1		2.62E-07	-1.596
RPS5	protein_coding	1.94E-22	-1.601
RACK1	protein_coding	5.75E-48	-1.601
APOL2	protein_coding	0.0003692	-1.605
ISG15	protein_coding	1.98E-06	-1.606
EIF4EBP1	protein_coding	5.07E-11	-1.607
SNORA12	snoRNA	0.0257206	-1.608
S100A11	protein_coding	2.80E-22	-1.611
GAPDH	protein_coding	1.04E-59	-1.613
RPL32	protein_coding	9.05E-42	-1.617
RPL28	protein_coding	2.08E-42	-1.617
RPL27A	protein_coding	1.08E-35	-1.618
MMP24OS	protein_coding	4.82E-18	-1.619

ZNF688	protein_coding	0.0198164	-1.621
CAPNS1	protein_coding	4.82E-21	-1.624
RPL18A	protein_coding	3.95E-48	-1.624
ATP5F1D	protein_coding	1.11E-06	-1.627
FHL3	protein_coding	0.0116427	-1.628
RP11-253E3.3		0.0419733	-1.628
MAP4	protein_coding	1.82E-36	-1.633
RPL9P7	processed_pseudogene	0.0415198	-1.633
ANXA8	protein_coding	0.000459	-1.641
RPL13AP5	processed_pseudogene	1.78E-10	-1.653
GAMT	protein_coding	0.0010685	-1.653
RTL8C	protein_coding	2.58E-08	-1.660
FAM219A	protein_coding	5.47E-05	-1.665
CYP1B1	protein_coding	2.25E-08	-1.668
PLAAT4	protein_coding	9.21E-08	-1.673
KCND1	protein_coding	0.004891	-1.680
CORO1C	protein_coding	1.32E-25	-1.682
NUDT3	protein_coding	1.06E-13	-1.683
SMARCC1	protein_coding	1.35E-26	-1.686
DESI1	protein_coding	0.000886	-1.686
LGALS3	protein_coding	5.03E-14	-1.689
TCTA	protein_coding	0.000124	-1.690
SF3B5	protein_coding	3.20E-05	-1.693
NBPF10	protein_coding	2.57E-06	-1.696
APC	protein_coding	1.42E-06	-1.703
MT-CYB	protein_coding	9.70E-15	-1.703
RPL29	protein_coding	6.83E-44	-1.708
NUAK1	protein_coding	0.0001487	-1.710
CRIP2	protein_coding	0.0190556	-1.711
H1-4	protein_coding	3.61E-31	-1.711
ALAD	protein_coding	2.21E-06	-1.711
RP11-371A22.1		2.06E-11	-1.713
RPL24P4	processed_pseudogene	8.16E-05	-1.719
DDX24	protein_coding	0.0247442	-1.720
KIF3B	protein_coding	1.23E-18	-1.720
GSTP1	protein_coding	1.92E-19	-1.722
MT-ND5	protein_coding	4.68E-31	-1.722
BCYRN1	scRNA	2.63E-07	-1.727
ZNF326	protein_coding	5.66E-09	-1.736
C11orf68	protein_coding	0.0003125	-1.740
PYCARD	protein_coding	0.007504	-1.742
DYNC2I1	protein_coding	5.99E-27	-1.745
RPLP0	protein_coding	7.51E-44	-1.745
SLC24A1	protein_coding	7.68E-07	-1.747
RPL13P12	processed_pseudogene	0.0097127	-1.751
TK1	protein_coding	6.15E-11	-1.753

ZNF358	protein_coding	8.00E-05	-1.758
RPS28	protein_coding	2.26E-17	-1.771
HMGA1	protein_coding	8.86E-69	-1.785
RP11-543P15.1		0.0018943	-1.786
OCEL1	protein_coding	0.002525	-1.788
SH3BGRL3	protein_coding	1.51E-34	-1.796
MTCO1P12	unprocessed_pseudogene	0.0083614	-1.800
PTPN11	protein_coding	3.36E-34	-1.804
MT-ATP8	protein_coding	9.44E-05	-1.810
BRK1	protein_coding	2.04E-07	-1.811
TXNRD3	protein_coding	0.0009131	-1.816
NIPSNAP3A	protein_coding	0.0021143	-1.861
ATG4D	protein_coding	0.004256	-1.868
UNG	protein_coding	0.0003602	-1.872
ASPHD1	protein_coding	0.0030674	-1.877
ALKBH7	protein_coding	5.33E-05	-1.890
SET	protein_coding	4.37E-66	-1.910
H3C13	protein_coding	0.0478123	-1.911
SH3RF2	protein_coding	2.36E-15	-1.913
RP1-278E11.3		0.0080924	-1.920
MAPKBP1	protein_coding	3.74E-08	-1.922
PINK1	protein_coding	5.41E-17	-1.927
GAB2	protein_coding	6.91E-13	-1.936
CHAF1A	protein_coding	8.07E-32	-1.940
TSNARE1	protein_coding	0.000118	-1.946
HCFC1R1	protein_coding	4.30E-09	-1.946
NSRP1	protein_coding	1.57E-34	-1.947
RPL18AP3	processed_pseudogene	2.32E-07	-1.949
PALLD	protein_coding	2.61E-33	-1.990
TCEAL4	protein_coding	4.18E-31	-1.997
ESF1	protein_coding	6.07E-26	-1.997
H2BC5	protein_coding	1.40E-24	-2.002
H2BC12	protein_coding	2.83E-38	-2.002
PPDPF	protein_coding	8.43E-24	-2.003
RNY1	misc_RNA	5.15E-25	-2.013
RNY3	misc_RNA	1.92E-25	-2.017
RN7SL5P	misc_RNA	0.0140611	-2.018
H4C8	protein_coding	6.10E-05	-2.022
APOO	protein_coding	0.0008187	-2.023
H4C2	protein_coding	1.23E-17	-2.038
NAP1L1	protein_coding	8.97E-53	-2.043
MT-ATP6	protein_coding	2.26E-13	-2.058
MT-CO3	protein_coding	2.34E-22	-2.078
AC004057.1	transcribed_processed_pseudogene	0.0009077	-2.090
RAI14	protein_coding	7.87E-45	-2.096
RP5-1182A14.7		0.0390269	-2.110

DYNC1LI2	protein_coding	1.09E-21	-2.114
MAP4K4	protein_coding	3.04E-90	-2.139
KCNK6	protein_coding	1.36E-08	-2.144
H1-2	protein_coding	3.17E-56	-2.146
CDC42BPG	protein_coding	1.11E-15	-2.148
HNRNPH3	protein_coding	1.02E-51	-2.162
MT-RNR1	Mt_rRNA	3.78E-07	-2.177
MRPL23	protein_coding	0.0041176	-2.181
ADIRF	protein_coding	1.16E-05	-2.202
H2AC6	protein_coding	2.19E-22	-2.203
H2AC14	protein_coding	8.88E-17	-2.220
WWTR1	protein_coding	1.86E-33	-2.224
FBXL19-AS1	lncRNA	9.23E-07	-2.228
H2AC20	protein_coding	4.54E-18	-2.241
H2BC15	protein_coding	2.25E-12	-2.273
H2BC4	protein_coding	1.32E-48	-2.312
ZBTB47	protein_coding	2.27E-17	-2.321
H4C12	protein_coding	0.0013607	-2.335
MT-ND2	protein_coding	3.35E-41	-2.337
VTRNA1-2	misc_RNA	0.0386875	-2.364
H4-16	protein_coding	0.0016278	-2.371
RP11-644F5.11		0.000583	-2.378
ABLIM3	protein_coding	8.33E-19	-2.382
MT-ND1	protein_coding	2.53E-38	-2.385
PLEKHA4	protein_coding	1.49E-39	-2.387
H2BC18	protein_coding	2.44E-23	-2.395
TCEAL3	protein_coding	5.94E-27	-2.398
MTATP6P1	unprocessed_pseudogene	1.12E-08	-2.399
MT-CO1	protein_coding	2.11E-72	-2.400
SYNJ2BP	protein_coding	7.61E-10	-2.449
MT-ND3	protein_coding	0.0001231	-2.476
H2AC13	protein_coding	6.47E-10	-2.490
MT-ND4	protein_coding	1.09E-53	-2.502
ANXA8L1	protein_coding	0.000687	-2.504
MT-RNR2	Mt_rRNA	7.94E-58	-2.506
H2AC12	protein_coding	7.04E-22	-2.530
CENPB	protein_coding	7.99E-29	-2.540
PKP4	protein_coding	2.02E-68	-2.543
MT-ND4L	protein_coding	2.01E-05	-2.546
H4C1	protein_coding	0.0003206	-2.548
NBPF14	protein_coding	1.20E-29	-2.562
RPS27AP16	transcribed_processed_pseudogene	0.0187873	-2.565
PFN1P1	processed_pseudogene	0.000611	-2.569
LPAR1	protein_coding	6.24E-29	-2.574
H2BC11	protein_coding	6.05E-24	-2.574
GDF11	protein_coding	1.62E-25	-2.584

MT-TW	Mt_tRNA	0.0013838	-2.584
MT-CO2	protein_coding	8.76E-79	-2.588
H3C1	protein_coding	0.0001014	-2.603
TTLL11	protein_coding	3.77E-15	-2.603
PURA	protein_coding	1.92E-32	-2.604
H4C3	protein_coding	5.26E-18	-2.615
H2BC21	protein_coding	1.21E-11	-2.617
H2AC15	protein_coding	1.03E-10	-2.641
SAMD4A	protein_coding	2.56E-32	-2.647
SLC25A6	protein_coding	2.91E-05	-2.705
LINC00638	lncRNA	0.0011333	-2.710
YJEFN3	protein_coding	4.46E-23	-2.756
SNX8	protein_coding	5.48E-39	-2.778
H2AC4	protein_coding	8.40E-11	-2.788
RNY4	misc_RNA	5.95E-09	-2.793
H2AC11	protein_coding	3.26E-55	-2.808
H2BC13	protein_coding	1.68E-06	-2.810
H2BC14	protein_coding	9.16E-05	-2.811
H3C2	protein_coding	3.16E-55	-2.827
MTURN	protein_coding	1.76E-28	-2.866
EIF3CL	protein_coding	0.0126975	-2.872
H2BC3	protein_coding	2.09E-21	-2.903
IFI27	protein_coding	3.53E-05	-2.923
UACA	protein_coding	1.25E-89	-2.946
H3C3	protein_coding	3.66E-52	-2.981
ZEB1	protein_coding	1.96E-111	-2.988
H2AC21	protein_coding	2.09E-22	-2.995
PITPNM3	protein_coding	2.59E-78	-3.002
LACC1	protein_coding	1.39E-16	-3.040
ARRDC4	protein_coding	2.04E-08	-3.076
H2BC17	protein_coding	4.01E-46	-3.103
H3C10	protein_coding	5.04E-21	-3.221
ANP32B	protein_coding	5.72E-117	-3.224
TP53INP2	protein_coding	1.82E-39	-3.241
IGIP	protein_coding	5.91E-09	-3.298
BCL2L2	protein_coding	1.19E-33	-3.328
NUDT16	protein_coding	1.84E-55	-3.431
SH3PXD2A	protein_coding	2.76E-96	-3.433
CRTAP	protein_coding	9.71E-143	-3.441
H4C11	protein_coding	2.83E-05	-3.492
PGPEP1	protein_coding	7.71E-73	-3.545
ZSWIM9	protein_coding	5.21E-47	-3.631
C22orf46	transcribed_unitary_pseudogene	3.46E-167	-3.653
CTD-3099C6.13		8.58E-120	-3.724
FBXW4	protein_coding	2.42E-50	-3.742
RAB13	protein_coding	2.47E-96	-3.892

RASSF3	protein_coding	9.52E-168	-3.969
KIF1C	protein_coding	0	-4.046
TRAK2	protein_coding	5.02E-249	-4.174
NET1	protein_coding	0	-4.432
RP11-603J24.7	processed_pseudogene	3.44E-95	-4.460

Supplementary Table S5. Full list of differentially expressed small RNAs (i.e. miRNAs, snoRNAs, piRNAs and tRNA-derived fragments). The table shows gene name, gene type, amount of differential expression in terms of logarithm of the base 2 of the Fold change (log2FC), adjusted P-value.

Name	Official Name	adjusted p-value	log2(FoldChange)
HBII-419	SNORD98	0.001945745	4.031
HBI-115	SNORA47	2.34E-11	3.551
HBII-239	SNORD71	2.83E-08	3.250
U106	SNORD12C	0.003250867	3.134
U28	SNORD28	2.34E-11	3.066
piR-hsa-23566	piR-hsa-23566	1.54E-05	3.010
U37	SNORD37	0.000450443	2.870
HBII-295	SNORD90	0.004611853	2.715
U18B	SNORD18B	0.00603962	2.710
SNORD127	SNORD127	0.012604674	2.672
E2	SNORA62	2.61E-10	2.555
HBII-108B	SNORD19B	0.029368823	2.480
U83A	SNORD83A	3.60E-08	2.345
piR-hsa-1834	piR-hsa-1834	0.011518158	2.340
U46	SNORD46	0.000138538	2.333
piR-hsa-26039	piR-hsa-26039	1.01E-05	2.325
U34	SNORD34	0.033602158	2.274
U78	SNORD78	1.64E-07	2.203
SNORD123	SNORD123	0.000140412	2.179
U49A	SNORD49A	1.54E-05	2.154
U38A	SNORD38A	0.001804525	2.104
U44	SNORD44	1.01E-07	2.026
U42B	SNORD42B	0.011518158	2.009
snR38C	SNORD1C	0.004030615	1.997
ACA55	SNORA55	0.015115556	1.992
piR-hsa-963	piR-hsa-963	0.011808293	1.988
HBII-99B	SNORD12B	0.044876368	1.965
U47	SNORD47	0.000184785	1.947
hsa-let-7a-5p	hsa-let-7a-5p	2.68E-05	1.894
U14B	SNORD14B	0.037426583	1.892
U105B	SNORD105B	0.012024773	1.875
snR38B	SNORD1B	0.000208326	1.857
HBII-135	SNORD65	0.01760122	1.842
U36B	SNORD36B	0.012024773	1.808
hsa-miR-17-3p	hsa-miR-17-3p	0.031526296	1.801

U75	SNORD75	0.031526296	1.786
tRFdb-5026a-435	tRFdb-5026a-435	0.000551326	1.759
U27	SNORD27	0.030951235	1.750
hsa-let-7f-5p_1	hsa-let-7f-5p_1	1.79E-05	1.692
hsa-let-7f-5p	hsa-let-7f-5p	0.000489777	1.664
piR-hsa-11360	piR-hsa-11360	0.000501853	1.663
U94	SNORD94	0.00014761	1.661
ACA43	SNORA43	0.003158675	1.602
U3	SNORD3@	0.027085968	1.594
piR-hsa-24000	piR-hsa-24000	0.026917937	1.554
HBII-99	SNORD12	0.01828989	1.552
U41	SNORD41	0.003655502	1.547
piR-hsa-20757	piR-hsa-20757	0.021170337	1.535
U18A	SNORD18A	0.02028284	1.533
mgU2-19/30	SCARNA9	0.046725168	1.525
hsa-miR-29a-3p	hsa-miR-29a-3p	3.42E-05	1.513
hsa-let-7a-5p_2	hsa-let-7a-5p	0.003303137	1.496
U45A	SNORD45A	0.040556392	1.483
piR-hsa-1338	piR-hsa-1338	0.003963276	1.454
mgh28S-2411	SNORD6	0.002968098	1.448
U74	SNORD74	0.001758718	1.443
hsa-miR-126-3p	hsa-miR-126-3p	2.76E-07	1.442
U38B	SNORD38B	0.023662453	1.409
piR-hsa-11361	piR-hsa-11361	0.001860243	1.395
hsa-miR-151a-5p	hsa-miR-151a-5p	0.044876368	1.375
U29	SNORD29	0.00816336	1.326
hsa-miR-30b-5p	hsa-miR-30b-5p	6.17E-06	1.317
U22	SNORD22	0.004622223	1.299
U14A	SNORD14A	0.007972601	1.280
hsa-miR-150-5p	hsa-miR-150-5p	0.036637167	1.274
HBII-210	SNORD69	0.016859984	1.265
hsa-miR-30c-5p_1	hsa-miR-30c-5p	0.004771216	1.225
U73a	SNORD73A	0.023973	1.201
U102	SNORD102	0.006202267	1.201
U82	SNORD82	0.041143864	1.187
hsa-let-7e-5p	hsa-let-7e-5p	0.005438103	1.184
hsa-let-7b-5p	hsa-let-7b-5p	0.041626029	1.182
U109	SCARNA18	0.029368823	1.110
U57	SNORD57	4.11E-05	1.109
hsa-miR-30c-2-3p	hsa-miR-30c-2-3p	0.016772502	1.065
U83B	SNORD83B	0.023973	1.047
U65	SNORA65	0.045915112	1.042
hsa-miR-30a-5p	hsa-miR-30a-5p	0.00092912	1.035
tRFdb-5020a-338	tRFdb-5020a-338	0.000716457	1.023
hsa-miR-29c-3p	hsa-miR-29c-3p	0.002198833	1.009
hsa-miR-92b-3p	hsa-miR-92b-3p	0.019620441	-1.038

U59B	SNORD59B	0.024351039	-1.078
hsa-miR-671-5p	hsa-miR-671-5p	0.027319547	-1.130
tRFdb-5032a-504	tRFdb-5032a-504	6.17E-06	-1.144
hsa-let-7d-3p	hsa-let-7d-3p	0.003492552	-1.168
SNORA38B	SNORA38B	0.044755394	-1.304
ACA21	SNORA21	0.007699912	-1.342
U71a	SNORA71A	0.02028284	-1.380
hsa-miR-942-5p	hsa-miR-942-5p	0.016679904	-1.421
hsa-miR-125b-5p_1	hsa-miR-125b-5p	0.02651708	-1.453
hsa-miR-450b-5p	hsa-miR-450b-5p	0.016772502	-1.579
5P_tRNA-Ser-TGA-1-1	5P_tRNA-Ser-TGA-1-1	0.032083442	-1.587
U80	SNORD80	0.005438103	-1.617
hsa-miR-379-5p	hsa-miR-379-5p	0.034147577	-1.663
HBII-429	SNORD100	7.60E-05	-1.673
hsa-miR-451a	hsa-miR-451a	0.012552502	-1.745
piR-hsa-2491	piR-hsa-2491	0.037882925	-1.818
hsa-miR-148a-3p	hsa-miR-148a-3p	0.02973412	-1.852
hsa-miR-335-3p	hsa-miR-335-3p	0.031627236	-1.898
piR-hsa-32299	piR-hsa-32299	0.031526296	-1.971
hsa-miR-125a-5p	hsa-miR-125a-5p	1.11E-05	-1.993
hsa-miR-671-3p	hsa-miR-671-3p	0.013442311	-2.012
piR-hsa-7695	piR-hsa-7695	0.005360417	-2.031
hsa-miR-1296-5p	hsa-miR-1296-5p	0.00266175	-2.050
hsa-miR-497-5p	hsa-miR-497-5p	0.036515117	-2.124
ACA62	SNORA76	1.79E-05	-2.151
tRFdb-3004a-618	tRFdb-3004a-618	0.011444528	-2.165
hsa-miR-576-5p	hsa-miR-576-5p	0.006531813	-2.175
hsa-miR-486-5p	hsa-miR-486-5p	0.000385341	-2.187
piR-hsa-20230	piR-hsa-20230	0.031526296	-2.212
piR-hsa-30636	piR-hsa-30636	0.013106285	-2.226
hsa-miR-144-5p	hsa-miR-144-5p	1.32E-06	-2.263
hsa-miR-197-3p	hsa-miR-197-3p	1.79E-05	-2.297
hsa-miR-494-3p	hsa-miR-494-3p	0.016434456	-2.341
hsa-miR-874-3p	hsa-miR-874-3p	0.044787273	-2.401
tRFdb-5011a-129	tRFdb-5011a-129	0.049313626	-2.415
piR-hsa-1593	piR-hsa-1593	0.048719427	-2.417
hsa-miR-381-3p	hsa-miR-381-3p	0.041143864	-2.432
hsa-miR-127-3p	hsa-miR-127-3p	0.000170038	-2.436
piR-hsa-426	piR-hsa-426	0.039076393	-2.438
piR-hsa-13633	piR-hsa-13633	0.035680541	-2.469
hsa-miR-1307-3p	hsa-miR-1307-3p	3.35E-12	-2.516
U8	SNORD118	0.023973	-2.589
hsa-miR-3615	hsa-miR-3615	0.002737401	-2.636
hsa-miR-122-5p	hsa-miR-122-5p	0.021222107	-2.698
U89	SCARNA12	0.023973	-2.794
ts-103	ts-103	0.003655502	-2.852

hsa-miR-432-5p	hsa-miR-432-5p	0.001945745	-2.888
hsa-miR-144-3p	hsa-miR-144-3p	0.00355425	-2.896
hsa-miR-328-3p	hsa-miR-328-3p	1.67E-07	-2.916
piR-hsa-30734	piR-hsa-30734	0.008418354	-2.927
ts-47	ts-47	0.009467337	-2.973
piR-hsa-23679	piR-hsa-23679	0.000450443	-3.007
hsa-miR-369-3p	hsa-miR-369-3p	0.000714419	-3.037
5P_tRNA-Leu-TAA-1-1	5P_tRNA-Leu-TAA-1-1	0.00417747	-3.096
piR-hsa-22021	piR-hsa-22021	0.002780882	-3.132
piR-hsa-23317	piR-hsa-23317	0.012024773	-3.149
ACA8	SNORA8	0.01828989	-3.238
hTR	TERC	0.00790365	-3.260
piR-hsa-27728	piR-hsa-27728	0.012629649	-3.414
hsa-miR-136-3p	hsa-miR-136-3p	0.004793526	-3.504
piR-hsa-7201	piR-hsa-7201	0.000406054	-3.541
piR-hsa-21000	piR-hsa-21000	0.002517521	-3.577
piR-hsa-27007	piR-hsa-27007	0.012863505	-3.609
hsa-miR-3605-3p	hsa-miR-3605-3p	0.000140286	-3.624
piR-hsa-28634	piR-hsa-28634	0.00346579	-3.663
piR-hsa-14870	piR-hsa-14870	0.00180319	-3.716
hsa-miR-193a-5p	hsa-miR-193a-5p	0.000102838	-3.754
piR-hsa-9104	piR-hsa-9104	4.68E-06	-3.822
piR-hsa-19996	piR-hsa-19996	0.002290491	-3.902
piR-hsa-27282	piR-hsa-27282	0.001235256	-3.956
hsa-miR-214-3p	hsa-miR-214-3p	0.001705624	-3.956
piR-hsa-26925	piR-hsa-26925	0.000162689	-3.992
piR-hsa-11256	piR-hsa-11256	6.17E-06	-4.008
mgU2-25/61	SCARNA2	0.000170038	-4.024
piR-hsa-23387	piR-hsa-23387	0.005338547	-4.176
piR-hsa-23919	piR-hsa-23919	0.000296953	-4.306
piR-hsa-28212	piR-hsa-28212	0.008025178	-4.316
piR-hsa-23670	piR-hsa-23670	0.001523838	-4.387
piR-hsa-7193	piR-hsa-7193	0.00266175	-4.456
piR-hsa-27513	piR-hsa-27513	0.000726201	-4.528
tRFdb-3004a-617	tRFdb-3004a-617	4.84E-05	-4.730
hsa-miR-155-5p	hsa-miR-155-5p	7.75E-06	-4.824
ts-112	ts-112	1.59E-05	-4.833
piR-hsa-7116	piR-hsa-7116	7.12E-06	-4.912
piR-hsa-28382	piR-hsa-28382	0.000118001	-5.091
piR-hsa-17793	piR-hsa-17793	0.00086163	-5.320
piR-hsa-2467	piR-hsa-2467	3.59E-05	-5.486
piR-hsa-28205	piR-hsa-28205	0.000108844	-5.755
5P_tRNA-His-GTG-1-8	5P_tRNA-His-GTG-1-8	4.44E-05	-6.275
piR-hsa-28478	piR-hsa-28478	0.00050761	-6.291
piR-hsa-5939	piR-hsa-5939	3.80E-07	-6.562
piR-hsa-25046	piR-hsa-25046	7.45E-08	-6.671

ts-44	ts-44	1.02E-05	-7.335
piR-hsa-12789	piR-hsa-12789	0.00014761	-7.417
hsa-miR-6087	hsa-miR-6087	2.68E-05	-7.987
hsa-miR-1306-5p	hsa-miR-1306-5p	5.79E-16	-8.193
hsa-miR-196b-5p	hsa-miR-196b-5p	2.07E-05	-8.454
piR-hsa-9105	piR-hsa-9105	1.64E-07	-9.148
piR-hsa-12275	piR-hsa-12275	2.15E-07	-10.756

Supplementary Table S6. Enrichment Pathway analysis of up-regulated genes.

GO BP	adj. P-value
extracellular structure organization (GO:0043062)	7.46E-13
external encapsulating structure organization (GO:0045229)	1.88E-12
extracellular matrix organization (GO:0030198)	1.04E-10
homophilic cell adhesion via plasma membrane adhesion molecules (GO:0007156)	8.53E-10
axon guidance (GO:0007411)	9.00E-10
axonogenesis (GO:0007409)	1.30E-08
lipid translocation (GO:0034204)	3.20E-08
cell-cell adhesion via plasma-membrane adhesion molecules (GO:0098742)	9.57E-08
phospholipid translocation (GO:0045332)	0.000004512
collagen fibril organization (GO:0030199)	0.000004512
synapse organization (GO:0050808)	0.00001251
transport across blood-brain barrier (GO:0150104)	0.00002676
nervous system development (GO:0007399)	0.0000271
regulation of dendrite development (GO:0050773)	0.00002748
vascular transport (GO:0010232)	0.00005104
phospholipid transport (GO:0015914)	0.00005137
neuron migration (GO:0001764)	0.00005602
regulation of cell migration (GO:0030334)	0.00005987
calcium ion transport (GO:0006816)	0.00009035
neuron projection morphogenesis (GO:0048812)	0.0001148
negative regulation of cell adhesion (GO:0007162)	0.0001295
negative regulation of chemotaxis (GO:0050922)	0.0002193
regulation of NMDA receptor activity (GO:2000310)	0.0002193
aorta development (GO:0035904)	0.0002911
negative chemotaxis (GO:0050919)	0.0003171
neuronal action potential (GO:0019228)	0.0005497
membrane depolarization during action potential (GO:0086010)	0.0005497
modulation of chemical synaptic transmission (GO:0050804)	0.0006166
semaphorin-plexin signaling pathway (GO:0071526)	0.0006344
action potential (GO:0001508)	0.0007905
membrane depolarization (GO:0051899)	0.001068
regulation of axon extension involved in axon guidance (GO:0048841)	0.0013
cilium movement (GO:0003341)	0.001352
lipid phosphorylation (GO:0046834)	0.001422
positive regulation of cell migration (GO:0030335)	0.001682
membrane depolarization during cardiac muscle cell action potential (GO:0086012)	0.001764
cation transport (GO:0006812)	0.00179
chloride transport (GO:0006821)	0.002151
inorganic anion transmembrane transport (GO:0098661)	0.002167
chloride transmembrane transport (GO:1902476)	0.002584
regulation of cation channel activity (GO:2001257)	0.002584
regulation of endothelial cell migration (GO:0010594)	0.002584
central nervous system development (GO:0007417)	0.002584
negative regulation of axon extension (GO:0030517)	0.002962
sodium ion transport (GO:0006814)	0.002962
positive regulation of cell motility (GO:2000147)	0.003101
cilium-dependent cell motility (GO:0060285)	0.00399
glutamate receptor signaling pathway (GO:0007215)	0.004311

synapse assembly (GO:0007416)	0.004325
eye photoreceptor cell differentiation (GO:0001754)	0.00529
aminophospholipid transport (GO:0015917)	0.005396
positive regulation of axonogenesis (GO:0050772)	0.005396
neuromuscular junction development (GO:0007528)	0.005505
negative regulation of axon extension involved in axon guidance (GO:0048843)	0.005978
xenobiotic transport (GO:0042908)	0.005978
protein O-linked glycosylation (GO:0006493)	0.006804
cAMP-mediated signaling (GO:0019933)	0.007744
semaphorin-plexin signaling pathway involved in neuron projection guidance (GO:1902285)	0.008997
regulation of synaptic transmission, glutamatergic (GO:0051966)	0.01081
positive regulation of cation transmembrane transport (GO:1904064)	0.01149
cyclic-nucleotide-mediated signaling (GO:0019935)	0.0116
peptidyl-tyrosine phosphorylation (GO:0018108)	0.0116
cellular response to purine-containing compound (GO:0071415)	0.01178
negative regulation of cAMP-mediated signaling (GO:0043951)	0.01178
regulation of neurotransmitter receptor activity (GO:0099601)	0.01194
regulation of kinase activity (GO:0043549)	0.01194
inorganic cation transmembrane transport (GO:0098662)	0.01194
cellular response to caffeine (GO:0071313)	0.01227
regulation of cilium beat frequency (GO:0003356)	0.01227
response to caffeine (GO:0031000)	0.01227
SA node cell action potential (GO:0086015)	0.01227
enzyme linked receptor protein signaling pathway (GO:0007167)	0.01227
regulation of cardiac muscle cell contraction (GO:0086004)	0.01286
chemical synaptic transmission (GO:0007268)	0.01286
phosphatidylinositol-mediated signaling (GO:0048015)	0.01376
positive regulation of phosphatidylinositol 3-kinase signaling (GO:0014068)	0.01376
diacylglycerol metabolic process (GO:0046339)	0.01558
brain development (GO:0007420)	0.0163
positive regulation of synapse assembly (GO:0051965)	0.01675
muscle cell development (GO:0055001)	0.01675
sensory perception of sound (GO:0007605)	0.01684
anterograde trans-synaptic signaling (GO:0098916)	0.01775
insulin-like growth factor receptor signaling pathway (GO:0048009)	0.01801
positive regulation of potassium ion transmembrane transport (GO:1901381)	0.01801
supramolecular fiber organization (GO:0097435)	0.01925
regulation of phosphatidylinositol 3-kinase signaling (GO:0014066)	0.02183
calcium ion transmembrane transport (GO:0070588)	0.02273
membrane depolarization during SA node cell action potential (GO:0086046)	0.02461
protein localization to axon (GO:0099612)	0.02461
SA node cell to atrial cardiac muscle cell signaling (GO:0086018)	0.02461
positive regulation of cell junction assembly (GO:1901890)	0.02461
metal ion transport (GO:0030001)	0.02488
sensory perception of mechanical stimulus (GO:0050954)	0.02488
cellular response to amyloid-beta (GO:1904646)	0.02503
muscle cell differentiation (GO:0042692)	0.02503
artery morphogenesis (GO:0048844)	0.02581
cardiac muscle cell action potential (GO:0086001)	0.02581
cell morphogenesis involved in neuron differentiation (GO:0048667)	0.0261
basement membrane organization (GO:0071711)	0.02629
dendrite self-avoidance (GO:0070593)	0.02629
glomerular visceral epithelial cell differentiation (GO:0072112)	0.02677
negative regulation of vascular permeability (GO:0043116)	0.02677

protein localization to synapse (GO:0035418)	0.02677
import into cell (GO:0098657)	0.02677
regulation of neuron projection development (GO:0010975)	0.02985
positive regulation of cell projection organization (GO:0031346)	0.03177
endothelial cell development (GO:0001885)	0.0319
positive regulation of ion transmembrane transporter activity (GO:0032414)	0.0319
calcium ion transmembrane import into cytosol (GO:0097553)	0.03214
heterophilic cell-cell adhesion via plasma membrane cell adhesion molecules (GO:0007157)	0.03214
positive regulation of cardiac muscle tissue growth (GO:0055023)	0.03605
pulmonary valve morphogenesis (GO:0003184)	0.03605
regulation of cellular response to growth factor stimulus (GO:0090287)	0.03605
neuronal ion channel clustering (GO:0045161)	0.03759
semaphorin-plexin signaling pathway involved in axon guidance (GO:1902287)	0.03759
endodermal cell differentiation (GO:0035987)	0.03759
neurotransmitter transport (GO:0006836)	0.03759
positive regulation of synaptic transmission (GO:0050806)	0.03759
positive regulation of sodium ion transport (GO:0010765)	0.03934
regulation of cardiac conduction (GO:1903779)	0.0397
actin-myosin filament sliding (GO:0033275)	0.04137
muscle filament sliding (GO:0030049)	0.04137
positive regulation of potassium ion transport (GO:0043268)	0.04137
cell-matrix adhesion (GO:0007160)	0.04137
sodium ion transmembrane transport (GO:0035725)	0.04204
positive regulation of ion transport (GO:0043270)	0.04533
peptidyl-tyrosine modification (GO:0018212)	0.04533
cell migration involved in sprouting angiogenesis (GO:0002042)	0.0466
protein O-linked mannosylation (GO:0035269)	0.0466
pulmonary valve development (GO:0003177)	0.0466
cellular response to organic cyclic compound (GO:0071407)	0.0466
positive regulation of cell communication (GO:0010647)	0.04698
receptor clustering (GO:0043113)	0.04698
potassium ion transport (GO:0006813)	0.04698
regulation of heart rate by cardiac conduction (GO:0086091)	0.04805
skeletal system development (GO:0001501)	0.04888
**GO MF**	**adj. P-value**
glycerophospholipid flippase activity (GO:0140333)	0.000006188
voltage-gated calcium channel activity (GO:0005245)	0.00007446
transmembrane receptor protein tyrosine kinase activity (GO:0004714)	0.00007446
transmembrane receptor protein kinase activity (GO:0019199)	0.0002612
ABC-type xenobiotic transporter activity (GO:0008559)	0.0002666
transmembrane receptor protein phosphatase activity (GO:0019198)	0.0009198
transmembrane receptor protein tyrosine phosphatase activity (GO:0005001)	0.0009198
voltage-gated cation channel activity (GO:0022843)	0.00128
chemorepellent activity (GO:0045499)	0.001464
diacylglycerol kinase activity (GO:0004143)	0.00213
voltage-gated sodium channel activity (GO:0005248)	0.00213
calcium channel activity (GO:0005262)	0.00213
glutamate receptor activity (GO:0008066)	0.002782
semaphorin receptor binding (GO:0030215)	0.002782
chloride channel activity (GO:0005254)	0.003192
phosphatidylethanolamine flippase activity (GO:0090555)	0.003376
ion channel activity (GO:0005216)	0.004711

ionotropic glutamate receptor activity (GO:0004970)	0.005837
cation channel activity (GO:0005261)	0.005837
high voltage-gated calcium channel activity (GO:0008331)	0.007022
voltage-gated chloride channel activity (GO:0005247)	0.007022
aminophospholipid flippase activity (GO:0015247)	0.007858
sodium channel activity (GO:0005272)	0.01088
semaphorin receptor activity (GO:0017154)	0.01233
transmitter-gated ion channel activity (GO:0022824)	0.01406
L-leucine transmembrane transporter activity (GO:0015190)	0.01461
phosphatidylcholine flippase activity (GO:0140345)	0.01461
phosphatidylserine flippase activity (GO:0140346)	0.01461
voltage-gated calcium channel activity involved in cardiac muscle cell action potential	0.01461
PDZ domain binding (GO:0030165)	0.01461
protein tyrosine kinase activity (GO:0004713)	0.01567
3',5'-cyclic-nucleotide phosphodiesterase activity (GO:0004114)	0.01987
motor activity (GO:0003774)	0.02283
calcium ion binding (GO:0005509)	0.02283
GABA-gated chloride ion channel activity (GO:0022851)	0.02617
amyloid-beta binding (GO:0001540)	0.02643
actin binding (GO:0003779)	0.02696
anion channel activity (GO:0005253)	0.02799
microtubule motor activity (GO:0003777)	0.02888
ligand-gated anion channel activity (GO:0099095)	0.02898
phosphatidylcholine transporter activity (GO:0008525)	0.02898
cyclic-nucleotide phosphodiesterase activity (GO:0004112)	0.03023
ankyrin binding (GO:0030506)	0.03989
ligand-gated channel activity (GO:0022834)	0.04796
insulin-like growth factor binding (GO:0005520)	0.04949
**GO CC**	**adj. P-value**
integral component of plasma membrane (GO:0005887)	4.39E-10
axon (GO:0030424)	1.83E-09
neuron projection (GO:0043005)	1.83E-09
collagen-containing extracellular matrix (GO:0062023)	4.62E-07
calcium channel complex (GO:0034704)	0.00007643
dendrite (GO:0030425)	0.00008868
sodium channel complex (GO:0034706)	0.0001061
endoplasmic reticulum lumen (GO:0005788)	0.0003574
voltage-gated sodium channel complex (GO:0001518)	0.0005942
cell-cell junction (GO:0005911)	0.0006117
synaptic membrane (GO:0097060)	0.0008103
voltage-gated calcium channel complex (GO:0005891)	0.0009297
basement membrane (GO:0005604)	0.001221
adherens junction (GO:0005912)	0.001717
dendrite membrane (GO:0032590)	0.001985
sarcoplasmic reticulum (GO:0016529)	0.002578
cytoskeleton of presynaptic active zone (GO:0048788)	0.004377
catenin complex (GO:0016342)	0.01488
intercalated disc (GO:0014704)	0.01488
cell projection membrane (GO:0031253)	0.01771
potassium channel complex (GO:0034705)	0.01951
motile cilium (GO:0031514)	0.02081
smooth endoplasmic reticulum (GO:0005790)	0.0321

muscle myosin complex (GO:0005859)	0.03853
caveola (GO:0005901)	0.03853
cation channel complex (GO:0034703)	0.03853
platelet dense tubular network (GO:0031094)	0.04097
endocytic vesicle membrane (GO:0030666)	0.04097
postsynaptic density (GO:0014069)	0.04685
ionotropic glutamate receptor complex (GO:0008328)	0.04894
actin cytoskeleton (GO:0015629)	0.04894
**BioPlanet 2019**	**adj. P-value**
Axon guidance	1.10E-11
Interaction between L1-type proteins and ankyrins	1.18E-08
ECM-receptor interaction	3.30E-08
ABC transporters	4.59E-08
Collagen biosynthesis and modifying enzymes	4.59E-08
Developmental biology	4.59E-08
L1CAM interactions	7.88E-08
NCAM1 interactions	3.40E-07
NCAM signaling for neurite out-growth	3.40E-07
Arrhythmogenic right ventricular cardiomyopathy (ARVC)	0.000001494
Beta-1 integrin cell surface interactions	0.00000167
Transmission across chemical synapses	0.00000261
Ion channel transport	0.000006253
Transmembrane transport of small molecules	0.000006253
Extracellular matrix organization	0.000008971
Ion transport by P-type ATPases	0.00001108
Neuronal system	0.0000176
Syndecan 1 pathway	0.00009672
PIP2 hydrolysis	0.0001219
Dilated cardiomyopathy	0.0001219
Adrenergic pathway	0.0004167
Nitric oxide stimulation of guanylate cyclase	0.0004667
Integrins in angiogenesis	0.0006068
Focal adhesion	0.001279
Integrin cell surface interactions	0.001705
Hemostasis pathway	0.001982
Beta-3 integrin cell surface interactions	0.002683
Platelet homeostasis	0.002683
G alpha s pathway	0.007773
GABA A and B receptor activation	0.008237
Calcium signaling pathway	0.01122
Plexin D1 signaling	0.01459
Neurophilin interactions with VEGF and VEGF receptor	0.01816
GABA (A) receptor activation	0.02052
Opening of calcium channels triggered by depolarization of the presynaptic terminal	0.02052
Other semaphorin interactions	0.02052
Cells and molecules involved in local acute inflammatory response	0.02794
Unblocking of NMDA receptor, glutamate binding and activation	0.02794
Activation of NMDA receptor upon glutamate binding and postsynaptic events	0.02794
cAMP cell motility pathway inferred from amoeba model	0.0307
Interleukin-6 regulation of target genes	0.04795
Long-term depression	0.04795

**KEGG 2021 Human**	**adj. P-value**
ECM-receptor interaction	3.76E-09
ABC transporters	1.04E-08
Arrhythmogenic right ventricular cardiomyopathy	5.02E-07
Protein digestion and absorption	0.000001842
Circadian entrainment	0.000004844
Dilated cardiomyopathy	0.00001193
Hypertrophic cardiomyopathy	0.0001191
PI3K-Akt signaling pathway	0.0004359
Focal adhesion	0.0005138
Long-term depression	0.002048
Glutamatergic synapse	0.002048
cGMP-PKG signaling pathway	0.002048
Calcium signaling pathway	0.002618
Oxytocin signaling pathway	0.004372
Morphine addiction	0.006175
Nicotine addiction	0.009043
Phospholipase D signaling pathway	0.009043
GABAergic synapse	0.01016
Adrenergic signaling in cardiomyocytes	0.01016
Rap1 signaling pathway	0.0124
Aldosterone synthesis and secretion	0.01254
Salivary secretion	0.01432
Cholinergic synapse	0.01432
Mannose type O-glycan biosynthesis	0.01695
Renin secretion	0.01695
cAMP signaling pathway	0.01695
Axon guidance	0.02051
Vitamin digestion and absorption	0.0215
Cortisol synthesis and secretion	0.02192
Other types of O-glycan biosynthesis	0.02268
Retrograde endocannabinoid signaling	0.02268
Insulin secretion	0.02441
Glycerolipid metabolism	0.02815
Apelin signaling pathway	0.02919
Phosphatidylinositol signaling system	0.03763
Cell adhesion molecules	0.03926
Inflammatory mediator regulation of TRP channels	0.03998
**Reactome 2016**	**adj. P-value**
Extracellular matrix organization Homo sapiens R-HSA-1474244	2.64E-12
Interaction between L1 and Ankyrins Homo sapiens R-HSA-445095	8.44E-09
Collagen biosynthesis and modifying enzymes Homo sapiens R-HSA-1650814	8.44E-09
L1CAM interactions Homo sapiens R-HSA-373760	1.04E-08
Axon guidance Homo sapiens R-HSA-422475	3.08E-07
Collagen formation Homo sapiens R-HSA-1474290	5.06E-07
Transmembrane transport of small molecules Homo sapiens R-HSA-382551	9.25E-07
NCAM1 interactions Homo sapiens R-HSA-419037	0.000004117
Neuronal System Homo sapiens R-HSA-112316	0.000004117
Transmission across Chemical Synapses Homo sapiens R-HSA-112315	0.000004204
ABC-family proteins mediated transport Homo sapiens R-HSA-382556	0.00001743
Ion channel transport Homo sapiens R-HSA-983712	0.00001941

O-linked glycosylation Homo sapiens R-HSA-5173105	0.00003716
Effects of PIP2 hydrolysis Homo sapiens R-HSA-114508	0.00005059
Phase 0 - rapid depolarisation Homo sapiens R-HSA-5576892	0.00005059
Muscle contraction Homo sapiens R-HSA-397014	0.00008966
Diseases associated with O-glycosylation of proteins Homo sapiens R-HSA-3906995	0.0001286
Integrin cell surface interactions Homo sapiens R-HSA-216083	0.0001286
ECM proteoglycans Homo sapiens R-HSA-3000178	0.000279
Ion transport by P-type ATPases Homo sapiens R-HSA-936837	0.000279
Cardiac conduction Homo sapiens R-HSA-5576891	0.000279
Nitric oxide stimulates guanylate cyclase Homo sapiens R-HSA-392154	0.0007398
Neurotransmitter Receptor Binding And Downstream Transmission In The Postsynaptic Cell	0.0007526
Assembly of collagen fibrils and other multimeric structures Homo sapiens R-HSA-2022090	0.0007763
Developmental Biology Homo sapiens R-HSA-1266738	0.0007763
Diseases of glycosylation Homo sapiens R-HSA-3781865	0.0008384
Laminin interactions Homo sapiens R-HSA-3000157	0.001679
Defective B3GALTL causes Peters-plus syndrome (PpS) Homo sapiens R-HSA-5083635	0.001786
O-glycosylation of TSR domain-containing proteins Homo sapiens R-HSA-5173214	0.002374
Phase 2 - plateau phase Homo sapiens R-HSA-5576893	0.005246
Degradation of the extracellular matrix Homo sapiens R-HSA-1474228	0.005621
Hemostasis Homo sapiens R-HSA-109582	0.005974
cGMP effects Homo sapiens R-HSA-418457	0.006798
Other semaphorin interactions Homo sapiens R-HSA-416700	0.01007
GABA receptor activation Homo sapiens R-HSA-977443	0.01007
Collagen degradation Homo sapiens R-HSA-1442490	0.01125
Platelet homeostasis Homo sapiens R-HSA-418346	0.01292
Non-integrin membrane-ECM interactions Homo sapiens R-HSA-3000171	0.02248
Unblocking of NMDA receptor, glutamate binding and activation Homo sapiens R-HSA-438066	0.02524
Depolarization of the Presynaptic Terminal Triggers the Opening of Calcium Channels	0.02704
GABA A receptor activation Homo sapiens R-HSA-977441	0.02704
Phase 1 - inactivation of fast Na+ channels Homo sapiens R-HSA-5576894	0.02704
Activation of NMDA receptor upon glutamate binding and postsynaptic events	0.03646
NCAM signaling for neurite out-growth Homo sapiens R-HSA-375165	0.04807
**WikiPathway 2021 Human**	**adj. P-value**
Arrhythmogenic Right Ventricular Cardiomyopathy WP2118	0.000004711
Splicing factor NOVA regulated synaptic proteins WP4148	0.00001292
Phosphodiesterases in neuronal function WP4222	0.0003876
Focal Adhesion-PI3K-Akt-mTOR-signaling pathway WP3932	0.0003876
PI3K-Akt signaling pathway WP4172	0.002477
Focal Adhesion WP306	0.004761
miRNA targets in ECM and membrane receptors WP2911	0.01338
Primary focal segmental glomerulosclerosis (FSGS) WP2572	0.01838
Fragile X Syndrome WP4549	0.03064
Alzheimer's disease WP2059	0.03343
Cell-type Dependent Selectivity of CCK2R Signaling WP3679	0.04197
Cells and molecules involved in local acute inflammatory response WP4493	0.04197
PKC-gamma calcium signaling pathway in ataxia WP4760	0.04197
**NCI-Nature 2016**	**adj. P-value**
Beta1 integrin cell surface interactions Homo sapiens 2fd0bc63-618d-11e5-8ac5-06603eb7f303	0.000003052
Syndecan-1-mediated signaling events Homo sapiens f957cc16-6195-11e5-8ac5-06603eb7f303	0.0001446
Integrins in angiogenesis Homo sapiens 2ddeac89-6194-11e5-8ac5-06603eb7f303	0.0004829

Beta3 integrin cell surface interactions Homo sapiens c2800165-618d-11e5-8ac5-06603eb7f303	0.003116
Plexin-D1 Signaling Homo sapiens e3068f36-6194-11e5-8ac5-06603eb7f303	0.0155
**MSigDB Hallmark 2020**	**adj. P-value**
Epithelial Mesenchymal Transition	9.03E-07
UV Response Dn	0.01206
Myogenesis	0.01206
**Elsevier Pathway Collection**	**adj. P-value**
Proteins Involved in Epilepsy	9.74E-11
Inherited Channelopathies	0.004141
Postsynaptic Neuron Activation	0.004378
Cochlear Hair Cell Stereocilia Proteins Mutations (Age-Related)	0.005433
Proteins Involved in Tangier Disease	0.008267
nNOS (NOS1) and iNOS (NOS2) Activation via Glutamate	0.01508
Proteins Involved in Hearing Loss	0.01883
**Panther 2016**	**adj. P-value**
Integrin signalling pathway Homo sapiens P00034	0.00009407
Metabotropic glutamate receptor group III pathway Homo sapiens P00039	0.003325
Beta2 adrenergic receptor signaling pathway Homo sapiens P04378	0.007017
Beta1 adrenergic receptor signaling pathway Homo sapiens P04377	0.007017
Nicotinic acetylcholine receptor signaling pathway Homo sapiens P00044	0.007791
Oxytocin receptor mediated signaling pathway Homo sapiens P04391	0.01716
Alzheimer disease-amyloid secretase pathway Homo sapiens P00003	0.01716
Endothelin signaling pathway Homo sapiens P00019	0.01716
Histamine H1 receptor mediated signaling pathway Homo sapiens P04385	0.03671
Ionotropic glutamate receptor pathway Homo sapiens P00037	0.04667

Supplementary Table S7. Enrichment Pathway analysis of down-regulated genes.

GO BP	adj. P-value
SRP-dependent cotranslational protein targeting to membrane (GO:0006614)	7.68E-74
cytoplasmic translation (GO:0002181)	1.73E-72
cotranslational protein targeting to membrane (GO:0006613)	3.89E-72
protein targeting to ER (GO:0045047)	5.75E-68
nuclear-transcribed mRNA catabolic process, nonsense-mediated decay (GO:0000184)	5.01E-64
peptide biosynthetic process (GO:0043043)	8.74E-60
translation (GO:0006412)	2.85E-55
nuclear-transcribed mRNA catabolic process (GO:0000956)	9.15E-54
cellular macromolecule biosynthetic process (GO:0034645)	1.36E-53
gene expression (GO:0010467)	1.53E-42
rRNA processing (GO:0006364)	2.54E-36
rRNA metabolic process (GO:0016072)	7.94E-36
ribosome biogenesis (GO:0042254)	1.12E-34
cellular protein metabolic process (GO:0044267)	1.53E-33
ncRNA processing (GO:0034470)	2.08E-31
ribosome assembly (GO:0042255)	1.79E-10
protein-DNA complex assembly (GO:0065004)	3.35E-10
nucleosome organization (GO:0034728)	3.88E-10
chromatin assembly (GO:0031497)	1.51E-09
ribosomal large subunit biogenesis (GO:0042273)	1.59E-09
nucleosome assembly (GO:0006334)	2.07E-09
ribosomal small subunit biogenesis (GO:0042274)	8.36E-09
ribonucleoprotein complex biogenesis (GO:0022613)	3.44E-07
ribonucleoprotein complex assembly (GO:0022618)	5.49E-07
aerobic electron transport chain (GO:0019646)	0.000003051
mitochondrial ATP synthesis coupled electron transport (GO:0042775)	0.000003594
mitochondrial respiratory chain complex I assembly (GO:0032981)	0.000013
NADH dehydrogenase complex assembly (GO:0010257)	0.000013
translational elongation (GO:0006414)	0.00002143
aerobic respiration (GO:0009060)	0.00003741
mitochondrion organization (GO:0007005)	0.00004293
mitochondrial transport (GO:0006839)	0.00005794
ribosomal small subunit assembly (GO:0000028)	0.00009884
ribosomal large subunit assembly (GO:0000027)	0.0001539
cellular response to oxidative stress (GO:0034599)	0.0002514
regulation of gene silencing by RNA (GO:0060966)	0.000299
regulation of posttranscriptional gene silencing (GO:0060147)	0.000299
mitochondrial respiratory chain complex assembly (GO:0033108)	0.0003433
regulation of gene silencing by miRNA (GO:0060964)	0.0003524
establishment of protein localization to mitochondrion (GO:0072655)	0.0005141
regulation of DNA recombination at telomere (GO:0072695)	0.0005725
negative regulation of DNA recombination at telomere (GO:0048239)	0.0005725
regulation of translation (GO:0006417)	0.0005725
cellular respiration (GO:0045333)	0.001046
mitochondrial translational elongation (GO:0070125)	0.001235
mitochondrial translational termination (GO:0070126)	0.001235
positive regulation of viral transcription (GO:0050434)	0.001685
mitochondrial translation (GO:0032543)	0.001685

RNA metabolic process (GO:0016070)	0.001685
translational termination (GO:0006415)	0.002576
nucleic acid metabolic process (GO:0090304)	0.002719
RNA splicing, via transesterification reactions with bulged adenosine as nucleophile (GO:0000377)	0.002909
inner mitochondrial membrane organization (GO:0007007)	0.003178
pentose-phosphate shunt (GO:0006098)	0.00322
hydrogen peroxide metabolic process (GO:0042743)	0.00322
transcription-coupled nucleotide-excision repair (GO:0006283)	0.00322
mitochondrial electron transport, NADH to ubiquinone (GO:0006120)	0.00331
hydrogen peroxide catabolic process (GO:0042744)	0.003584
DNA-dependent DNA replication (GO:0006261)	0.003584
mRNA splicing, via spliceosome (GO:0000398)	0.003584
cellular response to chemical stress (GO:0062197)	0.003607
fibroblast growth factor receptor signaling pathway (GO:0008543)	0.003745
positive regulation of viral process (GO:0048524)	0.003879
gluconeogenesis (GO:0006094)	0.004224
regulation of viral transcription (GO:0046782)	0.004224
positive regulation of signal transduction by p53 class mediator (GO:1901798)	0.004326
regulation of apoptotic process (GO:0042981)	0.004467
nucleotide-excision repair (GO:0006289)	0.004839
7-methylguanosine mRNA capping (GO:0006370)	0.00498
7-methylguanosine RNA capping (GO:0009452)	0.00498
glucose 6-phosphate metabolic process (GO:0051156)	0.005272
negative regulation of ubiquitin-protein transferase activity (GO:0051444)	0.005322
protein targeting to mitochondrion (GO:0006626)	0.005866
hexose biosynthetic process (GO:0019319)	0.006244
negative regulation of ubiquitin protein ligase activity (GO:1904667)	0.006871
DNA unwinding involved in DNA replication (GO:0006268)	0.00693
nuclear transport (GO:0051169)	0.00693
nucleotide catabolic process (GO:0009166)	0.00693
negative regulation of DNA recombination (GO:0045910)	0.007184
mitochondrial ATP synthesis coupled proton transport (GO:0042776)	0.009283
maturation of LSU-rRNA (GO:0000470)	0.009361
TOR signaling (GO:0031929)	0.009361
negative regulation of proteolysis involved in cellular protein catabolic process (GO:1903051)	0.009361
modulation by symbiont of host process (GO:0044003)	0.009927
negative regulation of ubiquitin-dependent protein catabolic process (GO:2000059)	0.01264
glucose metabolic process (GO:0006006)	0.01264
cristae formation (GO:0042407)	0.01357
negative regulation of oxidative stress-induced cell death (GO:1903202)	0.01357
negative regulation of hydrogen peroxide-induced neuron death (GO:1903208)	0.0136
positive regulation of dendritic cell differentiation (GO:2001200)	0.0136
rDNA heterochromatin assembly (GO:0000183)	0.01398
ATP synthesis coupled proton transport (GO:0015986)	0.01423
DNA duplex unwinding (GO:0032508)	0.01423
pre-replicative complex assembly (GO:0036388)	0.0147
glycolytic process (GO:0006096)	0.0152
cellular response to fibroblast growth factor stimulus (GO:0044344)	0.0152
carbohydrate catabolic process (GO:0016052)	0.01801
mitochondrial electron transport, ubiquinol to cytochrome c (GO:0006122)	0.01891
transcription elongation from RNA polymerase II promoter (GO:0006368)	0.01965
regulation of ubiquitin-dependent protein catabolic process (GO:2000058)	0.01991
nucleic acid-templated transcription (GO:0097659)	0.01991
mRNA processing (GO:0006397)	0.01991

regulation of translational initiation (GO:0006446)	0.02107
pyruvate metabolic process (GO:0006090)	0.02139
DNA-templated transcription, elongation (GO:0006354)	0.02301
negative regulation of programmed cell death (GO:0043069)	0.02318
negative regulation of hydrogen peroxide-induced cell death (GO:1903206)	0.02429
regulation of mRNA stability (GO:0043488)	0.02432
positive regulation of gene expression, epigenetic (GO:0045815)	0.02597
negative regulation of apoptotic process (GO:0043066)	0.02635
positive regulation of translation (GO:0045727)	0.02639
postreplication repair (GO:0006301)	0.03124
response to reactive oxygen species (GO:0000302)	0.03124
selective autophagy (GO:0061912)	0.03124
macropinocytosis (GO:0044351)	0.03462
canonical glycolysis (GO:0061621)	0.03462
glucose catabolic process to pyruvate (GO:0061718)	0.03462
heterochromatin assembly (GO:0031507)	0.03462
protein insertion into mitochondrial membrane (GO:0051204)	0.03462
negative regulation of protein kinase activity (GO:0006469)	0.03748
nitric oxide biosynthetic process (GO:0006809)	0.03867
regulation of mRNA catabolic process (GO:0061013)	0.03879
platelet aggregation (GO:0070527)	0.0391
glycolytic process through glucose-6-phosphate (GO:0061620)	0.04029
amyloid fibril formation (GO:1990000)	0.04342
cellular response to reactive oxygen species (GO:0034614)	0.04342
protein targeting (GO:0006605)	0.04578
regulation of chromatin silencing (GO:0031935)	0.04625
nitric oxide metabolic process (GO:0046209)	0.04625
antimicrobial humoral immune response mediated by antimicrobial peptide (GO:0061844)	0.04625
negative regulation of intrinsic apoptotic signaling pathway (GO:2001243)	0.04625
regulation of endodeoxyribonuclease activity (GO:0032071)	0.04707
negative regulation of response to reactive oxygen species (GO:1901032)	0.04707
positive regulation of leukocyte differentiation (GO:1902107)	0.04707
DNA replication initiation (GO:0006270)	0.04744
**GO MF**	**adj. P-value**
RNA binding (GO:0003723)	8.45E-34
cadherin binding (GO:0045296)	6.04E-08
ubiquitin ligase inhibitor activity (GO:1990948)	1.10E-07
ubiquitin-protein transferase inhibitor activity (GO:0055105)	3.61E-07
rRNA binding (GO:0019843)	0.00002258
oxidoreduction-driven active transmembrane transporter activity (GO:0015453)	0.00008511
large ribosomal subunit rRNA binding (GO:0070180)	0.0006068
mRNA 5'-UTR binding (GO:0048027)	0.001437
DNA binding (GO:0003677)	0.004017
NADH dehydrogenase (quinone) activity (GO:0050136)	0.01061
NADH dehydrogenase (ubiquinone) activity (GO:0008137)	0.01061
mRNA binding (GO:0003729)	0.01235
5'-3' RNA polymerase activity (GO:0034062)	0.01923
DNA-directed 5'-3' RNA polymerase activity (GO:0003899)	0.01923
translation initiation factor activity (GO:0003743)	0.02149
ubiquinol-cytochrome-c reductase activity (GO:0008121)	0.02584
single-stranded DNA binding (GO:0003697)	0.02584
damaged DNA binding (GO:0003684)	0.02774

translation elongation factor activity (GO:0003746)	0.03431
muscle alpha-actinin binding (GO:0051371)	0.04316
ubiquitin protein ligase binding (GO:0031625)	0.0457
**GO CC**	**adj. P-value**
large ribosomal subunit (GO:0015934)	4.68E-43
cytosolic large ribosomal subunit (GO:0022625)	9.45E-42
ribosome (GO:0005840)	2.68E-32
small ribosomal subunit (GO:0015935)	6.90E-31
cytosolic small ribosomal subunit (GO:0022627)	7.91E-30
polysomal ribosome (GO:0042788)	2.57E-22
focal adhesion (GO:0005925)	3.86E-19
cell-substrate junction (GO:0030055)	8.33E-19
mitochondrial membrane (GO:0031966)	3.51E-15
nucleus (GO:0005634)	4.86E-12
mitochondrial inner membrane (GO:0005743)	1.08E-11
organelle inner membrane (GO:0019866)	1.53E-11
intracellular membrane-bounded organelle (GO:0043231)	8.36E-10
intracellular non-membrane-bounded organelle (GO:0043232)	7.13E-08
cytoplasmic vesicle lumen (GO:0060205)	6.69E-07
nuclear lumen (GO:0031981)	0.00001237
nucleolus (GO:0005730)	0.0000157
mitochondrial outer membrane (GO:0005741)	0.00005341
mitochondrial proton-transporting ATP synthase complex, catalytic sector F(1) (GO:0000275)	0.0001168
RNA polymerase II, core complex (GO:0005665)	0.0001168
ficolin-1-rich granule lumen (GO:1904813)	0.0001374
cytoskeleton (GO:0005856)	0.0001374
organelle outer membrane (GO:0031968)	0.0002107
rough endoplasmic reticulum membrane (GO:0030867)	0.0002733
secretory granule lumen (GO:0034774)	0.0004765
mitochondrial matrix (GO:0005759)	0.0009539
mitochondrial respiratory chain complex I (GO:0005747)	0.001147
respiratory chain complex I (GO:0045271)	0.001147
proton-transporting ATP synthase complex (GO:0045259)	0.001292
ficolin-1-rich granule (GO:0101002)	0.001357
mitochondrial proton-transporting ATP synthase complex (GO:0005753)	0.003147
postsynaptic density (GO:0014069)	0.004591
cytoplasmic side of endoplasmic reticulum membrane (GO:0098554)	0.007704
asymmetric synapse (GO:0032279)	0.009474
spliceosomal snRNP complex (GO:0097525)	0.0183
mitochondrial respiratory chain complex III (GO:0005750)	0.02501
U2-type spliceosomal complex (GO:0005684)	0.03747
mitochondrial outer membrane translocase complex (GO:0005742)	0.0443
polymeric cytoskeletal fiber (GO:0099513)	0.0443
**Panther 2016**	**adj. P-value**
Glycolysis Homo sapiens P00024	0.02279
Cytoskeletal regulation by Rho GTPase Homo sapiens P00016	0.04118
**HumanCyc 2016**	**adj. P-value**
gluconeogenesis Homo sapiens PWY66-399	0.001102

pentose phosphate pathway Homo sapiens PENTOSE-P-PWY	0.001806
superpathway of conversion of glucose to acetyl CoA and entry into the TCA cycle Homo sapiens PWY66-407	0.005055
glutathione redox reactions I Homo sapiens PWY-4081	0.01416
glycolysis Homo sapiens PWY66-400	0.0196
**Reactome 2016**	**adj. P-value**
Eukaryotic Translation Elongation Homo sapiens R-HSA-156842	8.32E-81
Peptide chain elongation Homo sapiens R-HSA-156902	2.31E-80
Viral mRNA Translation Homo sapiens R-HSA-192823	1.90E-76
L13a-mediated translational silencing of Ceruloplasmin expression Homo sapiens R-HSA-156827	6.10E-76
3' -UTR-mediated translational regulation Homo sapiens R-HSA-157279	6.10E-76
Cap-dependent Translation Initiation Homo sapiens R-HSA-72737	6.10E-76
Eukaryotic Translation Initiation Homo sapiens R-HSA-72613	6.10E-76
Nonsense Mediated Decay (NMD) independent of the Exon Junction Complex (EJC) Homo sapiens R-HSA-975956	8.88E-76
Formation of a pool of free 40S subunits Homo sapiens R-HSA-72689	9.34E-76
GTP hydrolysis and joining of the 60S ribosomal subunit Homo sapiens R-HSA-72706	9.34E-76
Selenocysteine synthesis Homo sapiens R-HSA-2408557	3.43E-75
Eukaryotic Translation Termination Homo sapiens R-HSA-72764	3.43E-75
Translation Homo sapiens R-HSA-72766	7.65E-71
Influenza Viral RNA Transcription and Replication Homo sapiens R-HSA-168273	4.01E-69
Nonsense-Mediated Decay (NMD) Homo sapiens R-HSA-927802	9.61E-68
Nonsense Mediated Decay (NMD) enhanced by the Exon Junction Complex (EJC) Homo sapiens R-HSA-975957	9.61E-68
SRP-dependent cotranslational protein targeting to membrane Homo sapiens R-HSA-1799339	2.34E-67
Influenza Life Cycle Homo sapiens R-HSA-168255	1.45E-66
Influenza Infection Homo sapiens R-HSA-168254	3.11E-66
Selenoamino acid metabolism Homo sapiens R-HSA-2408522	7.68E-66
Major pathway of rRNA processing in the nucleolus Homo sapiens R-HSA-6791226	4.19E-53
rRNA processing Homo sapiens R-HSA-72312	3.04E-50
Infectious disease Homo sapiens R-HSA-5663205	3.60E-48
Metabolism of amino acids and derivatives Homo sapiens R-HSA-71291	9.54E-41
Activation of the mRNA upon binding of the cap-binding complex and eIFs, and subsequent binding to 43S Homo sapiens R-HSA-72662	6.22E-31
Translation initiation complex formation Homo sapiens R-HSA-72649	9.65E-30
Formation of the ternary complex, and subsequently, the 43S complex Homo sapiens R-HSA-72695	4.20E-29
Ribosomal scanning and start codon recognition Homo sapiens R-HSA-72702	3.02E-28
Disease Homo sapiens R-HSA-1643685	7.47E-27
Metabolism Homo sapiens R-HSA-1430728	1.52E-22
Metabolism of proteins Homo sapiens R-HSA-392499	1.73E-21
Gene Expression Homo sapiens R-HSA-74160	2.56E-17
Respiratory electron transport Homo sapiens R-HSA-611105	5.10E-07
The citric acid (TCA) cycle and respiratory electron transport Homo sapiens R-HSA-1428517	0.00000124
Respiratory electron transport, ATP synthesis by chemiosmotic coupling, and heat production by uncoupling proteins.	0.000002351
Detoxification of Reactive Oxygen Species Homo sapiens R-HSA-3299685	0.000004666
FGFR2 alternative splicing Homo sapiens R-HSA-6803529	0.00006925
Signaling by FGFR2 IIIa TM Homo sapiens R-HSA-8851708	0.00007302
Pentose phosphate pathway (hexose monophosphate shunt) Homo sapiens R-HSA-71336	0.0001736
HIV Infection Homo sapiens R-HSA-162906	0.0001858
Complex I biogenesis Homo sapiens R-HSA-6799198	0.0002111
Nucleotide Excision Repair Homo sapiens R-HSA-5696398	0.0002111
TP53 Regulates Metabolic Genes Homo sapiens R-HSA-5628897	0.0002438
MicroRNA (miRNA) biogenesis Homo sapiens R-HSA-203927	0.0002637
Abortive elongation of HIV-1 transcript in the absence of Tat Homo sapiens R-HSA-167242	0.0002637

Gluconeogenesis Homo sapiens R-HSA-70263	0.0003084
mRNA Splicing - Minor Pathway Homo sapiens R-HSA-72165	0.0003227
Metabolism of nucleotides Homo sapiens R-HSA-15869	0.0003227
Transcriptional Regulation by TP53 Homo sapiens R-HSA-3700989	0.0007296
RNA Pol II CTD phosphorylation and interaction with CE Homo sapiens R-HSA-167160	0.0007413
RNA Pol II CTD phosphorylation and interaction with CE Homo sapiens R-HSA-77075	0.0007413
Mitochondrial translation initiation Homo sapiens R-HSA-5368286	0.0009854
mRNA Capping Homo sapiens R-HSA-72086	0.001176
Dual incision in TC-NER Homo sapiens R-HSA-6782135	0.001585
Transcription-Coupled Nucleotide Excision Repair (TC-NER) Homo sapiens R-HSA-6781827	0.001672
Pink/Parkin Mediated Mitophagy Homo sapiens R-HSA-5205685	0.001693
Mitophagy Homo sapiens R-HSA-5205647	0.001693
Pausing and recovery of Tat-mediated HIV elongation Homo sapiens R-HSA-167238	0.001693
Tat-mediated HIV elongation arrest and recovery Homo sapiens R-HSA-167243	0.001693
Mitochondrial translation Homo sapiens R-HSA-5368287	0.001693
Formation of TC-NER Pre-Incision Complex Homo sapiens R-HSA-6781823	0.00183
mRNA Splicing - Major Pathway Homo sapiens R-HSA-72163	0.001847
HIV elongation arrest and recovery Homo sapiens R-HSA-167287	0.001878
Pausing and recovery of HIV elongation Homo sapiens R-HSA-167290	0.001878
Elongation arrest and recovery Homo sapiens R-HSA-112387	0.001878
Mitotic G1-G1/S phases Homo sapiens R-HSA-453279	0.00205
Formation of the Early Elongation Complex Homo sapiens R-HSA-113418	0.002175
Formation of the HIV-1 Early Elongation Complex Homo sapiens R-HSA-167158	0.002175
FGFR2 mutant receptor activation Homo sapiens R-HSA-1839126	0.002175
S Phase Homo sapiens R-HSA-69242	0.002486
Synthesis of DNA Homo sapiens R-HSA-69239	0.002947
G1/S Transition Homo sapiens R-HSA-69206	0.003079
Mitochondrial translation termination Homo sapiens R-HSA-5419276	0.003089
Mitochondrial translation elongation Homo sapiens R-HSA-5389840	0.003089
mRNA Splicing Homo sapiens R-HSA-72172	0.003397
RHO GTPases Activate WASPs and WAVEs Homo sapiens R-HSA-5663213	0.00346
Host Interactions of HIV factors Homo sapiens R-HSA-162909	0.00346
Sema4D in semaphorin signaling Homo sapiens R-HSA-400685	0.004347
Gap-filling DNA repair synthesis and ligation in TC-NER Homo sapiens R-HSA-6782210	0.004818
Ubiquitin-dependent degradation of Cyclin D1 Homo sapiens R-HSA-69229	0.004984
Ubiquitin-dependent degradation of Cyclin D Homo sapiens R-HSA-75815	0.004984
APOBEC3G mediated resistance to HIV-1 infection Homo sapiens R-HSA-180689	0.005227
DNA Replication Homo sapiens R-HSA-69306	0.005284
PIWI-interacting RNA (piRNA) biogenesis Homo sapiens R-HSA-5601884	0.006084
Glucose metabolism Homo sapiens R-HSA-70326	0.006696
HIV Life Cycle Homo sapiens R-HSA-162587	0.007585
AUF1 (hnRNP D0) binds and destabilizes mRNA Homo sapiens R-HSA-450408	0.007725
Mitochondrial protein import Homo sapiens R-HSA-1268020	0.007725
Assembly of the pre-replicative complex Homo sapiens R-HSA-68867	0.007725
Viral Messenger RNA Synthesis Homo sapiens R-HSA-168325	0.007901
DNA Replication Pre-Initiation Homo sapiens R-HSA-69002	0.008324
M/G1 Transition Homo sapiens R-HSA-68874	0.008324
HIV Transcription Elongation Homo sapiens R-HSA-167169	0.008571
Tat-mediated elongation of the HIV-1 transcript Homo sapiens R-HSA-167246	0.008571
Formation of HIV-1 elongation complex containing HIV-1 Tat Homo sapiens R-HSA-167200	0.008571
Signaling by FGFR2 in disease Homo sapiens R-HSA-5655253	0.008571
DNA Repair Homo sapiens R-HSA-73894	0.00858
mTORC1-mediated signalling Homo sapiens R-HSA-166208	0.008637
Oncogene Induced Senescence Homo sapiens R-HSA-2559585	0.008637

Formation of HIV elongation complex in the absence of HIV Tat Homo sapiens R-HSA-167152	0.008637
RNA Polymerase II Transcription Elongation Homo sapiens R-HSA-75955	0.008637
Formation of RNA Pol II elongation complex Homo sapiens R-HSA-112382	0.008637
RNA Polymerase II HIV Promoter Escape Homo sapiens R-HSA-167162	0.008637
RNA Polymerase II Promoter Escape Homo sapiens R-HSA-73776	0.008637
RNA Polymerase II Transcription Initiation And Promoter Clearance Homo sapiens R-HSA-76042	0.008637
RNA Polymerase II Transcription Initiation Homo sapiens R-HSA-75953	0.008637
RNA Polymerase II Transcription Pre-Initiation And Promoter Opening Homo sapiens R-HSA-73779	0.008637
HIV Transcription Initiation Homo sapiens R-HSA-167161	0.008637
Switching of origins to a post-replicative state Homo sapiens R-HSA-69052	0.008637
Orc1 removal from chromatin Homo sapiens R-HSA-68949	0.008637
Regulation of mRNA stability by proteins that bind AU-rich elements Homo sapiens R-HSA-450531	0.01001
Removal of licensing factors from origins Homo sapiens R-HSA-69300	0.0102
RHO GTPase Effectors Homo sapiens R-HSA-195258	0.0102
Early Phase of HIV Life Cycle Homo sapiens R-HSA-162594	0.01075
Purine metabolism Homo sapiens R-HSA-73847	0.01075
SCF(Skp2)-mediated degradation of p27/p21 Homo sapiens R-HSA-187577	0.01075
Sema4D induced cell migration and growth-cone collapse Homo sapiens R-HSA-416572	0.01233
Regulation of DNA replication Homo sapiens R-HSA-69304	0.01308
PCP/CE pathway Homo sapiens R-HSA-4086400	0.01439
Signaling by FGFR in disease Homo sapiens R-HSA-1226099	0.01597
Spry regulation of FGF signaling Homo sapiens R-HSA-1295596	0.01725
RHO GTPases activate CIT Homo sapiens R-HSA-5625900	0.01725
TGF-beta receptor signaling in EMT (epithelial to mesenchymal transition) Homo sapiens R-HSA-2173791	0.01725
DNA Damage Recognition in GG-NER Homo sapiens R-HSA-5696394	0.01794
Cellular responses to stress Homo sapiens R-HSA-2262752	0.01794
RHO GTPases Activate ROCKs Homo sapiens R-HSA-5627117	0.02138
Global Genome Nucleotide Excision Repair (GG-NER) Homo sapiens R-HSA-5696399	0.02207
Toll Like Receptor 10 (TLR10) Cascade Homo sapiens R-HSA-168142	0.02207
Toll Like Receptor 5 (TLR5) Cascade Homo sapiens R-HSA-168176	0.02207
MyD88 cascade initiated on plasma membrane Homo sapiens R-HSA-975871	0.02207
RHO GTPases Activate Formins Homo sapiens R-HSA-5663220	0.02243
Cyclin E associated events during G1/S transition Homo sapiens R-HSA-69202	0.02343
TRAF6 mediated induction of NFkB and MAP kinases upon TLR7/8 or 9 activation Homo sapiens R-HSA-975138	0.02343
Displacement of DNA glycosylase by APEX1 Homo sapiens R-HSA-110357	0.02388
Integration of provirus Homo sapiens R-HSA-162592	0.02388
Cell Cycle Homo sapiens R-HSA-1640170	0.02464
Cyclin A:Cdk2-associated events at S phase entry Homo sapiens R-HSA-69656	0.02491
Cell Cycle, Mitotic Homo sapiens R-HSA-69278	0.02526
MyD88 dependent cascade initiated on endosome Homo sapiens R-HSA-975155	0.02613
Toll Like Receptor 7/8 (TLR7/8) Cascade Homo sapiens R-HSA-168181	0.02613
Transcriptional regulation of pluripotent stem cells Homo sapiens R-HSA-452723	0.0267
Termination of translesion DNA synthesis Homo sapiens R-HSA-5656169	0.0284
Formation of Incision Complex in GG-NER Homo sapiens R-HSA-5696395	0.02966
Purine catabolism Homo sapiens R-HSA-74259	0.03111
Toll Like Receptor 9 (TLR9) Cascade Homo sapiens R-HSA-168138	0.03178
CDT1 association with the CDC6:ORC:origin complex Homo sapiens R-HSA-68827	0.03271
Processing of Capped Intron-Containing Pre-mRNA Homo sapiens R-HSA-72203	0.03284
DNA strand elongation Homo sapiens R-HSA-69190	0.03601
Glycolysis Homo sapiens R-HSA-70171	0.03601
Oxidative Stress Induced Senescence Homo sapiens R-HSA-2559580	0.03825
MyD88:Mal cascade initiated on plasma membrane Homo sapiens R-HSA-166058	0.03999
Toll Like Receptor TLR1:TLR2 Cascade Homo sapiens R-HSA-168179	0.03999
Toll Like Receptor TLR6:TLR2 Cascade Homo sapiens R-HSA-168188	0.03999

Toll Like Receptor 2 (TLR2) Cascade Homo sapiens R-HSA-181438	0.03999
TP53 Regulates Transcription of DNA Repair Genes Homo sapiens R-HSA-6796648	0.04068
Beta-catenin independent WNT signaling Homo sapiens R-HSA-3858494	0.04099
Apoptosis Homo sapiens R-HSA-109581	0.04275
Late Phase of HIV Life Cycle Homo sapiens R-HSA-162599	0.04443
Post-chaperonin tubulin folding pathway Homo sapiens R-HSA-389977	0.04502
Activated TLR4 signalling Homo sapiens R-HSA-166054	0.04754
Unwinding of DNA Homo sapiens R-HSA-176974	0.04799
Programmed Cell Death Homo sapiens R-HSA-5357801	0.04799
Base Excision Repair Homo sapiens R-HSA-73884	0.04818
Resolution of Abasic Sites (AP sites) Homo sapiens R-HSA-73933	0.04818
Regulation of activated PAK-2p34 by proteasome mediated degradation Homo sapiens R-HSA-211733	0.04899
RNA Polymerase II Pre-transcription Events Homo sapiens R-HSA-674695	0.04923
**MSigDB Hallmark 2020**	**adj. P-value**
Myc Targets V1	1.53E-19
Oxidative Phosphorylation	5.35E-14
Reactive Oxygen Species Pathway	0.000002431
DNA Repair	0.0001292
mTORC1 Signaling	0.001529
Fatty Acid Metabolism	0.002001
E2F Targets	0.02136
TGF-beta Signaling	0.02141
Myc Targets V2	0.02848
Apoptosis	0.03245
Adipogenesis	0.03245
**Elsevier Pathway Collection**	**adj. P-value**
Glycolysis Activation in Cancer (Warburg Effect)	0.01152
**KEGG 2021 Human**	**adj. P-value**
Ribosome	4.90E-61
Coronavirus disease	2.92E-41
Neutrophil extracellular trap formation	1.20E-17
Systemic lupus erythematosus	1.37E-16
Alcoholism	6.58E-14
Parkinson disease	3.81E-12
Huntington disease	1.22E-10
Amyotrophic lateral sclerosis	2.29E-08
Diabetic cardiomyopathy	5.08E-08
Prion disease	1.97E-07
Oxidative phosphorylation	4.03E-07
Thermogenesis	7.54E-07
Pathways of neurodegeneration	0.000001119
Viral carcinogenesis	0.000008901
Alzheimer disease	0.00004432
Necroptosis	0.0003722
Non-alcoholic fatty liver disease	0.0008775
Pentose phosphate pathway	0.001212
Salmonella infection	0.001891
Base excision repair	0.002079

DNA replication	0.003515
Shigellosis	0.003667
RNA polymerase	0.008965
Cysteine and methionine metabolism	0.02358
Pathogenic Escherichia coli infection	0.02656
Glycolysis / Gluconeogenesis	0.02913
Regulation of actin cytoskeleton	0.02913
Hepatocellular carcinoma	0.03392
Glyoxylate and dicarboxylate metabolism	0.03854
Glutathione metabolism	0.04003
**WikiPathway 2021 Human**	**adj. P-value**
Cytoplasmic Ribosomal Proteins WP477	2.56E-73
Electron Transport Chain (OXPHOS system in mitochondria) WP111	3.69E-09
Metabolic reprogramming in colon cancer WP4290	1.96E-08
Translation Factors WP107	2.80E-07
VEGFA-VEGFR2 Signaling Pathway WP3888	2.80E-07
Cori Cycle WP1946	0.00008174
Computational Model of Aerobic Glycolysis WP4629	0.0001041
Glycolysis in senescence WP5049	0.001253
Glycolysis and Gluconeogenesis WP534	0.001384
Pentose Phosphate Metabolism WP134	0.001906
Oxidative phosphorylation WP623	0.001906
Pyrimidine metabolism WP4022	0.001906
Nonalcoholic fatty liver disease WP4396	0.001906
Base Excision Repair WP4752	0.00302
Pathogenic Escherichia coli infection WP2272	0.004293
Mitochondrial complex I assembly model OXPHOS system WP4324	0.004641
Leptin signaling pathway WP2034	0.01051
Pathways in clear cell renal cell carcinoma WP4018	0.02421
Retinoblastoma gene in cancer WP2446	0.02747
HIF1A and PPARG regulation of glycolysis WP2456	0.04516
Mitochondrial complex III assembly WP4921	0.04516
**BioPlanet 2019**	**adj. P-value**
Cytoplasmic ribosomal proteins	5.66E-71
Influenza viral RNA transcription and replication	5.66E-71
Translation	1.03E-68
Influenza infection	1.44E-65
Cap-dependent translation initiation	2.89E-47
Protein metabolism	9.97E-45
Activation of mRNA upon binding of the cap-binding complex and eIFs, and subsequent binding to 43S	9.73E-31
Disease	1.81E-27
Gene expression	7.44E-22
Huntington's disease	7.86E-08
T cell receptor regulation of apoptosis	1.33E-07
Parkinson's disease	1.83E-07
Tricarboxylic acid (TCA) cycle and respiratory electron transport	6.54E-07
Translation factors	0.000001318
Respiratory electron transport, ATP biosynthesis by chemiosmotic coupling, and heat production by uncoupling proteins	0.000002071
Electron transport chain	0.000002782

Viral messenger RNA biosynthesis	0.00001282
HIV infection	0.0002417
Nucleotide excision repair	0.0003444
Gluconeogenesis	0.0005393
Alzheimer's disease	0.0006635
Messenger RNA splicing: minor pathway	0.000719
Regulatory RNA pathways	0.0009311
Pentose phosphate pathway	0.001484
Oxidative phosphorylation	0.001484
Nucleotide metabolism	0.001602
Dual incision reaction in TC-NER	0.001711
Messenger RNA capping	0.001711
S phase	0.00195
Pyrimidine metabolism	0.002272
DNA replication	0.002421
DNA repair	0.003534
Destabilization of mRNA by AUF1 (hnRNP D0)	0.003693
Mitotic G1-G1/S phases	0.003693
Pathogenic Escherichia coli infection	0.005573
Base excision repair	0.00604
HIV factor interactions with host	0.006448
Purine metabolism	0.008818
Glycolysis and gluconeogenesis	0.009165
HIV life cycle	0.009165
APOBEC3G-mediated resistance to HIV-1 infection	0.01018
Mammalian target of rapamycin complex 1 (mTORC1)-mediated signaling	0.01018
HIV-1 transcription initiation	0.01018
RNA polymerase	0.01114
Sema4D in semaphorin signaling	0.01114
Mitochondrial protein import	0.01132
HIV life cycle early phase	0.01901
Myc active pathway	0.02075
mRNA stability regulation by proteins that bind AU-rich elements	0.02226
Signaling events mediated by PRL	0.02311
DNA replication pre-Initiation	0.02554
Glucose metabolism	0.02823
Leptin signaling pathway	0.02823
Licensing factor removal from origins	0.02823
Capped intron-containing pre-mRNA processing	0.02878
Sprouty regulation of FGF signaling	0.0359
TGF-beta receptor signaling in EMT (epithelial to mesenchymal transition)	0.0359
Ras signaling pathway	0.0359
Integration of provirus	0.03613
S6K1 signaling	0.03613
Spliceosome	0.03742
Glycolysis	0.0396
Eukaryotic protein translation	0.04197

Supplementary Table S8. Modules identified in the network by WGCNA functions and their relative size (number of genes).

Module	Genes
black	233
blue	4073
brown	437
cyan	120
green	282
greenyellow	138
grey60	98
lightcyan	115
lightgreen	93
lightyellow	41
magenta	184
midnightblue	115
pink	231
purple	155
red	257
salmon	127
tan	131
turquoise	5018
yellow	361

Supplementary Table S9. This table is already filtered to report piRNAs predicted to be strongly associated with breast cancer according to the iPiDi-PUL tool. The top part of the table shows the piRNAs that were down regulated and the bottom part shows the up regulated in EVs after our eribulin treatments.

piRNA Name	log_2(FoldChange)	iPiDi-PUL score
piR-hsa-12789	-7.417	0.84
piR-hsa-25046	-6.671	0.8475
piR-hsa-1338	1.454	0.8975
piR-hsa-24000	1.554	0.8425
piR-hsa-963	1.988	0.815
piR-hsa-26039	2.325	0.885

Supplementary Table S10. Here we report the tRNA fragments for which we have both expression data derived from this study and those derived from the tRFtarget 2.0 database. The last five columns report the values in terms of log2FC with adjusted p-values. n.s.: not significant.

Name	Eribulin-treated EVs vs control	Tumor vs Normal	LumA vs Normal	Her2 vs Normal	LumB vs Normal	Triple-Negative vs Normal
tRFdb-5026a	1.759	0.803 (p=1.2E-04)	0.940 (p=6.8E-06)	0.781 (p=9.5E-03)	1.286 (p=2.6E-08)	n.s.
tRFdb-5020a	1.023	0.327 (p=4.9E-02)	0.489 (p=9.9E-03)	0.807 (p=2.2E-03)	0.642 (p=2.4E-03)	n.s.
tRFdb-3004a	-4.73	0.557 (p=3.6E-04)	0.604 (p=1.4E-04)	1.007 (p=3.2E-06)	0.693 (p=9.4E-05)	0.871 (p=8.5E-07)
ts-112	-4.833	n.s.	0.434 (p=5.8E-03)	n.s.	0.403 (p=2.1E-02)	n.s.
